# Differential Metabolism of a Two-Carbon Substrate by Members of the *Paracoccidioides* Genus

**DOI:** 10.3389/fmicb.2017.02308

**Published:** 2017-11-27

**Authors:** Lilian C. Baeza, Fabiana R. da Mata, Laurine L. Pigosso, Maristela Pereira, Gustavo H. M. F. de Souza, Alexandre S. G. Coelho, Célia M. de Almeida Soares

**Affiliations:** ^1^Laboratório de Biologia Molecular, Instituto de Ciências Biológicas, Universidade Federal de Goiás, Goiânia, Brazil; ^2^Centro de Ciências Médicas e Farmacêuticas, Universidade Estadual do Oeste do Paraná, Cascavel, Brazil; ^3^Mass Spectrometry Applications Research & Development Laboratory, Waters Corporation, São Paulo, Brazil; ^4^Laboratório de Genética e Genômica de Plantas, Escola de Agronomia, Universidade Federal de Goiás, Goiânia, Brazil

**Keywords:** *Paracoccidioides* spp., proteomic, two-carbon source, sodium acetate, metabolism

## Abstract

The genus *Paracoccidioides* comprises known fungal pathogens of humans and can be isolated from different infection sites. Metabolic peculiarities in different members of the *Paracoccidioides* led us to perform proteomic studies in the presence of the two-carbon molecule acetate, which predominates in the nutrient-poor environment of the phagosome. To investigate the expression rates of proteins of different members of *Paracoccidioides*, including one isolate of *P. lutzii* (*Pb*01) and three isolates of *P. brasiliensis* (*Pb*03, *Pb*339, and *Pb*EPM83), using sodium acetate as a carbon source, proteins were quantified using label-free and data-independent liquid chromatography-mass spectrometry. Protein profiles of the isolates were statistically analyzed, revealing proteins that were differentially expressed when the fungus was cultivated in a non-preferential carbon source rather than glucose. A total of 1,160, 1,211, 1,280, and 1,462 proteins were reproducibly identified and relatively quantified in *P. lutzii* and the *P. brasiliensis* isolates *Pb*03, *Pb*339, and *Pb*EPM83, respectively. Notably, 526, 435, 744, and 747 proteins were differentially expressed among *P. lutzii* and the *P. brasiliensis* isolates *Pb*03, *Pb*339, and *Pb*EPM*83*, respectively, with a fold-change equal to or higher than 1.5. This analysis revealed that reorganization of metabolism occurred through the induction of proteins related to gluconeogenesis, glyoxylic/glyoxylate cycle, response to stress, and degradation of amino acids in the four isolates. The following differences were observed among the isolates: higher increases in the expression levels of proteins belonging to the TCA and respiratory chain in *Pb*EPM83 and *Pb*01; increase in ethanol production in *Pb*01; utilization of cell wall components for gluconeogenesis in *Pb*03 and *Pb*EPM83; and increased β-oxidation and methylcitrate cycle proteins in *Pb*01and *Pb*EPM83. Proteomic profiles indicated that the four isolates reorganized their metabolism in different manners to use acetate as a carbon source.

## Introduction

The genus *Paracoccidioides* includes fungal pathogens causing paracoccidioidomycosis, a disease geographically restricted to subtropical areas of Latin America (San-Blas et al., 2002). Approximately 80% of reported cases of paracoccidioidomycosis occurred in Brazil, followed by Colombia, Venezuela, and Argentina (Martinez, 2015). This genus comprises thermally dimorphic fungi that grow in a hyphal form in the environment, but exist as budding yeast in mammalian hosts. Infection is initiated by inhalation of fungal propagules, which differentiate into the yeast form after reaching the alveolar epithelium (Restrepo et al., 2001).

Multilocus sequencing led to the proposal of four cryptic studies in the genus *Paracoccidioides*: S1, PS2, and PS3 in the *P. brasiliensis* complex and *P. lutzii*, a single monophyletic population (Matute et al., 2006a; Teixeira et al., 2009). Additionally, a new lineage PS4 was proposed, belonging to a region in Venezuela (Teixeira et al., 2014). Recently, the S1 group was divided into lineages S1a and S1b. Dating time analysis to determine the separation of these lineages indicated that S1b was the earliest diverging lineage of *P. brasiliensis* (Muñoz et al., 2016).

The genomes of *Paracoccidioides* have been widely examined. A reduced number of genes was found to be involved in the metabolism of carbohydrates and proteins, as well as in the synthesis of secondary metabolites (Desjardins et al., 2011; Muñoz et al., 2014). Additionally, the genomes of 31 isolates representing lineages S1, PS2, PS3, and PS4 were sequenced, providing new reference genomes for two lineages from PS3 and PS4 (Muñoz et al., 2016). Further functional analysis can provide more data regarding fungus biology.

To grow, a pathogen must assimilate carbon and have metabolic flexibility to assimilate available nutrients in different host niches. Data from transcriptional and proteomic profiles are consistent with this assumption. It has been shown that *Candida albicans* requires isocitrate lyase (ICL), a key enzyme for virulence, in the glyoxylate cycle (Lorenz and Fink, 2001). Because ICL is repressed by glucose, its expression in the host is restricted to the nutrient-limited environment of the phagosome of immune cells (Barelle et al., 2006). *Paracoccidioides* regulate their genes by using different carbon sources. Upon carbon starvation, *P. lutzii* yeast cells shift the metabolism to gluconeogenesis and ethanol production, which is supported by the degradation of amino acids and fatty acids and by modulation of the glyoxylate and tricarboxylic cycles (Lima et al., 2014). During macrophage infection, *P. brasiliensis* positively regulates proteins involved in alternative carbon metabolism, such as those related to gluconeogenesis, beta oxidation, and amino acids catabolism (Parente-Rocha et al., 2015). During the initial stages of lung infection, *P. brasiliensis* alters the expression of genes related to several functional categories including energy metabolism and cell wall metabolism. *Paracoccidioides brasiliensis* remodels cellular lipid metabolism to catabolize its own lipid stores via beta oxidation and the glyoxylate cycle, which are strongly induced during the first 6 h of lung infection (Pigosso et al., 2017).

Proteomic characterization of *Paracoccidioides* was performed by 2D electrophoresis and mass spectrometry. Comparison of the protein expression profiles from yeast cells using glucose as a carbon source revealed different metabolic aspects among members of four phylogenetic species (Pigosso et al., 2013). *Paracoccidioides lutzii* preferentially used anaerobic pathways for energy production; *P. lutzii* and *P. brasiliensis* (*Pb*2 and *Pb*339) showed a better response to reactive oxygen species (ROS) compared to *Pb*EPM83, and *Pb*2 showed a higher abundance of enzymes from the pentose phosphate pathway compared to the others. Additionally, antigenic proteins were differentially accumulated (Pigosso et al., 2013). Results of comparative proteomic analyses were confirmed by biochemical assays (Pigosso et al., 2013). The hierarchical clustering of proteins reflects the phylogenetic relationships among the studied *Paracoccidioides* species, where S1 is an independent species (*Pb*339), PS3 (*Pb*EPM83) is phylogenetically closer to S1 than PS2 (*Pb*2), and *P. lutzii* (*Pb*01) is highly divergent from the other phylogenetic species (Matute et al., 2006a,b; Teixeira et al., 2009).

Macrophages are important components of the innate immune system and include neutrophils, which are front-line phagocytes that eliminate pathogens. During infection by *Paracoccidioides* spp., alveolar macrophages constitute one of the primary defense mechanisms (Brummer et al., 1988). It is generally thought that the phagosome is a nutrient-poor environment (Haas, 2007) whose carbon sources are derived from the breakdown of fatty acids via beta-oxidation, producing acetyl-CoA (Lorenz and Fink, 2002).

Metabolic peculiarities in different members of the *Paracoccidioides* genus, as described above, led us to investigate their metabolism in the presence of acetate, a two-carbon molecule. Acetate utilization requires tricarboxylic acid (TCA) shunting through the glyoxylate cycle, which allows the utilization of two-carbon sources. In this study, we examined *Pb*339 (ATCC 200273), *Pb*03, *Pb*EPM83, and *P. lutzii* (ATCC-MYA-826) (Carrero et al., 2008; Teixeira et al., 2009) to investigate the metabolic utilization of two-carbon sources by these organisms.

## Materials and methods

### Strains and growth conditions

*Paracoccidioides brasiliensis Pb*339 (ATCC 200273), *Pb*03, *Pb*EPM83 (Matute et al., 2006a), and *P. lutzii* (ATCC-MYA-826) (Carrero et al., 2008; Teixeira et al., 2009) were used in this study. Fungi were maintained in brain heart infusion (BHI) medium supplemented with 4% (w/v) glucose at 36°C to cultivate the yeast form. After 3 days of incubation in BHI liquid medium with agitation at 150 rpm, the yeast cells were washed twice with 1X phosphate-buffered saline (1X PBS; 1.4 mM KH_2_PO_4_, 8 mM Na_2_HPO_4_, 140 mM NaCl, 2.7 mM KCl; pH 7.2) and inoculated in chemically defined McVeigh/Morton medium (Restrepo and Jiménez, 1980). Glucose (100 mM) (control) or sodium acetate (100 mM) (test) was added to the defined medium as carbon sources and the cells were grown for 48 h at 36°C with agitation at 150 rpm.

### ICL activity

Following *Paracoccidioides Pb*01, *Pb*03, *Pb*339, and *Pb*EPM83 growth in the presence of acetate and glucose (both 100 mM), the cells were centrifuged at 1,500 × *g*, resuspended in a solution containing 20 mM Tris-HCl, pH 8.8, and 2 mM CaCl_2_ (Fonseca et al., 2001) and disrupted using glass beads and bead beater apparatus (BioSpec, Bartlesville, OK, USA) for 5 cycles of 30 s each while on ice. The cell lysate was centrifuged at 10,000 × *g* for 15 min at 4°C and the supernatant was quantified using Bradford reagent (Sigma-Aldrich, St. Louis, MO, USA) (Bradford, 1976). The amount of protein used was 50 μg (Lima et al., 2014).

ICL activity was determined by measuring the formation of glyoxylate as its phenylhydrazone derivative (Ebel et al., 2006). Glyoxylate-phenylhydrazone formation was determined by measuring the absorbance at 324 nm with an extinction coefficient of 16.8 mM^−1^ cm^−1^ in a reaction mixture containing 2 mM threo-D,L-isocitrate (Sigma Aldrich), 2 mM MgCl_2_, 10 mM phenylhydrazine HCl (Sigma Aldrich), 2 mM dithiothreitol, and 50 mM potassium phosphate at pH 7.0. Specific activity was determined as the amount of enzyme required to form 1 μmol of glyoxylate- phenylhydrazone per min per mg of total protein. Statistical comparisons were performed using the Student's *t*-test and *p*-values ≤ 0.05 were considered statistically significant.

### *Paracoccidioides* spp. cell dry weight assay

Yeast cells of *P*aracoccidioides spp. were grown as described above. A total of 10^6^ cells/50 mL was inoculated into chemically defined McVeigh/Morton medium (Restrepo and Jiménez, 1980) containing the carbon sources glucose or sodium acetate followed by incubation at 36°C. At each time-point, 10 mL aliquots of the culture were centrifuged at 1,500 × g and the supernatants were carefully removed. The cells were resuspended in 1X PBS up to 500 μL and incubated at 95°C for 1 h. The cells were centrifuged, frozen in liquid nitrogen, and lyophilized for 24 h (Lima et al., 2014). Data are expressed as the mean ± standard deviation of the triplicate independent experiments. Statistical comparisons were performed using Student's *t-*test, and a *p*-value ≤ 0.05 was considered significant.

### Obtaining protein extracts

To obtain the cytoplasmic protein extract, the cells were centrifuged at 10,000 × *g* for 5 min, followed by washing with 1X PBS twice and the addition of Tris-Ca buffer (20 mM Tris–HCl pH 8.8; 2 mM CaCl_2_). This suspension was distributed in tubes containing an equal volume of glass beads (425–600 μm) to the volume of the cell pellet. The cells were disrupted by vigorous mixing in a bead beater apparatus (BioSpec) for 5 cycles for 30 s on ice. The cell lysate was centrifuged at 10,000 × *g* for 15 min at 4°C until no pellet formed. Protein content in the supernatant was quantified using Bradford reagent (Bradford, 1976) using bovine serum albumin as a standard.

### Protein digestion for NanoUPLC-MS^E^ analysis

Proteins were enzymatically digested as described previously (Murad et al., 2011) with some modifications. Briefly, approximately 150 μg of protein (previous item) was added to 10 μL of 50 mM ammonium bicarbonate, pH 8.5, in a microcentrifuge tube. Next, 75 μL of RapiGEST^TM^ SF Surfactante (0.2% v/v) (Waters Corporation, Billerica, MA, USA) was added and the sample was vortexed and incubated in a dry bath at 80°C for 15 min. Disulfide bonds were reduced by adding 2.5 μL of 100 mM dithiothreitol (GE Healthcare, Little Chalfont, UK) at 60°C for 30 min, while cysteines were alkylated by 2.5 μL of 300 mM iodoacetamide (GE Healthcare) for 30 min at room temperature in the dark. The proteins were subsequently digested with 30 μL of trypsin 0.05 μg/μL (Promega, Madison, WI, USA) at 37°C in dry bath for 16 h. To precipitate the RapiGEST, samples were acidified with 30 μL of a 5% (v/v) trifluoracetic acid solution (Sigma-Aldrich) and the mixture was incubated for 90 min at 37°C in a dry bath, followed by centrifugation at 18,000 × *g* at 4°C for 30 min. The supernatants were dried in a speed vacuum (Eppendorf, Hamburg, Germany). All obtained peptides were suspended in 80 μL of solution containing 20 mM of ammonium formate and 80 fmol/μL of PHB (Rabbit Phosphorylase B) (Waters Corporation) (MassPREP^TM^ protein) as an internal standard. Samples were transferred to a Waters Total Recovery vial (Waters Corporation). The samples were then placed in an auto-sampler and stored at 4°C for nano-ultra-high-performance liquid chromatography-mass spectrometry analysis.

### Mass spectrometry

Mass spectrometric experiments were performed on a Synapt G2-S*i* High Definition Mass Spectrometer (Waters Corporation) equipped with mass analyzers such as a hybrid quadrupole/ion mobility mass spectrometry/orthogonal acceleration time-of-flight MS geometry, and coupled to nanoAcquity^TM^ UPLC system (Waters Corporation). The peptide mixture was loaded for 5 min at a flow rate of 8 μL/min for phase A (0.1% formic acid) using a Symmetry C18 trapping column (5 μm particles, 180 μm × 20 mm length; Waters Corporation). The mixture of trapped peptides was subsequently separated by elution over a gradient of 7–35% of phase B (0.1% formic acid in acetonitrile) through a BEH 130 C18 column (1.7 μm particles, 75 × 150 mm; Waters Corporation) in 93 min at 350 nL/min. For each measurement, the mass spectrometer was operated in resolution mode with an *m/z* resolving power of approximately 40,000 full width at half maximum using ion mobility with a cross-section resolving power of at least 40 Ω/ΔΩ. LC–MS multiplex data were collected using ion mobility enhanced MS^E^. Data were acquired in data-independent mode (UDMS^E^) (Distler et al., 2014) over an *m/z* range of 50–2,000 in resolution mode. The exact mass retention time signals from multiplexed ion-mobility DIA scanning (UDMS^E^) were collected in alternating low energy and elevated energy acquisition modes. In the low energy mode, data were collected at 6 eV. In the elevated collision energy, *quasi m/z*-specific collision energies were applied to the different drift time bins to fragment precursor ions prior to orthogonal acceleration time-of-flight analysis, applied to the transfer traveling-wave, collision-induced dissociation cell filled with argon gas with a total cycle time scan of 0.5 s (Souza et al., 2017). Nano electrospray ionization in the positive mode (nanoESI+) source was operated with a capillary voltage of 2.75 kV, block temperature of 70°C, and cone voltage of 30 V. For lock mass correction, [Glu^1^]-Fibrinopeptide B solution (100 fmol/ml in 50% V/V water and acetonitrile, 0.1% V/V formic acid was infused through the reference sprayer at 300 nL/min and sampled every 30 s (Garrido et al., 2016; Abreu et al., 2017). The peptides fractions were analyzed in triplicates.

### Data processing and protein identification

Mass spectrometry raw data of peptide fractions were loaded in Progenesis QI for Proteomics (Nonlinear Dynamics). A reference run for the triplicates was automatically selected as the default. Precursor ion retention times were processed for alignment, peak picking, and normalized to a reference run with default parameters. The MS data were processed by the Apex3D and Peptide3D module using a low-energy threshold of 150 counts, elevated energy threshold of 50 counts, and intensity threshold of 750 counts. Databank searches were performed using Progenesis QIP and the databank of *Paracoccidioides* was used with decoy proteins (http://www.broadinstitute.org/annotation/genome/paracoccidioides_brasiliensis/Multiome.tml). The database was chosen according to the isolated sample set, together with reverse sequences: (i) *Pb*339 (S1) and *Pb*EPM83 (PS3): *P. brasiliensis Pb*18 protein sequences; (ii) *Pb03* (PS2): *P. brasiliensis* (strain *Pb*03) protein sequences; (iii) *P. lutzii* (Pb01) protein sequences.

The mass error tolerance for peptide identification was less than 10 ppm. Protein identification criteria included: (i) the detection of at least 2 fragment ions per peptide, (ii) 5 fragments per protein, (iii) the determination of at least 1 unique (proteotypic) peptide per protein, (iv) carbamidomethylation of cysteine as a fixed modification, (v) phosphorylation of serine, threonine, and tyrosine, and oxidation of methionine were considered as variable modifications, (vi) maximum protein mass (600 kDa), (vii) one missed cleavage site was allowed for trypsin, and (viii) maximum false positive ratio of 4% was allowed. Peptide ion data were exported from Progenesis QIP as ^*^.csv files and edited in Excel (Microsoft®). Only features detected above an intensity threshold of 150 and in at least 2 of 3 replicates were considered for further analysis. To compare the ratios between the control (glucose) and test (sodium acetate), proteins displaying at least 50% differences in expression values compared to the control were considered as regulated. The Uniprot (http://www.uniprot.org) and Pedant on MIPS () database were used for functional classification. The NCBI database was employed to annotate uncharacterized proteins (https://www.ncbi.nlm.nih.gov/).

### Data treatment

Quantitative data on the expression of each protein was converted to a logarithmic scale (log_2_) for analysis purposes. For each protein, the significance of the effects associated with isolates, carbon sources, and interaction between these two factors was evaluated by analysis of variance (ANOVA) using the factorial model, followed by the *Tukey* test. All comparisons were performed using the critical significance level of 0.05. All analyses were performed in R (R Core Team, 2017) software.

The Multi Experiment Viewer software V.4.8 (http://www.tm4.org) was used to group comparative proteomic data. To analyze the isolates, the up- and down-regulated proteins were combined and the expression levels estimated for each protein were used to build a heat map. Differences in proteomic profiles were examined by hierarchical clustering using Pearson correlation as a measure of similarity.

### Ethanol quantification assay

Ethanol concentration was determined using an enzymatic detection kit according to the manufacturer's instructions (UV-test for ethanol, RBiopharm, Darmstadt, Germany). Briefly, ethanol was oxidized to acetaldehyde by the enzyme alcohol dehydrogenase in the presence of nicotinamide-adenine dinucleotide (NAD). Acetaldehyde was quantitatively oxidized to acetic acid in the presence of aldehyde dehydrogenase, releasing NADH, which was determined by measuring the absorbance at 340 nm. *Paracoccidioides Pb*01, *Pb*03, *Pb*339, and *Pb*EPM83 yeast cells were cultivated in minimal media containing acetate (100 mM) or glucose (100 mM), and 10^6^ cells were assayed. Briefly, the cells were centrifuged and lysed using glass beads and a bead beater apparatus (BioSpec) in 5 cycles of 30 s while keeping the samples on ice. The cell lysate was centrifuged at 10,000 × *g* for15 min at 4°C and the supernatant was used for enzymatic assays according to the manufacturer's instructions. The concentrations of ethanol were determined in triplicate. Statistical analysis was performed using Student's *t-*test, and *p* ≤ 0.05 was considered significant.

### Analysis of glucans by fluorescence microscopy

To evaluate the beta glucan content in the cell wall, staining was performed with aniline blue solution 100% (v/v) (Sigma, catalog no. B8563). Yeast cells of different isolates, cultured in the presence of acetate or glucose, were incubated with aniline blue for 5 min while stirring and subsequently washed twice with 1X PBS (Renshaw et al., 2016; De Curcio et al., 2017). Samples stained with aniline blue were visualized under a fluorescence microscope (Zeiss Axiocam MRc-Scope A1, Oberkochen, Germany). A minimum of 100 cells on each microscope slide were used to evaluate fluorescence intensity in triplicate. The software determined the fluorescence intensity (in pixels) and standard error of each analysis. Statistical comparisons were performed using the Student's *t*-test and *p* ≤ 0.05 was considered statistically significant.

### Macrophage infection assays

The survival of the *Paracoccidioides* isolates was determined by quantifying the number of colony-forming units (CFUs) recovered from macrophage infection. J774 1.6 macrophages (Rio de Janeiro Cell Bank–BCRJ/UFRJ, accession number 0273 were employed). Macrophages were maintained in RPMI medium (RPMI 1640, Vitrocell, Brazil) with 10% FBS ([v/v]) and MEM non-essential amino acid solution (Sigma Aldrich), at 36°C and 5% CO_2_ until the cells were completely confluent. The phagocytosis assay was performed in 12-well polypropylene plates (Greiner Bio-One, Kremsmünster, Austria). A total of 10^6^ J774 macrophages were plated per well in RPMI medium containing IFN-γ (1 U/mL) (Sigma Aldrich) and incubated for 24 h at 36°C and 5% CO_2_ for adherence and activation. Prior to co-cultivation, *Paracoccidioides* yeast cells were grown in BHI liquid medium (4% [w/v] glucose, 3.7% [w/v] brain heart infusion, pH 7.2) for 48 h. Next, 5 × 10^6^ yeast cells per well were added to the macrophages, giving a yeast:macrophage cell ratio of 5:1. The cells were incubated for 24 h at 36°C and 5% CO_2_ in RPMI medium containing IFN-γ (1 U/mL). Non-phagocytosed/non-adhered yeast were washed 3 times with PBS (Parente-Rocha et al., 2015). Macrophages were lysed with water and fungal cells were recovered. The number of viable cells was determined based on the number of CFUs. The lysates were plated in BHI medium supplemented with 5% FBS [v/v]. CFUs were determined after growth at 36°C in 5% CO_2_ for 10 days. Data were expressed as the mean value deviation from triplicate measurements and the statistical analyses were performed using ANOVA.

## Results and discussion

### ICL activity

**Figure 1:**
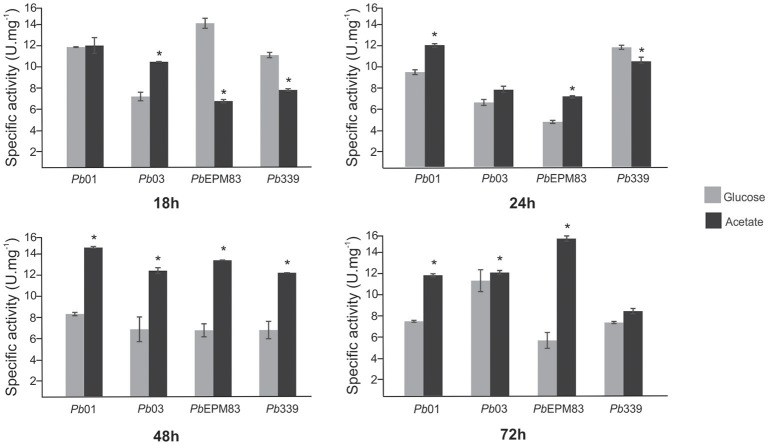
Isocitrate lyase (ICL) activity assay. Activity was determined by measuring the formation of glyoxylate as its phenylhydrazone derivative under each condition. A total of 50 mg of each total protein extract of *Paracoccidioides* isolates *Pb*01, *Pb*03, *Pb*339, and *Pb*EPM83 yeast cells were cultivated in minimal media with sodium acetate (100 mM) and glucose (100 mM) for different times (18, 24, 48, and 72 h). The specific activity of ICL was determined as the amount of enzyme required to form 1 μmol of glyoxylate-phenylhydrazone per minute, per mg of total protein, and represented as U·mg^−1^. Errors bars represent standard deviation from three biological replicates while ^*^ represents *p* ≤ 0.05.

To determine the adequate time of exposure of the isolates to sodium acetate for the proteomic assays, the enzymatic activity of ICL was determined in the four isolates at different time intervals (18, 24, 48, and 72 h). Increased ICL activity was observed in all four isolates at 48 h, indicating activation of the glyoxylic/glyoxylate cycle; thus, this time was used for proteomic analysis (Figure 1). ICL is an enzyme exclusively involved in the glyoxylic/glyoxylate cycle, an anaplerotic carbon pathway that deviates in the decarboxylation steps of the TCA cycle, to produce malate and succinate using acetyl-CoA when the production of pyruvate from glycolysis is reduced (Lorenz and Fink, 2002; Fleck et al., 2011). Studies showed that ICL is an indicator of the glyoxylic/glyoxylate cycle because it allows the formation of glyoxylate from the cleavage of isocitrate, which becomes the substrate for malate synthase. ICL and other enzymes of the glyoxylate cycle are induced under conditions such as low glucose levels and low oxygen tension (Wayne and Lin, 1982; Fernandez et al., 1993) in the presence of carbon sources such as acetate and ethanol and at high temperatures (Bowyer et al., 1994). In *P. brasiliensis, Pb*01 carbon starvation promoted up-regulation of ICL expression, indicating up-regulation of the glyoxylic/glyoxylate cycle in yeast cells after 48 h of treatment (Lima et al., 2014). The enzymatic activity of ICL was reduced when glucose was used as a carbon source in relation to acetate in yeast cells of *P. brasiliensis Pb*01 (Cruz et al., 2011) and was increased under carbon starvation conditions for 48 h (Lima et al., 2014). ICL activity can also be used as an indicator of the glyoxylic/glyoxylate cycle.

### Growth of *P. lutzii* and isolates of *P. brasiliensis* in the presence of glucose and sodium acetate as carbon sources

Yeast cells of *P. lutzii* (*Pb*01) and of *P*. *brasiliensis Pb*03, *Pb*339, and *Pb*EPM83 were grown in the presence of glucose and sodium acetate. Growth was evaluated by dry weight analysis at several time intervals. For all isolates, growth was significantly reduced in the presence of sodium acetate compared to glucose, as depicted in Supplementary Figure 1. The lower growth in acetate likely reflects the lower content of proteins extracted under this condition, as follows: *Pb*01 glucose (14.6 μg/μL), *Pb*01 acetate (10.76 μg/μL); *Pb*03 glucose (10.22 μg/μL), *Pb*03 acetate (8.82 μg/μL); *Pb*339 glucose (8.6 μg/μL), *Pb*339 acetate (7.04 μg/μL); *Pb*EPM83 glucose (11.14 μg/μL), and *Pb*EPM83 acetate (10.78 μg/μL). Similar results were observed in *Escherichia coli* grown on acetate or glucose (Treitz et al., 2016).

### Proteome of *P. lutzii* and of *P. brasiliensis* isolates in the presence of sodium acetate

We identified differentially expressed proteins among *P. lutzii* (*Pb*01) and three members of the species *P. brasiliensis*, as following: *Pb*03, *Pb*339, and *Pb*EPM83. The proteins identified in the four analyzed groups of the genus *Paracoccidioides* are depicted in Supplementary Table 1. A total of 1,160, 1,211, 1,280, and 1,462 proteins were identified in *P. lutzii* (*Pb*01), *Pb*03, *Pb*339, and *Pb*EPM83, respectively, after filtering using the criterion of minimum repeat rate of 2 and a total of 5112 proteins (Supplementary Table 1).

The proteins in each isolate that grew in the presence of acetate were compared to the counterparts in yeast cells growing in the presence of glucose. Statistical analysis revealed differential protein expression in each isolate in relation to the carbon source used. Based on the differential protein expression results, the different isolates were compared using the heat map. There were 526, 442, 744, and 569 differentially expressed proteins showing fold-changes of at least 1.5, for *P. lutzii* (*Pb*01) and *Pb*03, *Pb*339, and *Pb*EPM83, respectively, as depicted in Supplementary Table 1. The number of differentially expressed proteins when comparing the four isolates to the others were 45.3, 36.5, 58.1, and 38.9%, respectively of the total of proteins identified in each isolate, for *P. lutzii* (*Pb*01) and *Pb*03, *Pb*339, and *Pb*EPM83. These data indicate that high metabolic flexibility exists among members of the *Paracoccidioides* genus. Similar results were observed by Pigosso et al. who found significant differences in the protein expression profiles among the representatives of different phylogenetic clades of *Paracoccidioides Pb*01, *Pb*02, *Pb*339, and *Pb*EPM83 (Pigosso et al., 2013).

### Differentially regulated proteins in analyzed *Paracoccidioides* isolates

Based on statistical analysis, the differentially expressed proteins were classified. Supplementary Tables 2–9 present the proteins of the analyzed isolates significantly up- or down-regulated upon growth with the two-carbon molecule acetate. Those proteins were classified according to the functional categories present in FunCat2. The functional categories for up- and down-regulated proteins were similar among the four analyzed isolates, as depicted in Supplementary Figures 2A,B, respectively. Some proteins were assigned as unclassified because of their unknown function, accounting for 20–30% of the analyzed proteins in the isolates (data not shown).

The metabolic pathways, glycolysis/gluconeogenesis and ethanol production, cell wall metabolism, glyoxylic/glyoxylate shunt and the TCA cycle, beta-oxidation and the methylcitrate cycle, electron transport chain and oxidative phosphorylation, amino acids degradation, and response to oxidative stress were analyzed among isolates, as depicted in Supplementary Table 10.

### Regulation of glycolysis/gluconeogenesis and ethanol production

**Figure 2:**
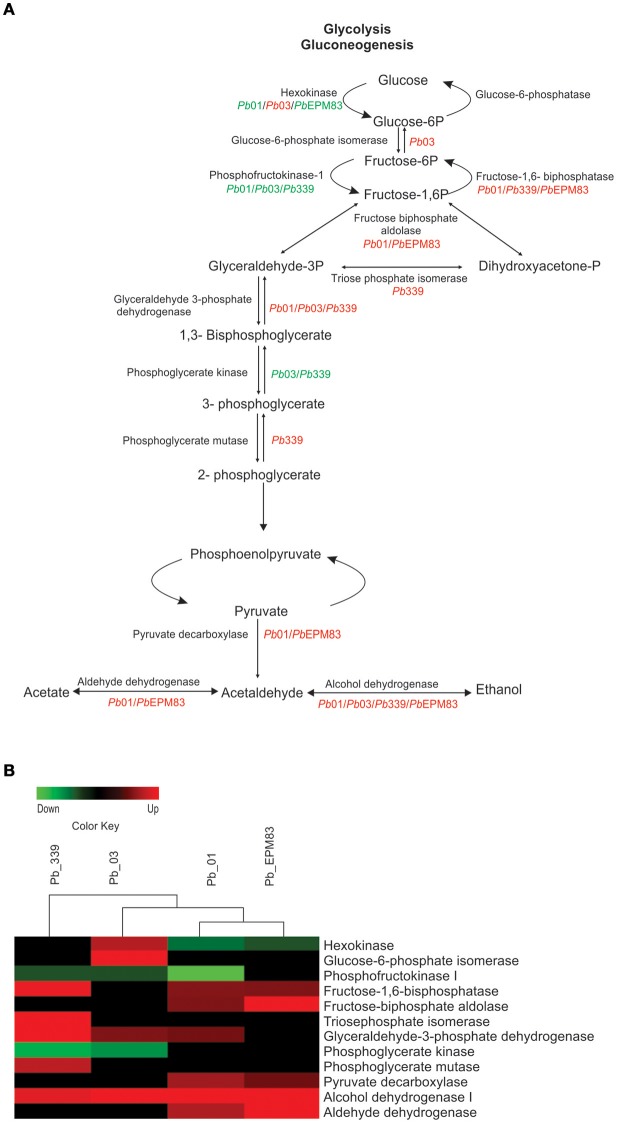
Comparison of protein profiles related to glycolysis and gluconeogenesis in *P. lutzii* and isolates of *P. brasiliensis*. ANOVA was applied to compare expression values among isolates, applying a cut-off of 1.5-fold. Expression data were obtained using the Multi Experiment Viewer software V.4.8, which was used to group and compare expression data. **(A)** Representative diagram of the glycolysis/gluconeogenesis and fermentation pathways depicting down-regulated (green) and up-regulated (red) proteins in the isolates, as cited above. **(B)** Changes in expression levels in yeast cells incubated with acetate in the four analyzed isolates are represented in a heat map format. Mean values of experimental triplicates are shown for down-regulation (green) and up-regulation (red) of genes of isolates *Pb*01, *Pb*03, *Pb*339, and *Pb*EPM83 in the presence of sodium acetate. Black indicates that no significant difference was observed.

To assess changes in the expression of proteins related to glycolysis/gluconeogenesis in the isolates, we compared the levels of enzymes listed in Supplementary Tables 2–9. As depicted in Figures 2A,B, fructose 1,6-biphosphatase (PAAG_02682; PADG_01706) was significantly induced in *Pb*01, *Pb*339, and *Pb*EPM83, suggesting increased gluconeogenesis. Regulatory enzymes of glycolysis, phosphofructokinase-I (PAAG_01583; PABG_03640; PADG_00192) and hexokinase (PADG_03813), were repressed in *Pb*01, *Pb*03, *Pb*339, and *Pb*EPM83. The reciprocal regulation of phosphofructokinase-I and fructose 1,6-biphosphatase clearly indicate increased gluconeogenesis and decreased glycolysis in the isolates of *P. lutzii* and *P. brasiliensis*, as depicted in Figures 2A,B. A shift to gluconeogenesis has been described as a metabolic hallmark of fungal cells exposed to two-carbon sources. Studies of the response of *C. albicans* to internalization by macrophages revealed reprogramming of transcription, including gluconeogenic growth (Lorenz et al., 2004).

**Figure 3:**
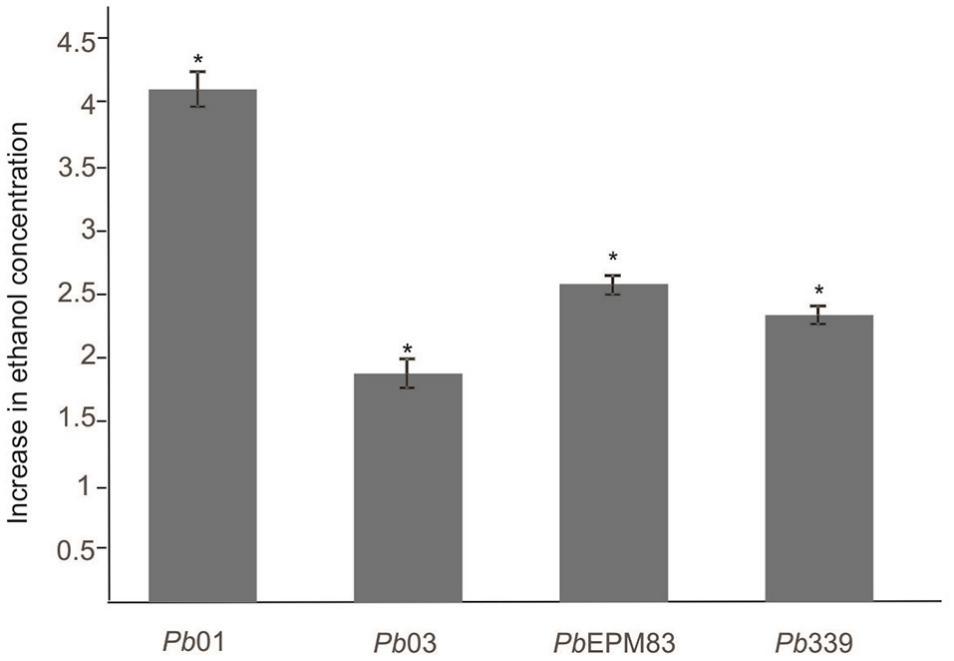
Ethanol measurement in *P. lutzii* and *P. brasiliensis*. The concentration of ethanol (g/L) was determined in yeast cells of the isolates *Pb*01, *Pb*03, *Pb*339, and *Pb*EPM83 upon growth in minimal media with sodium acetate (100 mM) and glucose (100 mM) for 48 h. A total of 10^6^ cells was used for each sample, and the ethanol compound was quantified using an enzymatic detection kit (UV-test for ethanol, RBiopharm, Darmstadt, Germany). Data are expressed as the mean ± standard deviation of biological triplicates in independent experiments. The data were analyzed by two-way ANOVA and *Tukey's* multiple comparisons test. ^*^Indicate significant differences among isolates at *p*-value of ≤0.05.

Three enzymes involved in ethanol production were differentially expressed in the presence of acetate. Pyruvate decarboxylase (PAAG_02050; PADG_00714) and aldehyde dehydrogenase (PAAG_03910) were induced in *Pb*01 and *Pb*EPM83 and alcohol dehydrogenase (PAAG_00403; PABG_04316; PADG_11405) was induced in all studied isolates. Acetate may provide ethanol because of the increase in aldehyde dehydrogenase and alcohol dehydrogenase. A high level of enzymes involved in ethanol production was observed, particularly for *Pb*01 and *Pb*EPM83, which presumably produces ethanol from pyruvate, acetate, and acetaldehyde, as depicted in Figures 2A,B. Ethanol measurement revealed that up to 48 h in presence of sodium acetate, a significantly higher level of ethanol was produced compared to in cells cultured in glucose in all isolates, confirming the proteomic analysis data (Figure 3). In *P. lutzii* during carbon starvation, enzymes related to the production of ethanol were induced, such as alcohol dehydrogenase and pyruvate decarboxylase and ethanol production was increased in cells cultured with glucose (Lima et al., 2014). Comparative proteomic studies among isolates *Pb*01*, Pb*2 *Pb*339, and *Pb*EPM83 in nutrient-rich media revealed a high level of ethanol production in *Pb*01 compared to in the other isolates, suggesting differential production of ethanol among isolates (Pigosso et al., 2013). Ethanol production has been related to fungi virulence. The influence of alcohol dehydrogenase in fungal pathogenesis was observed in invasive pulmonary aspergillosis with an increase in the inflammatory response in the lung of mice infected with an alcohol dehydrogenase null mutant strain, and reduction in fungal burden (Grahl et al., 2011).

### Regulation of cell wall metabolism

**Figure 4:**
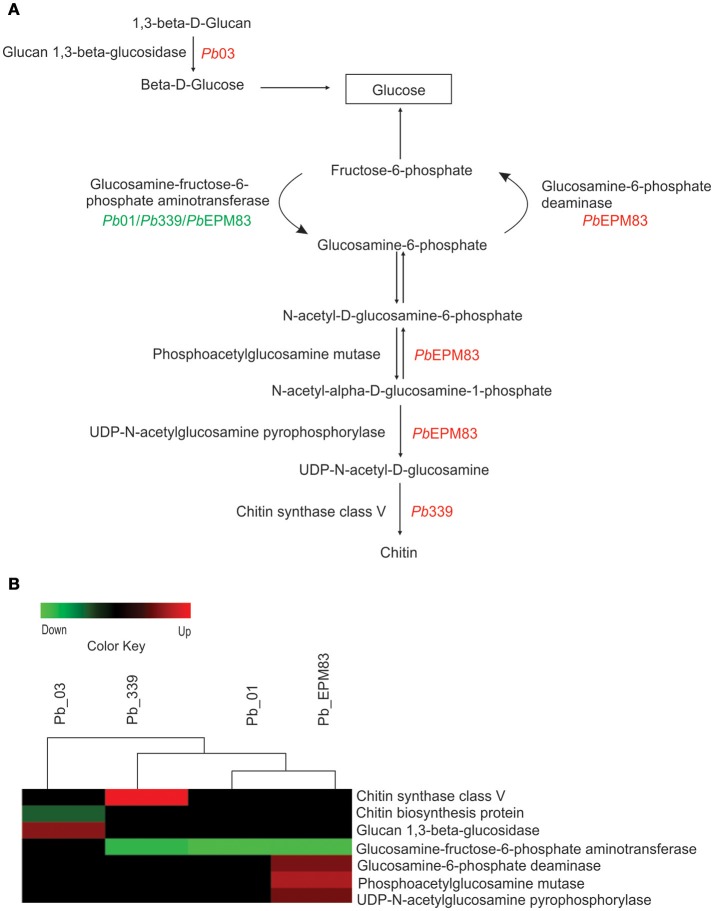
Comparison of protein profiles related to cell wall metabolism in *P. lutzii* and isolates of *P. brasiliensis*. Dataset comparisons were carried out as cited in the Figure 2 legend. **(A)** Representative diagram of the cell wall metabolism pathways depicting down-regulated (green) and up-regulated (red) proteins in the isolates, as cited above. **(B)** Changes in expression levels upon yeast cells incubated with acetate in *Pb*01, *Pb*03, *Pb*339, and *Pb*EPM83 are represented in a heat map format. Black indicates that no significant difference was observed.

Figure 4 depicts differences in the expression of cell wall metabolic enzymes in the presence of acetate in the analyzed isolates. Only *Pb*03 showed up-regulated glucan 1,3-beta-glucosidase, which catalyzes successive hydrolysis of the beta-D-glucose units from the non-reducing ends of (1-3)-beta-D-glucans, releasing beta glucose. Aniline blue, which selectively stains 1,3-beta-D-glucan, was employed to estimate the amount of this polymer in the cell wall, as depicted in Supplementary Figure 3. As depicted in Supplementary Figure 3A, fluorescence was visibly reduced in *Pb*03 grown with acetate. Quantitative analyses of the fluorescence intensity (in pixels) in yeast cells of isolate *Pb*03 in the presence of sodium acetate showed a significant decrease in fluorescence in cells (*p* ≤ 0.05), Supplementary Figure 3B, strongly suggesting a decrease in the glucan content, as indicated by proteomic analysis. Glucosamine-fructose-6-phosphate aminotransferase (PAAG_00850; PADG_03984) was down-regulated in *Pb*EPM83, *Pb*01, and *Pb*339. This enzyme catalyzes the formation of glucosamine 6-phosphate and is the first and rate-limiting enzyme in the hexosamine biosynthetic pathway controlling the flux of glucose into the hexosamine pathway. The final product of the hexosamine pathway, UDP-N-acetyl-glucosamine, is an active precursor of numerous macromolecules containing amino sugars, including chitin in fungi and arthropods (Badet et al., 1987). In our analysis, only *Pb*EPM83 showed up-regulation of the enzymes glucosamine-6-phosphate-deaminase (PADG_00401), phosphoacetylglucosamine mutase (PADG_00604), and UDP-*N*-acetylglucosamine pyrophosphorylase (PADG_04312). These enzymes participate in amino sugar metabolism and cell wall biosynthesis. Phosphoacetylglucosamine mutase reversibly converts *N*-acetyl-alpha-D-glucosamine-1-phosphate to *N*-acetyl-D-glucosamine-6-phosphate. The products from this reaction are substrates to the enzymes glucosamine-6-phosphate-deaminase and UDP-*N*-acetylglucosamine pyrophosphorylase, respectively. The first converts glucosamine-6-phosphate and H_2_O to NH_3_ and fructose-6-phosphate, which is used in glycolysis and gluconeogenesis; the second converts *N*-acetyl-alpha-D-glucosamine-1-phosphate to UDP-*N*-acetyl-D-glucosamine, which is used to produce chitin for the cell wall. Down-regulation of glucosamine: fructose-6-phosphate-aminotransferase (PADG_03984) and up-regulation of glucosamine-6-phosphate-deaminase (PADG_03984) suggests that *Pb*EPM83 mainly uses glucosamine-6-phosphate for glucose production. These results suggest that *Pb*EPM83 can use polymeric carbohydrates precursors to obtain glucose by gluconeogenesis and synthesize chitin polymer into the cell wall.

### Regulation of the glyoxylic/glyoxylate shunt and TCA cycle

**Figure 5:**
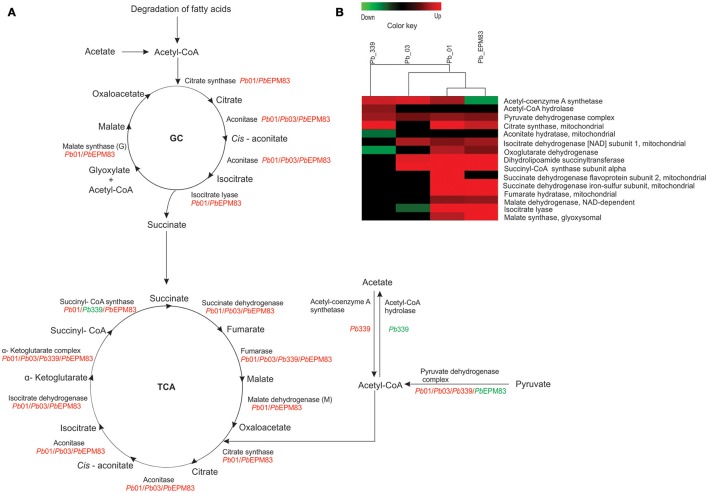
Comparison of protein profiles related to TCA and glyoxylate cycle in *P. lutzii* and isolates of *P. brasiliensis*. Dataset comparisons were carried out as cited in Figure 2 legend. **(A)** Representative diagram of the TCA cycle and glyoxylate cycle depicting down-regulated (green) and up-regulated (red) proteins in the isolates. **(B)** Changes in expression levels upon yeast cells incubated with acetate in *Pb*01, *Pb*03, *Pb*339, and *Pb*EPM83 are represented in a heat map format. Black indicates that no significant difference was observed.

Acetyl-CoA synthase (PADG_01677) and pyruvate dehydrogenase (PAAG_11035; PABG_03494; PADG_07213) can promote the synthesis of acetyl-CoA from acetate and pyruvate, respectively, as depicted in Figures 5A,B. The major fate of acetyl-CoA is the TCA cycle, in which most of enzymes were induced in the analyzed isolates, mainly in *Pb*01 and *Pb*EPM83 (Figures 5A,B). The glyoxylate shunt, which is a shortcut for the decarboxylation steps of the TCA cycle, can allow the use of acetyl-CoA in the synthesis of cellular components (Chung et al., 1988). ICL (PAAG_06951; PADG_01483) and malate synthase (PAAG_04542; PADG_04702) were increased in isolates *Pb*01 and *Pb*EPM83. This aspect is of relevance, as ICL and malate synthase allow the use of two-carbon compounds from the glyoxylic/glyoxylate cycle. Induction of transcripts encoding ICL and malate synthase was observed in phagocytized *C. albicans* upon macrophage infection, enhancing the importance of acquiring non-preferential carbon sources and glyoxylate cycle function in glucose synthesis (Lorenz and Fink, 2001). Thus, activation of this metabolic pathway enables the pathogen to survive and persist in the host in tissues with limited nutrients such as inside macrophages. As described above, the glyoxylic/glyoxylate cycle is induced in fungi and bacteria during phagocytosis as a response to nutrient deprivation in the phagolysosome (Brock, 2009). Genes encoding the enzymes of the glyoxylate cycle, ICL and malate synthase, were induced in *C. albicans* upon phagocytosis by murine macrophages (Lorenz et al., 2004) and human neutrophils (Fradin et al., 2015). This metabolic pathway enables the fungus to utilize two-carbon molecules as a carbon source, and the induction of this pathway likely reflects the nutrient-deprived environment inside phagocytes (Miramón et al., 2012). The role of the glyoxylate cycle in virulence has been described in organisms, such as *Aspergillus fumigatus* (Olivas et al., 2007), *C. albicans* (Lorenz and Fink, 2001, 2002), and *Mycobacterium tuberculosis* (McKinney et al., 2000; Munõz-Elias and McKinney, 2005).

Regarding the genus *Paracoccidioides*, the induction of ICL was observed in *Pb*01 during the transition from mycelium to yeast cells (Bastos et al., 2007) and in yeast cells recovered from the kidneys of infected mice (Costa et al., 2007). The glyoxylate cycle was positively regulated in yeast cells of *P. lutzii* (Felipe et al., 2005) and the level of malate synthase transcript was higher in yeast cells in the presence of a two-carbon source in relation to glucose (Zambuzzi-Carvalho et al., 2009). Increased expression of ICL was also observed in *Pb*01 from 6 to 12 h under carbon starvation (Lima et al., 2014).

### Regulation of β-oxidation and methyl citrate cycle

**Figure 6:**
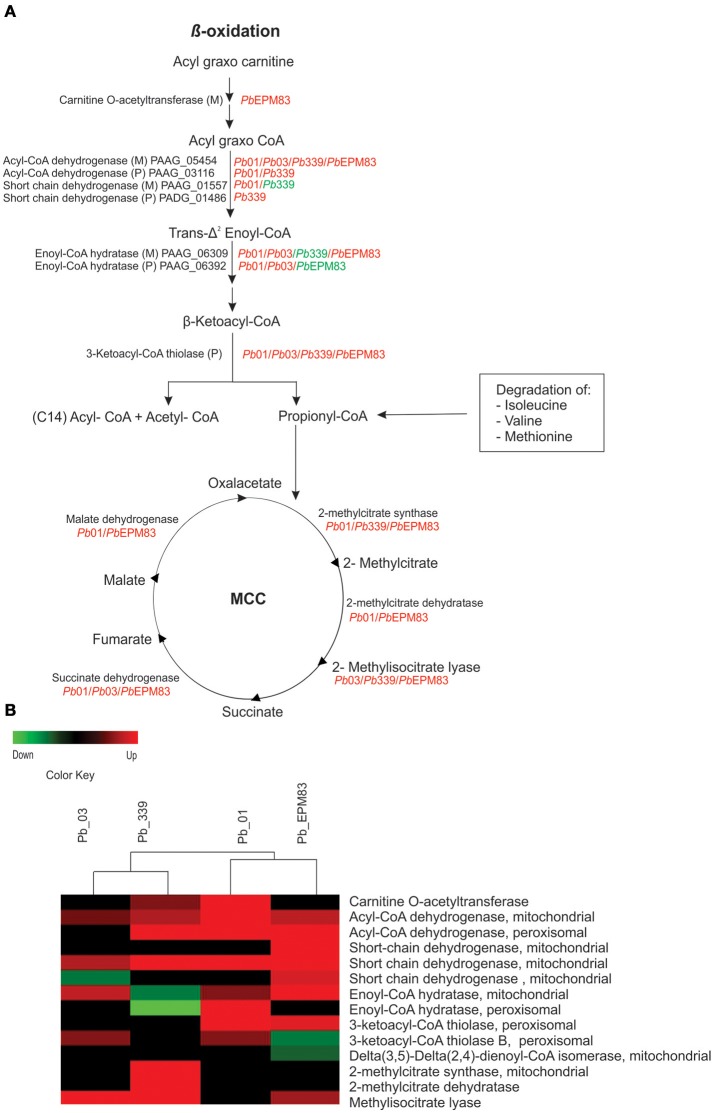
Comparison of protein profiles related to β-oxidation and methyl citrate cycle in *P. lutzii* and isolates of *P. brasiliensis*. Dataset comparisons were carried out as cited in Figure 2, legend. **(A)** Representative diagram of the β-oxidation and methyl citrate cycle pathways depicting down-regulated (green) and up-regulated (red) proteins in the isolates **(B)** Changes in expression levels in yeast cells incubated with acetate in *Pb*01, *Pb*03, *Pb*339, and *Pb*EPM83 are represented in a heat map format. Black indicates that no significant difference was observed.

In general, the beta-oxidation of fatty acids mainly functions in *Pb*01 and *Pb*EPM83 (PAAG_06329; PAAG_02664; PAAG_05454; PAAG_03116; PAAG_06309; PAAG_06392; PAAG_01557; PADG_01228; PADG_01687; PADG_06805; PADG_07023; PADG_06721; PADG_01209; PADG_02527), producing acetyl-CoA and propionyl-CoA. Acetyl-CoA can be consumed in the glyoxylate cycle for biosynthetic purposes or in the TCA cycle to generate cellular energy as reduced cofactors. Beta-oxidation was induced in the mitochondria and peroxisomes, as shown in Figure 6, with the up-regulation of enzymes in both compartments. Carnitine *O*-acetyl transferase (PADG_07023), which is related to the entry of acyl fatty acid into the mitochondria, and acylCoA dehydrogenase mitochondrial (PAAG_05454; PABG_01791; PADG_06805), which catalyzes the initial step in each cycle of fatty acid β-oxidation, were up-regulated in *Pb*01, *Pb*03, *Pb*339, and *Pb*EPM83, suggesting higher fatty acid degradation in those isolates in the presence of acetate.

Enzymes levels in the methyl citrate cycle were induced or maintained, as shown in Figure 6, indicating that this cycle was active in the presence of acetate. Notably, all identified enzymes in the cycle were increased in *Pb*339 (PADG_04709; PADG_04710; PADG_04718; PADG_05281). Methyl citrate synthase is a key enzyme in the methyl citrate cycle and is essential for the degradation of propionyl-CoA in fungi and some bacteria, avoiding accumulation of propionyl, which is toxic (Ibrahim-Granet et al., 2008). The accumulation of propionyl in *Aspergillus nidulans* and *A. fumigatus* inhibits growth and secondary metabolism, and occurs when there are defects in the enzyme methyl citrate synthase (Brock and Buckel, 2004; Zhang et al., 2004; Maerker et al., 2005). Methyl citrate synthase is essential in *A. fumigatus* for the manifestation of the invasive properties of aspergillosis, suggesting that amino acids serve as nutritional support for the growth and invasion of *A. fumigatus* (Ibrahim-Granet et al., 2008).

### Electron transport chain and oxidative phosphorylation

Additionally, in *Pb*EPM83, the electron transport chain and ATP synthase complex were predominantly induced but no classes of proteins were repressed, as depicted in Supplementary Figure 4. Proteins of the different respiratory chain complexes were differentially regulated among isolates (Supplementary Figure 4). *Pb*EMP83 showed the highest number of induced respiratory chain proteins/enzymes, whereas in *Pb*01, most components of the electron transport system and membrane-associated energy conservation were not induced, corroborating the results of Pigosso and colleagues who demonstrated that *P. lutzii* (*Pb*01) is metabolically more anaerobic than *P. brasiliensis* (Pigosso et al., 2013).

### Regulation of amino acids degradation

**Figure 7:**
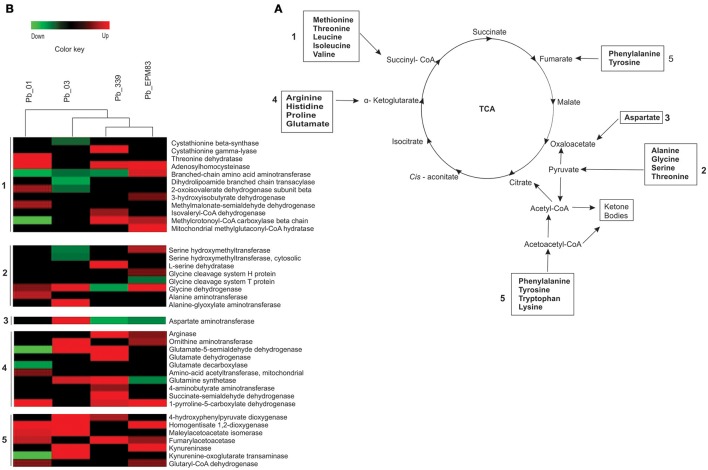
Comparison of protein profiles related to amino acid catabolism in *P. lutzii* and isolates of *P. brasiliensis*. Dataset comparisons were carried out as cited in Figure 2 legend. **(A)** Changes in expression levels in yeast cells incubated with acetate in *Pb*01, *Pb*03, *Pb*339, and *Pb*EPM83 are represented in a heat map format. **(B)** Representative diagram of the amino acid catabolism pathways depicting down-regulated (green) and up-regulated (red) proteins in the isolates, as cited above. Black indicates that no significant difference was observed.

The degradation of amino acids providing precursors for the TCA cycle, pyruvate, or acetyl-CoA was predominantly induced in the analyzed isolates (Figure 7). Higher production of succinyl-CoA appeared to occur in *Pb*01 from the degradation of methionine and threonine because of the induction of adenosyl homocysteinase (PAAG_02859) and threonine dehydratase (PAAG_03168) (Figure 7 and Supplementary Figure 5). Glyoxylate production from the degradation of glycine was observed in *Pb*01, *Pb*03, and *Pb*EPM83 with the induction of the enzyme glycine dehydrogenase (PAAG_1568; PABG_04990) (Figure 7 and Supplementary Figure 5). *Pb*01 also induced alanine aminotransferase (PAAG_08207), producing pyruvate from alanine. Notably, *Pb*03 appeared to induce the interconversion of alanine and glyoxylate to pyruvate because of the up-regulation of alanine glyoxylate aminotransferase (PABG_00589), allowing pyruvate synthesis from the transamination of alanine and glyoxylate synthesized by the glyoxylate cycle (Figure 7 and Supplementary Figure 5). *Pb*03 may be the most efficient producer of oxaloacetate from asparagine because aspartate aminotransferase (PABG_02806) was up-regulated in this strain (Figure 7 and Supplementary Figure 5).

The degradation of phenylalanine and tyrosine to acetoacetyl-CoA was expected to be very similar in all isolates because they upregulate at least two enzymes in the pathway (Figure 7; Supplementary Figure 6). The degradation of tryptophan rendering acetyl-CoA may be higher in *Pb*EPM83, and *Pb*03 because of up-regulation of kyneurinase (PADG_00349; PABG_01965) (Figure 7 and Supplementary Figure 6).

The conversion of arginine and ornithine to alpha-ketoglutarate appeared to predominate in *Pb*339, *Pb*03, and *Pb*EPM83, as the enzymes in this pathway such as arginase (PADG_00637) and ornithine aminotransferase (PABG_02827; PADG_01328) were up-regulated in the three isolates (Figure 7 and Supplementary Figure 7).

The isolates *Pb*EPM83 and *Pb*339 may efficiently produce acetyl-CoA from leucine because of the induction of the enzyme methylcrotonyl-CoA carboxylase (PADG_07370) in both isolates, as depicted in Figure 7 and Supplementary Figure 8. Related to this pathway, the enzymes isovaleryl-CoA dehydrogenase (PADG_07369) and methylglutaconyl-CoA hydratase (PADG_00643) were induced in *Pb*339 and *Pb*EPM83, respectively. However, *Pb*01 and *Pb*03 may be less efficient in producing acetoacetate from leucine, while the enzymes in this pathway were not up-regulated and the methylcrotonyl-CoA carboxylase (PAAG_04103) was repressed (Figure 7 and Supplementary Figure 8). The conversion of valine and isoleucine to succinyl-CoA may be more predominant in *Pb*EPM83 and *Pb*01 because of up-regulation of the enzymes acylCoA dehydrogenase (PAAG_05454; PADG_06805) and enoyl-CoA hydratase (PAAG_06309; PADG_01209) in both (Figure 7, Supplementary Figure 8), as well as the up-regulation of 2-oxovalerate dehydrogenase (PAAG_01194) and methylmalonate-semialdehyde dehydrogenase (PAAG_07036) in *Pb*01 and 3-hydroxyisobutyrate dehydrogenase (PADG_03466) and branched-chain amino acid aminotransferase (PADG_04570) in *Pb*EPM83 (Figure 7, Supplementary Figure 8).

The catabolism of amino acids is highly induced in *Paracoccidioides*. In the presence of a non-preferential carbon source, there was a metabolic shift to the catabolism of amino acids to produce TCA intermediates. Similar results were obtained in *E. coli*, in which acetate as the carbon source activates amino acid degradation (Treitz et al., 2016). Upon nutrient deprivation, *C. albicans* utilizes amino acids as the main carbon source, leading to ammonia excretion which increases the environmental pH and contributes to morphogenesis (Vylkova et al., 2011). *Paracoccidioides lutzii*, upon carbon deprivation, activates the degradation of amino acids to provide precursors for gluconeogenesis (Lima et al., 2014). Additionally, upon phagocytosis, *P. brasiliensis* induces the enzymes glutamate dehydrogenase, alanine glyoxylate aminotransferase, and aspartate aminotransferase, indicating the importance of the catabolism of amino acids for obtaining glucose precursors and energy in the harsh environment of the phagolysosome (Parente-Rocha et al., 2015).

### Regulation of stress response

**Figure 8:**
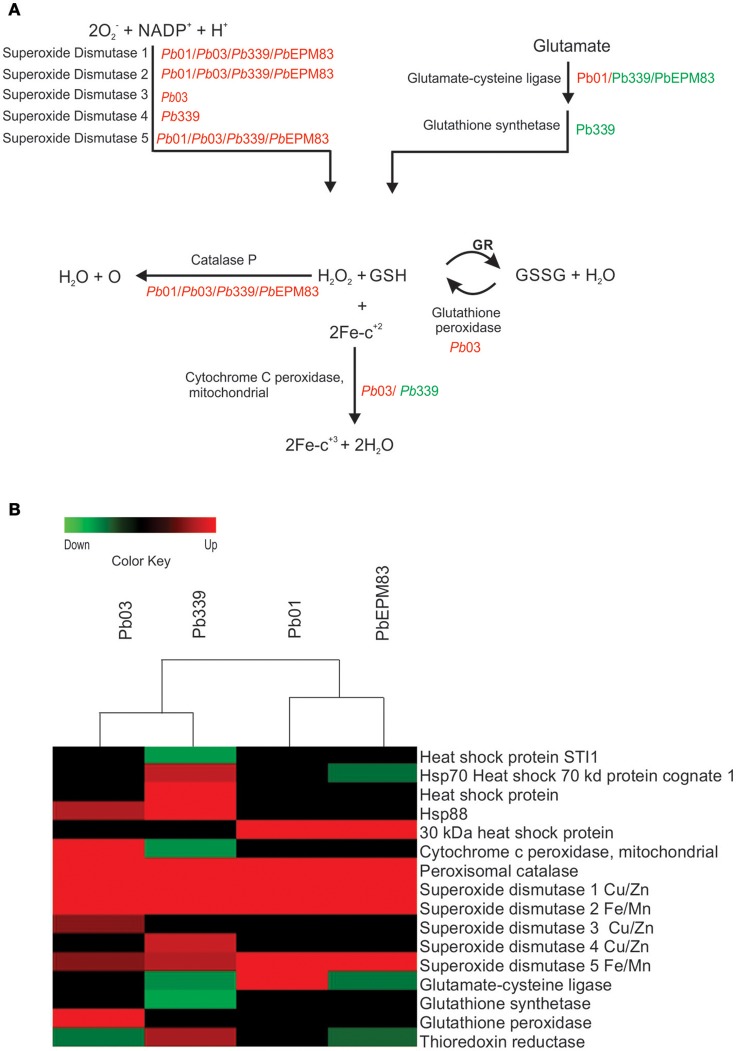
Comparison of protein profiles related to detoxification mechanisms against oxidative stress in *P. lutzii* and isolates of *P. brasiliensis*. Dataset comparisons were carried out as cited in Figure 2 legend. **(A)** Changes in expression levels in yeast cells incubated with acetate in Pb01, *Pb*03, *Pb*339, and *Pb*EPM83 are represented in a heat map format. **(B)** Representative diagram of the detoxification mechanisms against oxidative stress pathways depicting down-regulated (green) and up-regulated (red) proteins in the isolates as cited above. Black indicates that no significant difference was observed. *Glutathione (GSH)*; glutathione reductase (GR); glutathione disulfide (GSSG).

We observed the induction of several proteins and enzymes related to ROS detoxification in pathogenic microorganisms in the different isolates of *P. brasiliensis* and *P. lutzii* when yeast cells were cultured in the presence of sodium acetate, as depicted in Figure 8. Detoxifying molecules are involved in the pathogenesis and virulence of pathogens in phagocytes (Enjalbert et al., 2007; Youseff et al., 2012). In all analyzed isolates, we detected increases in superoxide dismutases (SODs), Cu/Zn-containing SOD1 (PAAG_04164; PABG_03954; PADG_07418), Fe/Mn-containing SOD2 (PAAG_02725; PABG_03204; PADG_01755), SOD5 (PAAG_02926; PABG_03387; PADG_01954), and peroxisomal catalase (PAAG_01454; PADG_00324; PABG_01943). SOD is a primary antioxidant defense against ROS which dismutates the superoxide radical ($O_{2}^{.-}$) into molecular oxygen and H_2_O_2_ (Turrens, 2003). It has been previously suggested that one of the mechanisms used by *P. brasiliensis* for eliminating ROS is the expression of proteins from the antioxidant system, such as SOD enzymes, to neutralize superoxide and convert them into less harmful molecules such as hydrogen peroxide and oxygen molecules (Campos et al., 2005; de Arruda Grossklaus et al., 2013). The *Paracoccidioides* genome database lists six SOD isoforms. Analysis of gene expression between phylogenetic lineages of *Paracoccidioides* revealed the induction of *SOD1* and *SOD3* during the transition from mycelium-to-yeast, exposure to oxidative agents, and interaction with phagocytic cells (Tamayo et al., 2016). Catalase and peroxidase catabolize H_2_O_2_. *Paracoccidioides* isolates encode three catalases, *PbCatA, PbCatP*, and *PbCatB*, which catalyze the decomposition of H_2_O_2_ into oxygen and water (Chagas et al., 2008; Tamayo et al., 2017). CATP was up-regulated in all isolates of *Paracoccidioides* (Figure 8). Cytochrome C peroxidase (PABG_00720) and glutathione peroxidase (PABG_04219) were increased only in *Pb*03 compared to the other isolates upon incubation when acetate was the only carbon source. It was previously demonstrated that *Pb*01, *Pb*339, and *Pb*EPM83 are less resistant to incubation with menadione compared to *Pb*02, which belongs to the same group of *Pb*03 evaluated in this study (Pigosso et al., 2013; Muñoz et al., 2016), corroborating our results. In proteomic analysis, during infection of macrophages, *P. brasiliensis* induced the expression of gamma glutamyltranspeptidase, cytochrome c peroxidase, Cu/Zn SOD, and thioredoxins. Cytochrome C peroxidase knockdown mutant strains show reduced survival upon macrophage interactions and during infection in mouse (Parente-Rocha et al., 2015). All isolates analyzed in this study showed similar survival rates upon macrophage phagocytosis (Supplementary Figure 9), suggesting that they possess equivalent mechanisms for avoiding oxidative stress caused by phagocytes.

### Induced processes in *P. lutzii* and *P. brasiliensis* isolates

**Figure 9:**
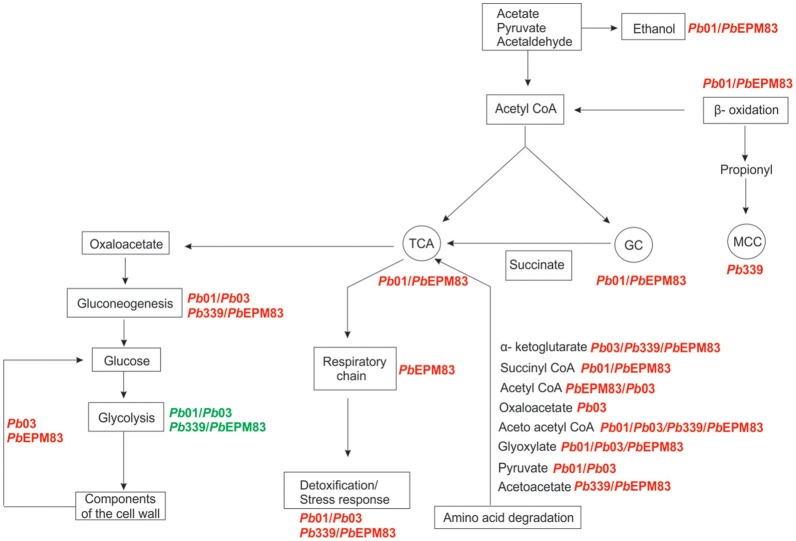
Overview of metabolic features of members of the *Paracoccidioides* genus in sodium acetate as carbon source. The metabolic pathways were identified by analyzing up-regulated proteins in *P. lutzii* (*Pb*01) and *P. brasiliensis Pb*03, *Pb*339, and *Pb*EPM83. The main metabolic pathways were represented: gluconeogenesis, fermentation, cycles of the tricarboxylic acid (TCA), glyoxylate (GC) and methyl citrate (MCC), respiratory chain, β- oxidation, amino acid degradation, detoxification/response to stress, and cell wall components.

By comparing the metabolic pathways that reflect carbon flux distribution, preferential metabolic strategies can be proposed for the analyzed *Paracoccidioides* isolates in the presence of acetate as the only carbon source. Figure 9 depicts the variations of *in vivo* carbon flux distribution of enzymes involved in central metabolism. Gluconeogenesis is thought to occur at similar rates in *P. brasiliensis* and *P. lutzii* because of similar induction of the enzymes in the pathway (Figure 2). *Pb*03 and *Pb*EPM83 are thought to use components or intermediates of cell wall catabolism to produce glucose, as depicted in Figures 4, 9. *Pb*01 and *Pb*EPM83 may increase the glyoxylate cycle and TCA cycle to a greater extent than *Pb*03 and *Pb*339, as depicted in Figures 5, 9. β-Oxidation predominates in *Pb*01 and *Pb*EPM83, as depicted in Figures 5, 9. The methyl citrate cycle predominates in *Pb*339, as shown in Figures 6, 9. Regarding the oxidative phosphorylation and ATP synthesis, *Pb*EPM83 showed greater induction than other isolates of the *Paracoccidioides* genus. The catabolism of amino acids producing intermediates of the TCA cycle was prominent among the isolates. *Pb*03 and *Pb*EPM8 were the most efficient producers of precursors to for the TCA cycle from amino acid metabolism. For the detoxification process, all analyzed isolates in the *Paracoccidioides* genus can detoxify H_2_O_2_ through catalase P. *Pb*03 showed induction of glutathione peroxidase and cytochrome C peroxidase, which may account for the higher potential for eliminating ROS (Figures 8, 9). Taken together, we suggest that *Pb*EPM83 uses aerobic metabolism, including the TCA cycle and glyoxylate shunt, to produce ATP through oxidative phosphorylation.

## Concluding remarks

*Paracoccidioides* respond to the presence of acetate in the growth medium by increasing the expression of systems devoted to utilization of this compound, which requires changes in the abundance of a large number of proteins. Although this general metabolic reprogramming which includes a shift to the glyoxylate shunt induced gluconeogenesis and catabolism of amino acids, it was possible to determine the quantitative and qualitative differences in the metabolism of different *Paracoccidioides* species when acetate was the only carbon source. Whether these differential changes reflect the biological fitness of *Paracoccidioides* within host niches remains unclear.

## Author contributions

CdAS conceived and finalized the manuscript. LB, FdM, and LP performed the experiments. GdS carried out proteomic data. AC carried out statistical analyzes. LB, FdM, LP, MP, and CdAS designed the study, discussed, analyzed, interpreted the data and writing the manuscript.

## Conflict of interest statement

The authors declare that the research was conducted in the absence of any commercial or financial relationships that could be construed as a potential conflict of interest. The reviewer EL-R and handling Editor declared their shared affiliation.
