# Supplementary material for: Differential Metabolism of a Two-Carbon Substrate by Members of the Paracoccidioides Genus
Source: Front Microbiol. 2017 Nov 27;8:2308. doi: 10.3389/fmicb.2017.02308 (PMC5711815; doi:10.3389/fmicb.2017.02308)
Supplement: Supplementary file 1 [file Table1.DOCX]

**Supplementary Table 1: Total proteins identified in** *P. lutzii* **and** *P. brasiliensis* **grown in the presence of 100mM sodium acetate for 48 hours obtained by NanoUPLC-MS^E^ analysis**.

| **PROTEIN / STRAIN** | ***Pb01*** | ***Pb*03** | ***Pb*339** | ***Pb*EPM83** |
| --- | --- | --- | --- | --- |
| **Total of Proteins** | 1160 | 1211 | 1280 | 1462 |
| **Constitutive** | 634 | 769 | 536 | 893 |
| **Regulated** | 526 | 442 | 744 | 569 |
| **Up** | 316 | 230 | 348 | 329 |
| **Down** | 210 | 212 | 396 | 240 |

The proteins were normalized to an internal standard and it was considered as regulated the proteins with fold change of 1.5 obtained from analysis of ProteinLynx Global Server (PLGS) version 3.0 (Waters Corporation. Manchester. UK).
