# Supplementary material for: Differential Metabolism of a Two-Carbon Substrate by Members of the Paracoccidioides Genus
Source: Front Microbiol. 2017 Nov 27;8:2308. doi: 10.3389/fmicb.2017.02308 (PMC5711815; doi:10.3389/fmicb.2017.02308)
Supplement: Supplementary file 2 [file Table2.DOCX]

**Supplemental Table 2: Proteins down-regulated in** *Paracoccidioides lutzii* **after growth for 48 hours in sodium acetate as carbon source.**

| **Accession number^a^** | **Protein Description^b^** | **Acetate/Glucose Ratio^c^** | **Score** |
| --- | --- | --- | --- |
| **Functional categories^d^** | |  |  |
| **1. METABOLISM** | | | |
| **Amino acid metabolism** | | | |
| PAAG_01383 | 3-deoxy-7-phosphoheptulonate synthase | 0.35 | 80.55 |
| PAAG_07605 | Acetolactate synthase, small subunit | 0.46 | 94.26 |
| PAAG_04563 | Amidophosphoribosyltransferase | 0.38 | 68.93 |
| PAAG_04401 | Branched-chain amino acid aminotransferase | 0.29 | 130.10 |
| PAAG_03506 | Glutamate decarboxylase | 0.38 | 100.03 |
| PAAG_00850 | Glutamine-fructose-6-phosphate transaminase | 0.10 | 350.67 |
| PAAG_07089 | Homocitrate synthase, mitochondrial | 0.22 | 148.51 |
| PAAG_01711 | tRNA methyltransferase Trm5 | 0.51 | 107.16 |
| PAAG_02603 | Aspartate aminotransferase | 0.45 | 164.90 |
| PAAG_05743 | Aromatic amino acid aminotransferase | 0.55 | 10.86 |
| PAAG_08900 | 2,2-dialkylglycine decarboxylase | 0.37 | 48.37 |
| PAAG_07566 | Methionine aminopeptidase 2A | 0.55 | 49.76 |
| PAAG_04528 | N-acetyl-gamma-glutamyl-phosphate reductase | 0.39 | 155.54 |
| PAAG_06289 | Tryptophan synthase | 0.26 | 92.44 |
| PAAG_11922 | 3-hydroxyanthranilate 3,4-dioxygenase | 0.43 | 18.54 |
|  |  |  |  |
| **Nitrogen, sulfur and selenium metabolism** | | | |
| PAAG_06237 | Urease accessory protein UreG | 0.19 | 59.82 |
|  |  |  |  |
| **Nucleotide/nucleoside/nucleobase metabolism** | | | |
| PAAG_06906 | Adenine phosphoribosyltransferase | 0.28 | 48.80 |
| PAAG_08019 | Adenylate kinase 1 | 0.66 | 65.27 |
| PAAG_00316 | Guanylate kinase | 0.63 | 67.93 |
| PAAG_06700 | Uridylate kinase | 0.33 | 77.05 |
| PAAG_03466 | 5'-nucleotidase | 0.45 | 32.68 |
| PAAG_00886 | Orotate phosphoribosyltransferase | 0.41 | 10.58 |
| PAAG_05613 | Phosphoribosylglycinamide formyltransferase | 0.34 | 17.28 |
| PAAG_00211 | Pyrimidine 5'-nucleotidase | 0.23 | 15.96 |
| PAAG_07682 | Ribonucleoside-diphosphate reductase large chain | 0.11 | 35.31 |
| PAAG_08597 | Thymidylate synthase | 0.47 | 35.24 |
|  |  |  |  |
| **C-compound and carbohydrate metabolism** | | | |
| PAAG_04888 | 4-coumarate-CoA ligase | 0.30 | 69.57 |
| PAAG_04602 | Mannosyl-oligosaccharide glucosidase | 0.34 | 37.84 |
| PAAG_00889 | Phosphomannomutase | 0.61 | 169.26 |
| PAAG_02382 | Puinone oxidoreductase | 0.27 | 178.56 |
| PAAG_06817 | UTP-glucose-1-phosphate uridylyltransferase | 0.11 | 137.03 |
| PAAG_04985 | Magnesium-dependent phosphatase-1 | 0.30 | 18.42 |
| PAAG_02767 | Formamidopyrimidine-DNA glycosylase | 0.56 | 6.55 |
|  |  |  |  |
| **Lipid, fatty acid and isoprenoid metabolism** | | | |
| PAAG_01524 | Fatty acid synthase subunit beta dehydratase | 0.35 | 821.89 |
| PAAG_03123 | 3-oxoacyl-(acyl-carrier-protein) reductase | 0.20 | 10.60 |
| PAAG_02007 | Oxysterol binding protein | 0.61 | 112.49 |
|  |  |  |  |
| **Metabolism of vitamins, cofactors, and prosthetic groups** | | | |
| PAAG_00673 | NUDIX domain-containing protein | 0.63 | 17.04 |
| PAAG_02418 | Porphobilinogen deaminase | 0.49 | 42.44 |
| PAAG_05032 | Uroporphyrinogen-III synthase | 0.48 | 22.91 |
| PAAG_06513 | Biotin-protein ligase ligase | 0.63 | 49.10 |
| PAAG_03464 | Bleomycin hydrolase | 0.19 | 90.89 |
|  |  |  |  |
| **2. ENERGY** | | | |
| **Glycolysis and gluconeogenesis** | | | |
| PAAG_01583 | 6-phosphofructokinase subunit beta | 0.59 | 220.05 |
| PAAG_01015 | Hexokinase | 0.54 | 140.03 |
| PAAG_02189 | Aldolase | 0.62 | 42.04 |
|  |  |  |  |
| **Pentose-phosphate pathway** | | | |
| PAAG_01178 | 6-phosphogluconate dehydrogenase | 0.62 | 316.09 |
| PAAG_02633 | Ribose-phosphate pyrophosphokinase 3 | 0.51 | 115.37 |
|  |  |  |  |
| **Tricarboxylic-acid pathway** | | | |
| PAAG_07843 | Aconitate hydratase | 0.56 | 62.72 |
|  |  |  |  |
| **Electron transport and membrane-associated energy conservation** | | | |
| PAAG_07586 | Cytochrome b2 | 0.64 | 42.71 |
|  |  |  |  |
| **3. CELL CYCLE and DNA PROCESSING** | | | |
| PAAG_02627 | RuvB-like helicase 1 | 0.43 | 59.34 |
| PAAG_09097 | DNA mismatch repair protein Msh3 | 0.65 | 52.14 |
| PAAG_02773 | Ubiquitin-conjugating enzyme variant MMS2 | 0.44 | 45.34 |
| PAAG_05197 | Protein BCP1 | 0.18 | 41.13 |
| PAAG_01325 | Arf gtpase-activating protein | 0.36 | 28.53 |
| PAAG_03129 | Dynein light chain | 0.32 | 11.29 |
| PAAG_04977 | Ubiquitin-conjugating enzyme | 0.66 | 34.66 |
| PAAG_04846 | COP9 signalosome complex subunit 4 | 0.55 | 17.31 |
| PAAG_12506 | Tubulin alpha-1 chain | 0.17 | 94.72 |
| PAAG_03031 | Tubulin beta chain | 0.43 | 73.92 |
| PAAG_00317 | Septin 4 | 0.45 | 83.35 |
|  |  |  |  |
| **4. TRANSCRIPTION** | | | |
| PAAG_05734 | DNA-directed RNA polymerases I and III | 0.03 | 6.10 |
| PAAG_02055 | Histone chaperone asf1 | 0.52 | 41.84 |
| PAAG_06917 | KH domain RNA-binding protein | 0.54 | 128.35 |
| PAAG_06891 | mRNA binding post-transcriptional regulator | 0.40 | 73.27 |
| PAAG_05121 | Phosducin family protein | 0.64 | 6.73 |
| PAAG_08117 | Transcriptional regulator | 0.42 | 115.88 |
| PAAG_04662 | Cleavage and polyadenylation specificity factor subunit 5 | 0.61 | 134.41 |
| PAAG_00723 | Small nuclear ribonucleoprotein B and B' | 0.66 | 66.05 |
| PAAG_01062 | Small nuclear ribonucleoprotein Sm D1 | 0.41 | 70.77 |
| PAAG_08223 | Small nuclear ribonucleoprotein Sm D3 | 0.65 | 23.75 |
| PAAG_07775 | Heat shock protein SSB1 | 0.61 | 336.29 |
| PAAG_05711 | Splicing factor U2AF 50 kDa subunit | 0.47 | 44.94 |
| PAAG_00847 | Multifunctional methyltransferase subunit | 0.46 | 11.71 |
| PAAG_03941 | G4 quadruplex nucleic acid binding protein | 0.58 | 142.62 |
| PAAG_05172 | Zinc finger protein zpr1 | 0.48 | 76.86 |
| PAAG_11028 | DNA polymerase epsilon subunit C | 0.50 | 12.00 |
|  |  |  |  |
| **5. PROTEIN SYNTHESIS** | | | |
| PAAG_01097 | Poly(rC)-binding protein | 0.51 | 112.89 |
| PAAG_00385 | 40S ribosomal protein S23 | 0.64 | 91.94 |
| PAAG_09083 | TCTP family protein | 0.53 | 94.75 |
| PAAG_00724 | 60S ribosomal protein L11 | 0.63 | 117.35 |
| PAAG_00801 | 60S acidic ribosomal protein P0 | 0.45 | 53.61 |
| PAAG_02578 | Mitochondrial 54S ribosomal protein MRPL51 | 0.64 | 33.18 |
| PAAG_05484 | 40S ribosomal protein S5 | 0.55 | 154.19 |
| PAAG_09043 | 40S ribosomal protein S2 | 0.52 | 176.32 |
| PAAG_08205 | 26S proteasome non-ATPase regulatory subunit 6 | 0.65 | 113.45 |
| PAAG_05943 | 26S proteasome non-ATPase regulatory subunit 12 | 0.62 | 73.96 |
| PAAG_00234 | Translation initiation factor IF-2 | 0.50 | 41.56 |
| PAAG_00689 | ATP-dependent RNA helicase eIF4A | 0.44 | 179.97 |
| PAAG_01330 | Eukaryotic translation initiation factor 3 | 0.28 | 29.64 |
| PAAG_01425 | Eukaryotic translation initiation factor 3 | 0.47 | 63.36 |
| PAAG_01951 | Eukaryotic translation initiation factor 3 subunit E | 0.24 | 79.25 |
| PAAG_06489 | Eukaryotic translation initiation factor 3 subunit 8 | 0.48 | 139.32 |
| PAAG_08817 | Translation initiation factor 2 subunit beta | 0.24 | 43.33 |
| PAAG_09045 | Eukaryotic translation initiation factor 4E-1 | 0.62 | 54.26 |
| PAAG_00594 | Elongation factor 2 | 0.57 | 857.93 |
| PAAG_00376 | Eukaryotic translation initiation factor 3 subunit F | 0.35 | 60.89 |
| PAAG_07283 | ATP-dependent RNA helicase FAL1 | 0.39 | 117.76 |
| PAAG_03652 | Cap binding protein | 0.28 | 41.56 |
| PAAG_01698 | Translation initiation factor eIF3 | 0.23 | 74.17 |
| PAAG_04511 | ATP-dependent RNA helicase SUB2 | 0.05 | 170.38 |
| PAAG_07506 | Nuclear cap-binding protein subunit 2 | 0.33 | 21.06 |
| PAAG_02828 | RNA binding domain-containing protein | 0.62 | 30.26 |
| PAAG_08680 | RNA binding domain-containing protein | 0.36 | 32.61 |
| PAAG_11075 | Eukaryotic translation initiation factor 5A | 0.48 | 26.02 |
| PAAG_11418 | Elongation factor 1-alpha | 0.40 | 464.33 |
| PAAG_03556 | Elongation factor 1 gamma domain-containing | 0.60 | 359.69 |
| PAAG_01292 | Eukaryotic peptide chain release factor | 0.39 | 89.84 |
| PAAG_00338 | Methionyl-tRNA synthetase | 0.49 | 90.53 |
| PAAG_02251 | Asparaginyl-tRNA synthetase | 0.60 | 120.98 |
|  |  |  |  |
| **6. PROTEIN FATE** | | | |
| PAAG_01727 | T-complex protein 1 subunit delta | 0.31 | 157.18 |
| PAAG_05226 | Hsp90 binding co-chaperone (Sba1) | 0.65 | 60.15 |
| PAAG_06068 | T-complex protein 1 subunit beta | 0.39 | 239.97 |
| PAAG_07165 | T-complex protein 1 subunit gamma | 0.43 | 199.20 |
| PAAG_00797 | Chaperone DnaJ | 0.30 | 113.76 |
| PAAG_07039 | Sorting nexin 3 | 0.54 | 29.09 |
| PAAG_07286 | Vacuolar protein sorting-associated protein | 0.60 | 45.42 |
| PAAG_05736 | Ubiquitin mediated proteolysis | 0.02 | 10.46 |
| PAAG_04327 | Ubiquitin carboxyl-terminal hydrolase | 0.64 | 122.33 |
| PAAG_01479 | 26S protease regulatory subunit 8 | 0.18 | 118.20 |
| PAAG_01706 | 26S proteasome regulatory subunit RPN10 | 0.61 | 12.26 |
| PAAG_02907 | 26S proteasome non-ATPase regulatory | 0.49 | 87.45 |
| PAAG_04899 | 26S protease regulatory subunit 4 | 0.43 | 40.52 |
| PAAG_08020 | 26S proteasome regulatory subunit rpn-8 | 0.34 | 133.82 |
| PAAG_00202 | Ubiquitin carboxyl-terminal hydrolase | 0.64 | 116.22 |
| PAAG_00770 | 26S protease regulatory subunit | 0.25 | 105.04 |
| PAAG_11406 | 26S protease regulatory subunit 6B | 0.64 | 118.98 |
| PAAG_01926 | 26S proteasome regulatory subunit T5 | 0.22 | 91.50 |
| PAAG_12272 | 26S proteasome regulatory subunit rpn11 | 0.67 | 29.64 |
| PAAG_11969 | Nuclear distribution protein PAC1 | 0.57 | 125.59 |
| PAAG_02865 | Translation initiation factor RLI1 | 0.58 | 136.92 |
| PAAG_03018 | 26S proteasome complex ubiquitin receptor | 0.24 | 62.92 |
|  |  |  |  |
| **7. PROTEIN WITH BINDING FUNCTION or COFACTOR REQUIREMENT** | | | |
| PAAG_05680 | NTF2 and RRM domain-containing protein | 0.33 | 22.76 |
| PAAG_05087 | RNA-binding domain-containing protein | 0.37 | 152.85 |
| PAAG_03970 | Replication factor-A protein | 0.37 | 16.42 |
| PAAG_08356 | AP-1 complex subunit beta-1 | 0.16 | 50.01 |
| PAAG_00782 | Small COPII coat GTPase sar1 | 0.52 | 91.82 |
| PAAG_01452 | ADP-ribosylation factor | 0.65 | 6.56 |
| PAAG_02820 | GTP-dependent nucleic acid-binding protein engD | 0.54 | 201.00 |
| PAAG_08028 | GTP-binding protein ypt1 | 0.47 | 97.85 |
| PAAG_08703 | Rheb small monomeric GTPase RhbA | 0.63 | 5.75 |
| PAAG_07444 | Chaperone protein dnaK | 0.53 | 60.16 |
|  |  |  |  |
| **8. CELLULAR TRANSPORT, TRANSPORT FACILITIES and TRANSPORT ROUTES** | | | |
| PAAG_04651 | GTP-binding nuclear protein GSP1/Ran | 0.58 | 239.90 |
| PAAG_04904 | ABC transporter ATP-binding protein ARB1 | 0.33 | 119.93 |
| PAAG_04953 | Reduced viability upon starvation protein | 0.58 | 58.04 |
| PAAG_12449 | Importin beta-3 subunit | 0.63 | 15.07 |
| PAAG_06657 | Importin subunit alpha-1 | 0.04 | 61.27 |
|  |  |  |  |
| **9. CELLULAR COMMUNICATION/SIGNAL TRANSDUCTION MECHANISM** | | | |
| PAAG_04197 | Suppressor of G2 allele of SKP1 | 0.61 | 32.19 |
| PAAG_03353 | Signal recognition particle 14kD protein | 0.35 | 24.53 |
| PAAG_07634 | Small GTPase RhoA | 0.58 | 121.49 |
| PAAG_02377 | Rho GDP-dissociation inhibitor | 0.62 | 173.86 |
| PAAG_07900 | Phosphatidylinositol-phosphatidylcholine transfer | 0.67 | 117.81 |
| PAAG_00783 | Serine/threonine-protein phosphatase PP1 | 0.43 | 104.62 |
| PAAG_05737 | Calcium/calmodulin-dependent protein kinase | 0.52 | 54.92 |
| PAAG_04743 | Casein kinase II subunit alpha | 0.49 | 29.14 |
| PAAG_07054 | COP9 signalosome complex subunit 7a | 0.34 | 17.97 |
| PAAG_05449 | RAC-alpha serine/threonine-protein kinase | 0.33 | 19.03 |
| PAAG_05129 | Serine/threonine protein phosphatase 2A | 0.55 | 34.11 |
| PAAG_07339 | S-phase kinase-associated protein 1A | 0.52 | 97.30 |
| PAAG_03659 | Protein kinase gsk3 | 0.63 | 112.29 |
| PAAG_03643 | 6-phosphofructo-2-kinase | 0.66 | 72.71 |
|  |  |  |  |
| **10. CELL RESCUE, DEFENSE and VIRULENCE** | | | |
| **Stress response** | | | |
| PAAG_05679 | Heat shock protein | 0.07 | 688.41 |
|  |  |  |  |
| **Detoxification** | | | |
| PAAG_03290 | Thioredoxin | 0.41 | 49.66 |
| PAAG_03216 | Mitochondrial peroxiredoxin PRX1 | 0.33 | 165.36 |
| PAAG_01849 | Glutaredoxin | 0.48 | 25.26 |
|  |  |  |  |
| **11. BIOGENESIS of CELLULAR COMPONENTS** | | | |
| **Cytoskeleton/structural proteins** | | | |
| PAAG_03532 | Actin | 0.43 | 253.78 |
| PAAG_00004 | Actin binding protein | 0.53 | 181.29 |
| PAAG_05638 | F-actin-capping protein subunit beta | 0.26 | 57.91 |
| PAAG_01272 | F-actin-capping protein subunit alpha | 0.27 | 72.11 |
| PAAG_05414 | Septum formation protein Maf | 0.27 | 17.45 |
|  |  |  |  |
| **12. MISCELLANEOUS** | | | |
| PAAG_08941 | AmmeMemoRadiSam system protein B | 0.65 | 36.22 |
| PAAG_07829 | Phosphatase family protein | 0.20 | 37.39 |
| PAAG_08992 | Ttype 2A phosphatase activator tip41 | 0.46 | 45.23 |
| PAAG_07874 | Hepatocellular carcinoma down-regulated mitochondrial | 0.21 | 43.57 |
|  |  |  |  |
| **13. UNCLASSIFIED** | | | |
| PAAG_00059 | Hypothetical protein | 0.41 | 31.08 |
| PAAG_00117 | Hypothetical protein | 0.53 | 24.78 |
| PAAG_00220 | Hypothetical protein | 0.53 | 65.34 |
| PAAG_00579 | Hypothetical protein | 0.48 | 126.90 |
| PAAG_00828 | Hypothetical protein | 0.50 | 21.21 |
| PAAG_01121 | Hypothetical protein | 0.01 | 9.88 |
| PAAG_01695 | Hypothetical protein | 0.58 | 98.52 |
| PAAG_01919 | Hypothetical protein | 0.62 | 20.42 |
| PAAG_02072 | Hypothetical protein | 0.54 | 15.78 |
| PAAG_02270 | Hypothetical protein | 0.23 | 16.00 |
| PAAG_02353 | Hypothetical protein | 0.28 | 27.45 |
| PAAG_02386 | Hypothetical protein | 0.65 | 10.25 |
| PAAG_03179 | Hypothetical protein | 0.15 | 16.84 |
| PAAG_03624 | Hypothetical protein | 0.55 | 109.99 |
| PAAG_03636 | Hypothetical protein | 0.48 | 16.55 |
| PAAG_04278 | Hypothetical protein | 0.27 | 10.74 |
| PAAG_05315 | Hypothetical protein | 0.10 | 11.67 |
| PAAG_06338 | Hypothetical protein | 0.57 | 4.54 |
| PAAG_06679 | Hypothetical protein | 0.66 | 37.78 |
| PAAG_06711 | Hypothetical protein | 0.06 | 5.21 |
| PAAG_06837 | Hypothetical protein | 0.22 | 5.93 |
| PAAG_07132 | Hypothetical protein | 0.49 | 26.37 |
| PAAG_07521 | Hypothetical protein | 0.65 | 4.69 |
| PAAG_07606 | Hypothetical protein | 0.63 | 45.73 |
| PAAG_07633 | Hypothetical protein | 0.36 | 38.69 |
| PAAG_07883 | Hypothetical protein | 0.17 | 38.79 |
| PAAG_08058 | Hypothetical protein | 0.55 | 187.52 |
| PAAG_08799 | Hypothetical protein | 0.02 | 10.44 |
| PAAG_11167 | Hypothetical protein | 0.31 | 15.93 |
| PAAG_11176 | Hypothetical protein | 0.48 | 10.65 |
| PAAG_11364 | Hypothetical protein | 0.47 | 69.22 |
| PAAG_11583 | Hypothetical protein | 0.34 | 16.82 |
| PAAG_11610 | Hypothetical protein | 0.59 | 5.67 |
| PAAG_11708 | Hypothetical protein | 0.49 | 31.87 |
| PAAG_11835 | Hypothetical protein | 0.30 | 64.31 |
| PAAG_12288 | Hypothetical protein | 0.40 | 319.63 |
| PAAG_12503 | Hypothetical protein | 0.26 | 6.28 |
| PAAG_12620 | Hypothetical protein | 0.59 | 140.68 |

^a^ Identification of differentially regulated proteins from *Paracoccidioides* genome database (http://www.broadinstitute.org/annotation/genome/paracoccidioides_brasiliensis/MultiHome.html) using the ProteinLynx Global Server vs. 2.4 (PLGS) (Waters Corporation, Manchester, UK).

^b^ Proteins annotation from *Paracoccidioides* genome database or by homology from NCBI database (<http://www.ncbi.nlm.nih.gov/>)

^c^ Acetate/Glucose means: The level of expression in yeast cells derived from cultured in sodium acetate divided by the level in the control yeast cells cultured in glucose.

^d^ Biological process of differentially expressed proteins from MIPS (http://mips.helmholtz-muenchen.de/funcatDB/) and Uniprot databases (http://www.uniprot.org/).
