# Supplementary material for: Differential Metabolism of a Two-Carbon Substrate by Members of the Paracoccidioides Genus
Source: Front Microbiol. 2017 Nov 27;8:2308. doi: 10.3389/fmicb.2017.02308 (PMC5711815; doi:10.3389/fmicb.2017.02308)
Supplement: Supplementary file 3 [file Table3.DOCX]

**Supplemental Table 3: Proteins up-regulated in** *Paracoccidioides lutzii* **after growth for 48 hours in sodium acetate as carbon source.**

| **Accession number^a^** | **Protein Description^b^** | | **Acetate/Glucose Ratio^c^** | **Score** |
| --- | --- | --- | --- | --- |
| **Functional categories^d^** | | |  |  |
| **1. METABOLISM** | | | | |
| **Amino acid metabolism** | | | | |
| PAAG_02975 | 2,4-dihydroxyhept-2-ene-1,7-dioic acid aldolase | | 2.32 | 60.36 |
| PAAG_05481 | 2-isopropylmalate synthase | | 3.32 | 81.98 |
| PAAG_01194 | 2-oxoisovalerate dehydrogenase subunit beta | | 1.64 | 81.87 |
| PAAG_03978 | 3-hydroxyisobutyrate dehydrogenase | | 1.65 | 202.80 |
| PAAG_05328 | 3-isopropylmalate dehydrogenase A | | 2.05 | 66.19 |
| PAAG_02859 | Adenosylhomocysteinase | | 1.99 | 337.84 |
| PAAG_08207 | Alanine aminotransferase | | 1.73 | 45.84 |
| PAAG_08668 | Anthranilate synthase component 2 | | 1.93 | 260.85 |
| PAAG_01969 | Arginase | | 2.42 | 39.72 |
| PAAG_01144 | Aspartate aminotransferase | | 1.81 | 64.44 |
| PAAG_08065 | Aspartate-semialdehyde dehydrogenase | | 1.67 | 158.87 |
| PAAG_09095 | ATP phosphoribosyltransferase | | 5.32 | 40.67 |
| PAAG_01365 | Choline dehydrogenase | | 1.95 | 137.69 |
| PAAG_05198 | Chorismate mutase | | 4.96 | 96.77 |
| PAAG_08405 | Cystathionine beta-lyase | | 1.73 | 89.64 |
| PAAG_05253 | Delta-1-pyrroline-5-carboxylate dehydrogenase | | 3.71 | 283.08 |
| PAAG_05776 | Dihydroxy-acid dehydratase | | 1.61 | 145.93 |
| PAAG_08163 | Fumarylacetoacetase | | 1.85 | 177.34 |
| PAAG_00869 | Fumarylacetoacetate hydrolase domain-containing protein | | 1.77 | 73.60 |
| PAAG_01649 | Gamma-butyrobetaine dioxygenase | | 2.13 | 13.51 |
| PAAG_07954 | Gamma-glutamyl phosphate reductase | | 1.74 | 160.31 |
| PAAG_08931 | Glutamate carboxypeptidase | | 1.62 | 174.62 |
| PAAG_01568 | Glycine dehydrogenase | | 1.56 | 344.68 |
| PAAG_05406 | Histidine biosynthesis trifunctional protein | | 5.41 | 374.39 |
| PAAG_08164 | Homogentisate 1,2-dioxygenase | | 2.85 | 179.08 |
| PAAG_01991 | Homoserine dehydrogenase | | 1.55 | 98.08 |
| PAAG_02644 | Kynurenine-oxoglutarate transaminase | | 3.39 | 164.08 |
| PAAG_02217 | Isochorismatase domain-containing protein | | 3.32 | 124.54 |
| PAAG_04083 | Isochorismatase family hydrolase | | 2.96 | 13.75 |
| PAAG_11161 | L-serine dehydratase | | 1.76 | 18.19 |
| PAAG_03092 | TPA: putative Conserved lysine-rich protein | | 4.41 | 179.83 |
| PAAG_08162 | Maleylacetoacetate isomerase | | 1.86 | 69.23 |
| PAAG_04103 | Methylcrotonoyl-CoA carboxylase beta chain | | 1.55 | 206.48 |
| PAAG_07036 | Methylmalonate-semialdehyde dehydrogenase | | 1.66 | 206.70 |
| PAAG_01302 | S-methyl-5'-thioadenosine phosphorylase | | 2.09 | 120.37 |
| PAAG_00047 | Porphyrin and chlorophyll metabolism | | 2.00 | 62.76 |
| PAAG_05847 | Saccharopine dehydrogenase | | 2.10 | 11.58 |
| PAAG_03168 | Threonine dehydratase | | 2.35 | 88.86 |
| PAAG_03537 | Lysine decarboxylase-like protein | | 1.72 | 92.48 |
|  |  | |  |  |
| **Nitrogen, sulfur and selenium metabolism** | | | | |
| PAAG_04233 | 2-nitropropane dioxygenase | | 1.82 | 59.48 |
| PAAG_06606 | Cyanate hydratase | | 1.87 | 56.15 |
| PAAG_03333 | Formamidase | | 2.03 | 202.65 |
| PAAG_00954 | Urease | | 1.57 | 273.15 |
|  |  | |  |  |
| **Nucleotide/nucleoside/nucleobase metabolism** | | | | |
| PAAG_07312 | CTP synthase | | 2.21 | 55.27 |
| PAAG_02333 | GMP synthase | | 1.62 | 51.87 |
| PAAG_08438 | Purine nucleoside phosphorylase | | 1.66 | 17.94 |
| PAAG_01437 | Uricase | | 2.76 | 89.60 |
| PAAG_05242 | Carbamoyl-phosphate synthase | | 1.76 | 176.96 |
| PAAG_03904 | DNA-directed RNA polymerase III subunit Rpc5 | | 2.10 | 15.40 |
| PAAG_04929 | DNA-directed RNA polymerase III subunit RPC7 | | 1.56 | 13.25 |
| PAAG_04919 | Nucleoside-diphosphate-sugar epimerase | | 2.00 | 41.97 |
|  |  | |  |  |
| **Phosphate metabolism** | | | | |
| PAAG_00661 | 4-nitrophenylphosphatase | | 1.81 | 43.02 |
| PAAG_08277 | Nitroreductase family protein | | 2.03 | 130.99 |
|  |  | |  |  |
| **C-compound and carbohydrate metabolism** | | |  |  |
| PAAG_03776 | Inositol-3-phosphate synthase | | 2.16 | 83.51 |
| PAAG_04181 | Sorbitol utilization protein SOU2 | | 1.61 | 78.13 |
| PAAG_05416 | NADP-dependent leukotriene B4 | | 1.51 | 76.89 |
| PAAG_03765 | NADP-dependent glycerol dehydrogenase | | 1.80 | 49.12 |
| PAAG_07276 | Glycogen synthase | | 2.32 | 133.32 |
| PAAG_03243 | Aldose 1-epimerase family protein | | 1.65 | 147.72 |
| PAAG_05287 | Amidohydrolase | | 1.61 | 21.87 |
|  |  | |  |  |
| **Lipid, fatty acid and isoprenoid metabolism** | | | | |
| PAAG_06329 | 3-hydroxybutyryl-CoA dehydrogenase | | 2.40 | 107.88 |
| PAAG_02664 | 3-ketoacyl-CoA thiolase | | 2.20 | 245.70 |
| PAAG_07746 | 3-ketoacyl-CoA thiolase | | 2.00 | 93.78 |
| PAAG_03447 | Acetyl-CoA acetyltransferase | | 2.17 | 145.13 |
| PAAG_05454 | Acyl-CoA dehydrogenase | | 2.00 | 211.80 |
| PAAG_03116 | Acyl-coenzyme A oxidase | | 2.46 | 89.66 |
| PAAG_06309 | Enoyl-CoA hydratase | | 1.57 | 227.79 |
| PAAG_06392 | Enoyl-CoA hydrataseily protein | | 1.57 | 29.07 |
| PAAG_05690 | Esterase D | | 2.26 | 145.43 |
| PAAG_05984 | Glutaryl-CoA dehydrogenase | | 1.56 | 210.74 |
| PAAG_03960 | Isopentenyl-diphosphate Delta-isomerase | | 1.65 | 53.10 |
| PAAG_00976 | LiPid Depleted family member | | 1.70 | 17.46 |
| PAAG_11860 | Nonspecific lipid-transfer protein _ Transport | | 1.58 | 95.15 |
| PAAG_01928 | Peroxisomal dehydratase | | 1.59 | 47.87 |
| PAAG_05093 | Succinyl-CoA:3-ketoacid-coenzyme A | | 1.68 | 205.21 |
| PAAG_01557 | Short-chain dehydrogenase | | 2.10 | 5.24 |
| PAAG_01269 | Diacylglycerol pyrophosphate phosphatase | | 1.87 | 5.37 |
|  |  | |  |  |
| **Metabolism of vitamins, cofactors, and prosthetic groups** | | | | |
| PAAG_01227 | 3-methyl-2-oxobutanoatehydroxymethyltransferase | | 2.11 | 25.62 |
| PAAG_03709 | 5-formyltetrahydrofolate cyclo-ligase | | 2.59 | 21.64 |
| PAAG_00851 | 6,7-dimethyl-8-ribityllumazine synthase | | 2.46 | 37.65 |
| PAAG_07321 | Pyridoxine biosynthesis protein PDX1 | | 1.52 | 115.30 |
| PAAG_03345 | Phosphotyrosine protein phosphatase | | 9.31 | 34.90 |
| PAAG_08427 | Aldose reductase | | 1.65 | 27.09 |
| PAAG_03106 | ThiJ/PfpI family protein | | 5.31 | 75.64 |
| PAAG_03631 | 1,2-oxophytodienoate reductase | | 1.58 | 264.85 |
| PAAG_03741 | Hydrolase-HD superfamily protein | | 5.01 | 11.17 |
| PAAG_11532 | Arylamine N-acetyltransferase 1 | | 1.90 | 23.01 |
| PAAG_06083 | Dienelactone hydrolase family protein | | 2.12 | 82.35 |
| PAAG_07875 | Lactoylglutathione lyase | | 2.07 | 27.49 |
| PAAG_00293 | Quinone oxidoreductase | | 3.31 | 83.85 |
| PAAG_00566 | Aflatoxin B1 aldehyde reductase member 2 | | 3.25 | 177.32 |
| PAAG_04966 | Hydrolase | | 1.52 | 117.50 |
|  |  | |  |  |
| **2. ENERGY** |  | |  |  |
| **Glycolysis and gluconeogenesis** | | | | |
| PAAG_02682 | Fructose-1,6-bisphosphatase | | 1.57 | 116.33 |
| PAAG_01995 | Fructose-bisphosphate aldolase | | 1.56 | 319.12 |
| PAAG_06172 | Glucokinase | | 2.18 | 178.62 |
| PAAG_08468 | Glyceraldehyde-3-phosphate dehydrogenase | | 1.53 | 373.46 |
| PAAG_11035 | Pyruvate dehydrogenase protein X component | | 1.66 | 226.61 |
| **Ethanol production** | |  |  |  |
| PAAG_00403 | Alcohol dehydrogenase | | 5.88 | 287.75 |
| PAAG_04541 | Alcohol dehydrogenase | | 6.33 | 247.18 |
| PAAG_08911 | Alcohol dehydrogenase | | 1.62 | 73.52 |
| PAAG_03910 | Aldehyde dehydrogenase | | 1.70 | 123.61 |
| PAAG_02050 | Pyruvate decarboxylase | | 1.66 | 216.82 |
|  |  | |  |  |
| **Pentose-phosphate pathway** | | | | |
| PAAG_00633 | Glucose-6-phosphate 1-dehydrogenase | | 1.77 | 153.57 |
| PAAG_05940 | Deoxyribose-phosphate aldolase | | 1.67 | 69.76 |
| PAAG_05146 | Ribose 5-phosphate isomerase A | | 1.92 | 41.82 |
|  |  | |  |  |
| **Tricarboxylic-acid pathway** | | | | |
| PAAG_02732 | 2-oxoglutarate dehydrogenase E1 | | 1.60 | 473.32 |
| PAAG_08075 | Citrate synthase | | 1.95 | 259.33 |
| PAAG_08915 | Dihydrolipoamide succinyltransferase | | 2.13 | 185.60 |
| PAAG_00588 | Fumarate hydratase | | 2.04 | 226.02 |
| PAAG_00856 | Isocitrate dehydrogenase subunit 1 | | 1.56 | 209.92 |
| PAAG_00053 | Malate dehydrogenase | | 1.59 | 426.26 |
| PAAG_01725 | Succinate dehydrogenase flavoprotein subunit | | 3.46 | 197.78 |
| PAAG_06103 | Succinate dehydrogenase iron-sulfur subunit | | 3.29 | 61.54 |
| PAAG_00417 | Succinyl-CoA ligase subunit alpha | | 1.71 | 226.10 |
| PAAG_05048 | 3-isopropylmalate dehydratase large subunit | | 2.57 | 521.25 |
|  |  | |  |  |
| **Glyoxylate cycle** | | | | |
| PAAG_06951 | Isocitrate lyase | | 2.99 | 303.75 |
| PAAG_04542 | Malate synthase | | 1.73 | 267.72 |
|  |  | |  |  |
| **Methylcytrate cycle** | | | | |
| PAAG_04550 | 2-methylcitrate synthase | | 2.67 | 426.54 |
| PAAG_04559 | 2-methylcitrate dehydratase | | 2.00 | 435.38 |
|  |  | |  |  |
| **Electron transport and membrane-associated energy conservation** | | | | |
| PAAG_05605 | ATP synthase delta chain | | 4.23 | 71.72 |
| PAAG_05576 | ATP synthase gamma chain | | 2.17 | 145.13 |
| PAAG_08037 | ATP synthase subunit beta | | 4.06 | 364.59 |
| PAAG_08551 | ATP synthase subunit g | | 2.44 | 82.59 |
| PAAG_04820 | ATPase alpha subunit | | 3.59 | 391.46 |
| PAAG_12575 | Cytochrome b5 | | 1.59 | 17.14 |
| PAAG_08088 | Cytochrome b-c1 complex subunit 2 | | 3.13 | 124.12 |
| PAAG_06268 | Cytochrome c | | 1.52 | 130.71 |
| PAAG_07246 | Cytochrome c oxidase subunit VIa | | 1.54 | 107.19 |
| PAAG_07672 | Ubiquinol-cytochrome c reductase subunit 7 | | 3.76 | 43.06 |
| PAAG_12013 | NADH dehydrogenase subunit 5 | | 1.93 | 4.76 |
|  |  | |  |  |
| **3. CELL CYCLE and DNA PROCESSING** | | | | |
| PAAG_00883 | ATP-dependent DNA helicase | | 1.52 | 134.49 |
| PAAG_00170 | Chromosome transmission fidelity protein | | 1.96 | 71.92 |
| PAAG_06491 | DNA repair and recombination protein RAD26 | | 2.31 | 60.70 |
| PAAG_07273 | DNA replication licensing factor mcm5 | | 1.51 | 154.90 |
| PAAG_01520 | DNA-binding protein HGH1 | | 2.20 | 34.09 |
| PAAG_05824 | MACRO domain-containing protein | | 2.08 | 74.54 |
| PAAG_00294 | Replication factor-A protein | | 1.85 | 83.06 |
| PAAG_06486 | DNA polymerase alpha catalytic subunit | | 9.40 | 48.89 |
| PAAG_06260 | Calcineurin binding protein | | 1.73 | 22.48 |
| PAAG_07192 | Microtubule-associated protein RP/EB family member 3 | | 3.21 | 70.37 |
| PAAG_05476 | ADP-ribosylation factor family protein | | 2.49 | 20.77 |
| PAAG_06202 | CENP-Q a CENPA-CAD centromere complex subunit | | 2.24 | 22.35 |
|  |  | |  |  |
| **4. TRANSCRIPTION** | | | | |
| PAAG_08917 | Histone H2a | | 1.91 | 62.19 |
| PAAG_08918 | Late histone H2B.L4 | | 2.01 | 80.59 |
| PAAG_07099 | Histone H3.3 | | 2.27 | 58.96 |
| PAAG_07098 | Histone H4.1 | | 1.85 | 125.77 |
| PAAG_00126 | Histone H4.2 | | 2.25 | 147.14 |
| PAAG_01558 | [CBF/NF-Y family transcription factor](https://blast.ncbi.nlm.nih.gov/Blast.cgi#alnHdr_261188929) | | 2.91 | 31.93 |
| PAAG_04814 | Nucleic acid-binding protein | | 2.42 | 171.63 |
| PAAG_04726 | Pirin | | 1.65 | 118.47 |
| PAAG_01710 | Polymerase II polypeptide D | | 1.96 | 140.07 |
| PAAG_00469 | Transcription elongation factor spt4 | | 2.38 | 16.67 |
| PAAG_02276 | ATP-dependent RNA helicase suv3 | | 1.52 | 98.19 |
| PAAG_02801 | tRNA ligase | | 1.88 | 62.55 |
| PAAG_02255 | mRNA decapping hydrolase | | 2.29 | 113.71 |
| PAAG_03277 | Pre-mRNA-processing factor 39 | | 3.86 | 94.59 |
| PAAG_06168 | Peptidyl-prolyl cis-trans isomerase cypE | | 1.74 | 78.28 |
| PAAG_11921 | Pre-mRNA-splicing factor | | 1.62 | 253.01 |
| PAAG_00714 | Nuclear transport factor 2 domain-containing | | 1.79 | 19.16 |
| PAAG_04767 | U4/U6 small nuclear ribonucleoprotein PRP4 | | 6.23 | 10.98 |
| PAAG_01394 | Poly(A) RNA polymerase cid14 | | 1.98 | 85.58 |
| PAAG_02434 | C6 zinc finger domain containing protein | | 1.79 | 64.12 |
| PAAG_04647 | RNA binding protein | | 2.57 | 34.86 |
| PAAG_00992 | Heterogeneous nuclear ribonucleoprotein HRP1 | | 2.33 | 6.29 |
|  |  | |  |  |
| **5. PROTEIN SYNTHESIS** | | | | |
| PAAG_06367 | 40S ribosomal protein S11 | | 1.56 | 139.88 |
| PAAG_04965 | 60S ribosomal protein L31 | | 1.78 | 115.41 |
| PAAG_07550 | 60S ribosomal protein L42 | | 1.57 | 79.91 |
| PAAG_01412 | 40S ribosomal protein S17 | | 2.05 | 74.55 |
| PAAG_08153 | 50S ribosomal protein L6 | | 3.07 | 25.83 |
| PAAG_08285 | 50S ribosomal protein L12 | | 1.78 | 131.23 |
| PAAG_01731 | 54S ribosomal protein L37 | | 1.60 | 31.99 |
| PAAG_00302 | Exportin-1 | | 2.22 | 82.74 |
| PAAG_00747 | Eukaryotic translation initiation factor 2 subunit gamma | | 1.60 | 173.40 |
| PAAG_02582 | Elongation factor 2 | | 2.78 | 57.31 |
| PAAG_07381 | Eukaryotic peptide chain release factor subunit 1 | | 2.03 | 93.13 |
| PAAG_02071 | Glutamyl-tRNA synthetase | | 2.46 | 334.35 |
| PAAG_08702 | Seryl-tRNA synthetase | | 1.82 | 207.91 |
| PAAG_05103 | Threonyl-tRNA synthetase | | 1.73 | 101.26 |
| PAAG_04742 | Valyl-tRNA synthetase | | 1.72 | 318.84 |
| PAAG_05969 | Translation initiation factor 2A | | 2.77 | 18.49 |
| PAAG_00772 | Translation initiation factor 3 subunit J | | 2.52 | 100.81 |
|  |  | |  |  |
| **6. PROTEIN FATE** | | | | |
| PAAG_01726 | 26S protease regulatory subunit 8 | | 2.36 | 93.03 |
| PAAG_03191 | 26S proteasome non-ATPase regulatory subunit 1 | | 1.72 | 125.14 |
| PAAG_05962 | 26S proteasome non-ATPase regulatory subunit 3 | | 2.10 | 143.38 |
| PAAG_05029 | 26S proteasome regulatory subunit RPN9 | | 1.63 | 84.03 |
| PAAG_02026 | Ankyrin repeat protein | | 2.23 | 19.75 |
| PAAG_02772 | Endosomal peripheral membrane protein | | 19.29 | 53.87 |
| PAAG_09122 | FK506-binding protein | | 1.86 | 31.95 |
| PAAG_02974 | Glutaredoxin domain-containing protein | | 3.27 | 41.85 |
| PAAG_01854 | [Golgi apparatus membrane protein TVP18](https://blast.ncbi.nlm.nih.gov/Blast.cgi#alnHdr_821504707) | | 1.70 | 46.95 |
| PAAG_03161 | GTP-binding protein ypt5 | | 2.40 | 79.88 |
| PAAG_06255 | Mitochondrial co-chaperone GrpE | | 2.26 | 194.69 |
| PAAG_03772 | Mitochondrial import inner translocase subunit tim9 | | 1.64 | 27.00 |
| PAAG_00109 | Mitochondrial intermembrane subunit Tim | | 5.69 | 6.03 |
| PAAG_05417 | Mitochondrial-processing peptidase subunit beta | | 1.90 | 175.07 |
| PAAG_07490 | Monothiol glutaredoxin-5 | | 1.83 | 84.84 |
| PAAG_05788 | Peptidyl-prolyl cis-trans isomerase A2 | | 1.68 | 152.72 |
| PAAG_00739 | Peptidyl-prolyl cis-trans isomerase B | | 3.25 | 112.77 |
| PAAG_07509 | Peptidyl-prolyl cis-trans isomerase ssp1 | | 1.53 | 79.96 |
| PAAG_06250 | Peptidylprolyl isomerase | | 1.82 | 35.93 |
| PAAG_03101 | Prefoldin subunit 1 | | 1.73 | 69.84 |
| PAAG_00852 | Proteasome component C1 | | 1.60 | 134.41 |
| PAAG_03536 | Proteasome component PRE5 | | 1.58 | 142.38 |
| PAAG_00071 | Proteasome component Y7 | | 1.58 | 136.20 |
| PAAG_08141 | Proteasome subunit alpha type-4 | | 1.58 | 135.25 |
| PAAG_11221 | Pyroglutamyl peptidase type | | 1.59 | 33.40 |
| PAAG_12392 | T-complex protein 1 subunit zeta | | 4.80 | 100.78 |
| PAAG_00295 | Ubiquitin carboxyl-terminal hydrolase | | 2.23 | 132.99 |
| PAAG_02254 | Ubiquitin carboxyl-terminal hydrolase | | 1.86 | 65.44 |
| PAAG_04282 | UBX domain-containing protein | | 1.64 | 51.91 |
| PAAG_07890 | Vacuolar-sorting protein snf7_Endocytosis | | 1.83 | 23.51 |
| PAAG_07500 | Xaa-Pro aminopeptidase | | 4.52 | 130.01 |
| PAAG_00664 | Aspartyl aminopeptidase | | 1.54 | 193.14 |
|  |  | |  |  |
| **7. PROTEIN WITH BINDING FUNCTION or COFACTOR REQUIREMENT** | | | | |
| PAAG_11982 | YbgI/family dinuclear metal center protein | | 2.49 | 17.59 |
| PAAG_04391 | Progesterone binding protein | | 3.95 | 92.85 |
| PAAG_08648 | GTP-binding protein RBG1 | | 2.63 | 41.64 |
| PAAG_11679 | Chaperone protein clpB | | 1.97 | 172.93 |
|  |  | |  |  |
| **8. CELLULAR TRANSPORT, TRANSPORT FACILITIES and TRANSPORT ROUTES** | | | | |
| PAAG_00555 | Coatomer subunit epsilon | | 3.47 | 23.96 |
| PAAG_08359 | Coatomer subunit alpha | | 1.72 | 170.24 |
| PAAG_08103 | EF hand domain-containing protein | | 7.40 | 66.14 |
| PAAG_01500 | GTP-binding protein SAS1 | | 2.05 | 55.97 |
| PAAG_08093 | GTP-binding protein ypt3 | | 1.99 | 159.56 |
| PAAG_00326 | Heavy metal ion transporter, putative | | 1.57 | 28.57 |
| PAAG_12133 | Lactose permease | | 8.25 | 10.93 |
| PAAG_06752 | Mitochondrial ATPase inhibitor, IATP family protein | | 2.76 | 69.19 |
| PAAG_04243 | Na+/K+-exchanging ATPase alpha chain | | 2.04 | 47.88 |
| PAAG_04276 | Phosphatidylinositol transporter | | 1.89 | 94.54 |
| PAAG_07341 | Transport protein particle subunit bet5 | | 9.74 | 15.65 |
| PAAG_12424 | Voltage-dependent anion channel protein 1 | | 1.51 | 12.17 |
|  |  | |  |  |
| **9. CELLULAR COMMUNICATION/SIGNAL TRANSDUCTION MECHANISM** | | | | |
| PAAG_00332 | cAMP-regulated phosphoprotein family protein Igo1 | | 1.61 | 45.71 |
| PAAG_02599 | Phosphorelay intermediate protein YPD1 | | 1.50 | 28.29 |
| PAAG_01602 | Ras-like GTP-binding protein | | 4.20 | 19.24 |
| PAAG_04274 | Protein phosphatase inhibitor 2 (IPP-2) | | 4.58 | 46.79 |
|  |  | |  |  |
| **10. CELL RESCUE, DEFENSE and VIRULENCE** | | | | |
| **Stress response** | | | | |
| PAAG_00871 | 30 kDa heat shock protein | | 3.43 | 223.90 |
| PAAG_08059 | Heat shock protein | | 2.25 | 900.66 |
| PAAG_01262 | Hsp70-like protein | | 1.53 | 456.40 |
| PAAG_11262 | Hsp7-like protein | | 1.69 | 549.72 |
| PAAG_05142 | 10 kDa heat shock protein | | 2.26 | 164.83 |
|  |  | |  |  |
| **Detoxification** | | | | |
| PAAG_01454 | Catalase | | 2.72 | 334.92 |
| PAAG_05860 | Glutamate-cysteine ligase | | 5.56 | 28.19 |
| PAAG_04164 | Superoxide dismutase Cu/Zn SOD1 | | 3.38 | 58.63 |
| PAAG_02725 | Superoxide dismutase Fe/Mn SOD2 | | 3.71 | 153.18 |
| PAAG_02926 | Superoxide dismutase Fe/Mn SOD5 | | 2.55 | 98.31 |
| PAAG_05292 | Glutathione reductase | | 1.61 | 153.12 |
| PAAG_03931 | Glutathione S-transferase Gst3 | | 4.71 | 90.39 |
|  |  | |  |  |
| **11. BIOGENESIS OF CELLULAR COMPONENTS** | | | | |
| **Cell wall** |  | |  |  |
| PAAG_00651 | Alpha-1,3-glucan synthase | | 3.04 | 11.53 |
| PAAG_01874 | Dolichol-phosphate mannosyltransferase | | 1.58 | 23.58 |
| PAAG_01136 | Beta-hexosaminidase | | 4.81 | 46.84 |
| PAAG_08038 | 1,4-alpha-glucan-branching enzyme | | 1.94 | 144.62 |
|  |  | |  |  |
| **Cytoskeleton/strucutural proteins** | | | | |
| PAAG_00564 | Actin | | 1.96 | 106.74 |
| PAAG_07185 | Actin like protein 2/3 complex, subunit 5 | | 5.14 | 69.65 |
| PAAG_08252 | Clathrin light chain | | 1.64 | 101.04 |
| PAAG_00529 | Cytoskeletal adaptor protein SagA | | 1.52 | 121.01 |
| PAAG_07958 | Fimbrin | | 1.62 | 219.14 |
|  |  | |  |  |
| **12. MISCELLANEOUS** | | | | |
| PAAG_05905 | Programmed cell death protein 5 | | 2.34 | 21.95 |
| PAAG_00493 | Autophagy-related protein 3 | | 2.36 | 64.46 |
| PAAG_00008 | Alpha/beta hydrolase fold family protein | | 1.84 | 21.77 |
| PAAG_02650 | C2 domain-containing protein | | 2.66 | 47.03 |
| PAAG_06512 | CHCH domain-containing protein | | 1.68 | 24.79 |
| PAAG_03152 | CobW domain-containing protein | | 1.53 | 170.14 |
| PAAG_00367 | CUE domain-containing protein | | 2.69 | 35.59 |
| PAAG_04610 | DUF28 domain-containing protein | | 1.51 | 23.30 |
| PAAG_04422 | EKC/KEOPS complex, subunit Pcc1 | | 1.98 | 10.55 |
| PAAG_02068 | Elicitor protein | | 1.82 | 59.29 |
| PAAG_03427 | Endonuclease/exonuclease/phosphatase | | 3.23 | 4.70 |
| PAAG_00503 | HAD-superfamily hydrolase | | 3.02 | 181.77 |
| PAAG_05037 | HHE domain-containing protein | | 2.68 | 24.15 |
| PAAG_01976 | LEA domain protein | | 2.64 | 57.47 |
| PAAG_01717 | MGS207 protein | | 1.89 | 46.16 |
| PAAG_07872 | MS8 | | 2.16 | 45.34 |
| PAAG_07821 | NAD dependent epimerase | | 2.12 | 97.47 |
| PAAG_04851 | Osmotic growth protein | | 1.81 | 242.10 |
| PAAG_05720 | SAP domain-containing protein | | 2.03 | 43.01 |
| PAAG_03330 | Dihydrolipoyl dehydrogenase | | 1.68 | 332.08 |
| PAAG_01556 | FAD dependent oxidoreductase superfamily | | 1.52 | 73.84 |
| PAAG_03774 | S-(hydroxymethyl)glutathione dehydrogenase | | 2.08 | 44.92 |
|  |  | |  |  |
|  |  | |  |  |
| **13. UNCLASSIFIED** | | | | |
| PAAG_00297 | Hypothetical protein | | 1.67 | 97.23 |
| PAAG_00335 | Hypothetical protein | | 1.56 | 115.90 |
| PAAG_00340 | Hypothetical protein | | 2.42 | 199.39 |
| PAAG_00835 | Hypothetical protein | | 2.14 | 26.12 |
| PAAG_01284 | Hypothetical protein | | 2.48 | 6.39 |
| PAAG_01952 | Hypothetical protein | | 1.76 | 24.79 |
| PAAG_02001 | Hypothetical protein | | 2.55 | 36.96 |
| PAAG_02121 | Hypothetical protein | | 2.80 | 16.92 |
| PAAG_02336 | Hypothetical protein | | 3.47 | 97.80 |
| PAAG_02375 | Hypothetical protein | | 4.39 | 12.29 |
| PAAG_03072 | Hypothetical protein | | 1.68 | 10.84 |
| PAAG_03648 | Hypothetical protein | | 1.77 | 11.51 |
| PAAG_03740 | Hypothetical protein | | 2.57 | 83.52 |
| PAAG_03925 | Hypothetical protein | | 6.85 | 26.26 |
| PAAG_04303 | Hypothetical protein | | 5.68 | 31.87 |
| PAAG_04335 | Hypothetical protein | | 2.65 | 31.38 |
| PAAG_05351 | Hypothetical protein | | 1.74 | 16.28 |
| PAAG_05403 | Hypothetical protein | | 1.51 | 10.37 |
| PAAG_05565 | Hypothetical protein | | 2.77 | 6.84 |
| PAAG_05624 | Hypothetical protein | | 6.39 | 22.79 |
| PAAG_05856 | Hypothetical protein | | 16.67 | 14.91 |
| PAAG_05957 | Hypothetical protein | | 1.69 | 34.28 |
| PAAG_06248 | Hypothetical protein | | 4.29 | 18.33 |
| PAAG_06376 | Hypothetical protein | | 1.51 | 5.44 |
| PAAG_06851 | Hypothetical protein | | 2.86 | 27.69 |
| PAAG_08671 | Hypothetical protein | | 2.29 | 84.51 |
| PAAG_11138 | Hypothetical protein | | 12.27 | 9.89 |
| PAAG_11142 | Hypothetical protein | | 2.34 | 22.05 |
| PAAG_11323 | Hypothetical protein | | 2.11 | 16.44 |
| PAAG_11405 | Hypothetical protein | | 2.63 | 5.91 |
| PAAG_11937 | Hypothetical protein | | 1.85 | 5.82 |
| PAAG_11980 | Hypothetical protein | | 1.67 | 6.46 |
| PAAG_12043 | Hypothetical protein | | 1.59 | 22.08 |
| PAAG_12169 | Hypothetical protein | | 2.35 | 24.31 |
| PAAG_12334 | Hypothetical protein | | 4.83 | 17.69 |
| PAAG_12439 | Hypothetical protein | | 1.82 | 21.67 |
| PAAG_12504 | Hypothetical protein | | 2.38 | 16.99 |
| PAAG_12514 | Hypothetical protein | | 1.72 | 11.91 |
| PAAG_12554 | Hypothetical protein | | 1.63 | 10.92 |
| PAAG_12676 | Hypothetical protein | | 4.51 | 23.59 |

^a^ Identification of differentially regulated proteins from *Paracoccidioides* genome database (http://www.broadinstitute.org/annotation/genome/paracoccidioides_brasiliensis/MultiHome.html) using the ProteinLynx Global Server vs. 2.4 (PLGS) (Waters Corporation, Manchester, UK).

^b^ Proteins annotation from *Paracoccidioides* genome database or by homology from NCBI database (<http://www.ncbi.nlm.nih.gov/>)

^c^ Acetate/Glucose means: The level of expression in yeast cells derived from cultured in sodium acetate divided by the level in the control yeast cells cultured in glucose.

^d^ Biological process of differentially expressed proteins from MIPS (http://mips.helmholtz-muenchen.de/funcatDB/) and Uniprot databases (http://www.uniprot.org/).
