# Supplementary material for: Differential Metabolism of a Two-Carbon Substrate by Members of the Paracoccidioides Genus
Source: Front Microbiol. 2017 Nov 27;8:2308. doi: 10.3389/fmicb.2017.02308 (PMC5711815; doi:10.3389/fmicb.2017.02308)
Supplement: Supplementary file 4 [file Table4.DOCX]

**Supplemental Table 4: Proteins down-regulated in** *Paracoccidioides brasiliensis* **isolate 03 after growth for 48 hours in sodium acetate as carbon source.**

| **Accession number^a^** | **Protein Description^b^** | **Acetate/Glucose Ratio^c^** | **Score** |
| --- | --- | --- | --- |
| **Functional categories^d^** | |  |  |
| **1. METABOLISM** | | | |
| **Amino acid metabolism** | | | |
| PABG_06909 | 1,2-dihydroxy-3-keto-5-methylthiopentenedioxygenase | 0.23 | 11.72 |
| PABG_01773 | 2-oxoisovalerate dehydrogenase subunit beta | 0.56 | 120.02 |
| PABG_00108 | 3-hydroxyanthranilate 3,4-dioxygenase | 0.19 | 45.89 |
| PABG_11179 | 3-isopropylmalate dehydratase, large subunit | 0.41 | 146.54 |
| PABG_02157 | Acetolactate synthase | 0.37 | 122.57 |
| PABG_07568 | Acetolactate synthase small subunit | 0.22 | 175.79 |
| PABG_03090 | Aromatic-amino-acid aminotransferase | 0.66 | 299.19 |
| PABG_02890 | Aspartate aminotransferase | 0.45 | 284.18 |
| PABG_12180 | Aspartate kinase | 0.59 | 63.83 |
| PABG_07381 | Bisphosphate-3'-nucleotidase | 0.52 | 147.20 |
| PABG_04202 | Branched-chain-amino-acid aminotransferase | 0.55 | 190.07 |
| PABG_04676 | Cysteine synthase | 0.59 | 296.70 |
| PABG_02415 | Histidinol-phosphate aminotransferase | 0.53 | 34.25 |
| PABG_03086 | Homocitrate synthase | 0.28 | 145.45 |
| PABG_07083 | Ketol-acid reductoisomerase | 0.40 | 419.20 |
| PABG_03730 | L-ornithine 5-monooxygenase | 0.02 | 241.80 |
| PABG_00309 | Methionine adenosyltransferase 2 subunit beta | 0.64 | 69.10 |
| PABG_01063 | Peptide methionine sulfoxide reductase | 0.53 | 100.68 |
| PABG_11793 | N-acetyltransferase ats1 | 0.57 | 17.95 |
| PABG_01248 | NAD-specific glutamate dehydrogenase | 0.55 | 552.67 |
| PABG_01996 | Phospho-2-dehydro-3-deoxyheptonate aldolase | 0.64 | 71.04 |
| PABG_06940 | Phosphoserine aminotransferase | 0.57 | 168.29 |
| PABG_04656 | Serine hydroxymethyltransferase | 0.50 | 213.34 |
| PABG_07147 | Serine hydroxymethyltransferase | 0.54 | 219.71 |
|  |  |  |  |
| **Nitrogen, sulfur and selenium metabolism** | | | |
| PABG_05255 | Urease accessory protein ureG | 0.48 | 59.38 |
| PABG_01063 | Peptide methionine sulfoxide reductase | 0.53 | 100.68 |
|  |  |  |  |
| **Nucleotide/nucleoside/nucleobase metabolism** | | | |
| PABG_01175 | Adenine phosphoribosyltransferase | 0.25 | 67.51 |
| PABG_05356 | Adenylate kinase isoenzyme 2, mitochondrial | 0.43 | 66.20 |
| PABG_07789 | Bifunctional dihydrofolate reductase-thymidylate synthase | 0.31 | 33.70 |
| PABG_06118 | GMP synthase | 0.46 | 52.44 |
| PABG_05573 | Non-canonical purine NTP pyrophosphatase | 0.59 | 14.83 |
| PABG_00254 | Nucleoside-diphosphate-sugar epimerase | 0.34 | 62.39 |
| PABG_03400 | Nucleolysin TIA-1 | 0.49 | 55.81 |
| PABG_07260 | Orotidine 5'-phosphate decarboxylase | 0.47 | 98.75 |
| PABG_05432 | Phosphoribosylformylglycinamidine synthase | 0.54 | 217.10 |
| PABG_01097 | UTP-glucose-1-phosphate uridylyltransferase | 0.46 | 256.19 |
|  |  |  |  |
| **C-compound and carbohydrate metabolism** | | | |
| PABG_01560 | 4-nitrophenylphosphatase | 0.36 | 63.15 |
| PABG_02592 | Epoxide hydrolase | 0.46 | 28.74 |
| PABG_03197 | Mannose-1-phosphate guanyltransferase | 0.50 | 143.35 |
| PABG_03225 | Glyoxylate reductase | 0.11 | 22.07 |
| PABG_04017 | 2-nitropropane dioxygenase | 0.55 | 71.46 |
| PABG_04506 | Alpha,alpha-trehalose-phosphate synthase | 0.19 | 37.58 |
| PABG_05374 | 2,5-diketo-D-gluconic acid reductase A | 0.59 | 33.73 |
| PABG_07675 | Sterigmatocystin 8-O-methyltransferase | 0.12 | 166.61 |
| PABG_07861 | Glycogen synthase kinase-3 beta | 0.25 | 142.36 |
|  |  |  |  |
| **Lipid, fatty acid and isoprenoid metabolism** | | | |
| PABG_03423 | Acetoacetyl-CoA synthase | 0.48 | 119.14 |
| PABG_07087 | Delta(3,5)-Delta(2,4)-dienoyl-CoA isomerase | 0.51 | 68.68 |
| PABG_07557 | Diphosphomevalonate decarboxylase | 0.47 | 69.71 |
| PABG_03585 | Fatty acid synthase subunit beta dehydratase | 0.58 | 617.58 |
| PABG_06772 | Geranylgeranyl pyrophosphate synthetase | 0.48 | 61.18 |
| PABG_07800 | Peroxisomal multifunctional enzyme | 0.19 | 555.41 |
| PABG_01991 | 3-ketoacyl-CoA thiolase | 0.62 | 42.84 |
|  |  |  |  |
| **Metabolism of vitamins, cofactors, and prosthetic groups** | | | |
| PABG_04074 | Methylenetetrahydrofolate reductase | 0.19 | 116.91 |
| PABG_07767 | Ethanolamine utilization protein eutG | 0.47 | 141.06 |
|  |  |  |  |
| **2. ENERGY** | | | |
| **Glycolysis and gluconeogenesis** | | | |
| PABG_03640 | 6-phosphofructokinase subunit beta | 0.66 | 230.81 |
| PABG_12188 | Aldose 1-epimerase | 0.61 | 52.05 |
| PABG_03332 | Phosphoglycerate kinase | 0.41 | 347.11 |
| PABG_02300 | Pyruvate decarboxylase | 0.66 | 267.86 |
|  |  |  |  |
| **Pentose-phosphate pathway** | | | |
| PABG_01586 | Glucose-6-phosphate 1-dehydrogenase | 0.41 | 172.63 |
| PABG_05454 | Ribokinase | 0.60 | 28.91 |
|  |  |  |  |
| **Tricarboxylic-acid pathway** | | | |
| PABG_04595 | ATP citrate lyase subunit | 0.56 | 109.54 |
| PABG_03242 | Dihydrolipoamide branched chain transacylase | 0.35 | 110.16 |
| PABG_04020 | Succinate dehydrogenase flavoprotein subunit | 0.60 | 109.47 |
|  |  |  |  |
| **Electron transport and membrane-associated energy conservation** | | | |
| PABG_02983 | Mitochondrial ribosomal protein subunit S24 | 0.53 | 40.17 |
| PABG_07006 | Regulatory protein suaprga1 | 0.61 | 266.45 |
| PABG_06504 | Vacuolar ATP synthase subunit E | 0.55 | 109.50 |
| PABG_05301 | Vacuolar ATP synthase subunit B | 0.43 | 213.75 |
| PABG_07507 | Cytochrome c oxidase assembly factor 6 | 0.54 | 78.25 |
|  |  |  |  |
| **3. CELL CYCLE AND and PROCESSING** | | | |
| PABG_04022 | Ankyrin repeat protein | 0.61 | 46.20 |
| PABG_04744 | Cell division control protein | 0.21 | 9.90 |
| PABG_04850 | GMF family protein | 0.29 | 18.13 |
| PABG_07186 | Mitochondrial genome maintenance protein Mgr2 | 0.60 | 26.18 |
| PABG_02962 | Mitogen-activated protein kinase | 0.63 | 105.14 |
| PABG_06608 | Neuronal-specific septin-3 | 0.27 | 153.40 |
| PABG_05575 | Nucleosome 6 protein | 0.46 | 114.72 |
| PABG_05403 | Septin-4 | 0.48 | 93.84 |
| PABG_03696 | Tubulin alpha-2 chain | 0.63 | 123.44 |
| PABG_00486 | Tubulin beta chain | 0.20 | 70.65 |
| PABG_00273 | UV excision repair protein Rad23 | 0.53 | 53.41 |
| PABG_12413 | DUF89 domain-containing protein | 0.66 | 164.34 |
|  |  |  |  |
| **4. TRANSCRIPTION** | | | |
| PABG_11363 | ATP-dependent RNA helicase Eif4a | 0.28 | 117.95 |
| PABG_11364 | ATP-dependent RNA helicase eIF4A | 0.11 | 16.79 |
| PABG_04293 | ATP-dependent RNA helicase sub2 | 0.43 | 108.63 |
| PABG_05371 | Cellular nucleic acid-binding protein | 0.49 | 122.42 |
| PABG_03581 | DNA-binding protein HGH1 | 0.59 | 43.10 |
| PABG_11509 | Elongation factor 1-alpha | 0.35 | 359.10 |
| PABG_02107 | Mediator-RNA polymerase II transcription subunit 10 | 0.27 | 5.65 |
| PABG_01229 | Mitochondrial transcription factor 1 | 0.51 | 189.29 |
| PABG_01162 | mRNA binding post-transcriptional regulator (Csx1) | 0.36 | 84.51 |
| PABG_04343 | Nascent polypeptide-associated complex subunit alpha | 0.56 | 67.83 |
| PABG_04281 | Nascent polypeptide-associated complex subunit beta | 0.53 | 60.11 |
| PABG_04720 | Peroxiredoxin Q/BCP | 0.49 | 112.27 |
| PABG_01645 | Small nuclear ribonucleoprotein Sm D1 | 0.43 | 19.49 |
| PABG_00932 | Transcription initiation factor IIA gamma chain | 0.55 | 11.79 |
| PABG_04830 | U2 small nuclear ribonucleoprotein A | 0.27 | 11.84 |
|  |  |  |  |
| **5. PROTEIN SYNTHESIS** | | | |
| PABG_00387 | 54S ribosomal protein L3 | 0.67 | 78.63 |
| PABG_06347 | 40S ribosomal protein S19 | 0.66 | 147.03 |
| PABG_02155 | Ribosomal protein L28/L40 | 0.63 | 22.59 |
| PABG_02911 | 40S ribosomal protein S12 | 0.63 | 72.80 |
| PABG_07658 | 40S ribosomal protein S2 | 0.56 | 189.04 |
| PABG_01950 | 50S ribosomal protein L13 | 0.53 | 29.45 |
| PABG_01430 | 60S acidic ribosomal protein P0 | 0.35 | 160.38 |
| PABG_05118 | 60S ribosomal protein L3 | 0.46 | 5.55 |
| PABG_11216 | 60S ribosomal protein L37 | 0.60 | 90.44 |
| PABG_06660 | Eukaryotic translation initiation factor 3 subunit F | 0.50 | 66.54 |
| PABG_03044 | Mitochondrial 54S ribosomal protein YmL36 | 0.56 | 11.00 |
| PABG_03981 | Cysteinyl-tRNA synthetase | 0.38 | 51.51 |
| PABG_06894 | Elongation factor 1-gamma 1 | 0.63 | 287.29 |
| PABG_05335 | Elongation factor 2 | 0.43 | 586.23 |
| PABG_00251 | Elongation factor G | 0.54 | 64.06 |
| PABG_05563 | Eukaryotic peptide chain release factor subunit 1 | 0.57 | 84.41 |
| PABG_01480 | Eukaryotic translation initiation factor 2 subunit 3 | 0.48 | 89.03 |
| PABG_07154 | Eukaryotic translation initiation factor 3 subunit K | 0.42 | 16.34 |
| PABG_02322 | Glutamyl-tRNA synthetase | 0.48 | 223.86 |
| PABG_04136 | Ubiquitin-conjugating enzyme | 0.42 | 5.95 |
| PABG_06435 | Initiation factor 5A-4 | 0.59 | 47.56 |
| PABG_04471 | Leucyl-tRNA synthetase | 0.64 | 220.71 |
| PABG_06626 | Methionyl-tRNA synthetase | 0.61 | 132.63 |
| PABG_01455 | Translation initiation factor 3 subunit J | 0.36 | 69.21 |
|  |  |  |  |
| **6. PROTEIN FATE** | | | |
| PABG_07221 | Monothiol glutaredoxin-5 | 0.60 | 104.02 |
| PABG_07175 | Chaperone protein dnaK | 0.65 | 125.30 |
| PABG_06307 | Calcium/calmodulin-dependent protein kinase | 0.45 | 68.52 |
| PABG_00950 | Ubiquitin-activating enzyme E1 Y | 0.57 | 310.78 |
| PABG_11351 | 26S protease regulatory subunit 10B | 0.39 | 111.68 |
| PABG_02198 | 26S protease regulatory subunit 6A | 0.32 | 88.15 |
| PABG_03773 | 26S protease regulatory subunit 7 | 0.63 | 70.22 |
| PABG_03369 | 26S proteasome non-ATPase regulatory subunit 10 | 0.58 | 105.85 |
| PABG_05889 | 26S proteasome non-ATPase regulatory subunit 11 | 0.57 | 47.81 |
| PABG_05717 | 26S proteasome non-ATPase regulatory subunit 12 | 0.41 | 51.83 |
| PABG_05698 | 26S proteasome non-ATPase regulatory subunit 3 | 0.53 | 127.20 |
| PABG_01672 | Prolyl peptidase | 0.64 | 107.27 |
| PABG_01465 | Proteasome subunit beta type-3 | 0.63 | 44.40 |
| PABG_06432 | Polyadenylate-binding protein, cytoplasmic and nuclear | 0.66 | 384.29 |
|  |  |  |  |
| **7. PROTEIN WITH BINDING FUNCTION OR COFACTOR REQUIREMENT** | | | |
| PABG_06250 | NTF2 and RRM domain-containing protein | 0.48 | 50.86 |
| PABG_01158 | Zinc finger protein GIS2 | 0.59 | 51.25 |
| PABG_05824 | Glycolipid transfer protein HET-C2 | 0.65 | 107.15 |
| PABG_02905 | GTP-binding protein 128up | 0.64 | 52.98 |
| PABG_03284 | GTP-dependent nucleic acid-binding protein engD | 0.36 | 154.36 |
| PABG_07851 | GTP-binding protein ypt1 | 0.46 | 74.35 |
|  |  |  |  |
| **8. CELLULAR TRANSPORT, TRANSPORT FACILITIES and TRANSPORT ROUTES** | | | |
| PABG_04417 | GTP-binding nuclear protein GSP1/Ran | 0.22 | 175.04 |
| PABG_04401 | Transmembrane 9 superfamily protein | 0.08 | 12.75 |
| PABG_04567 | Coatomer subunit gamma-2 | 0.52 | 49.85 |
| PABG_07340 | EF hand domain-containing protein | 0.61 | 109.59 |
| PABG_03328 | Aliphatic sulfonates import ATP-binding protein ssuB 1 | 0.63 | 114.21 |
| PABG_02315 | Peroxisomal membrane protein receptor Pex19 | 0.45 | 11.98 |
| PABG_01589 | Cation transporter ChaC | 0.51 | 16.67 |
| PABG_07473 | Vacuolar-sorting protein snf7 | 0.21 | 35.99 |
|  |  |  |  |
| **9. CELLULAR COMMUNICATION/SIGNAL TRANSDUCTION MECHANISM** | | | |
| PABG_06708 | Adenosine kinase | 0.64 | 109.86 |
| PABG_02751 | Rab GDP-dissociation inhibitor | 0.58 | 278.20 |
| PABG_11289 | Tyrosine-protein kinase | 0.57 | 21.16 |
|  |  |  |  |
| **10. CELL RESCUE, DEFENSE AND VIRULENCE** | | | |
| **Stress response** | | | |
| PABG_00736 | DNA damage-inducible protein | 0.48 | 104.96 |
| PABG_06249 | Heat shock protein | 0.14 | 879.10 |
|  |  |  |  |
| **Detoxification** | | | |
| PABG_00718 | Thioredoxin | 0.58 | 47.81 |
| PABG_03023 | Thioredoxin reductase | 0.52 | 101.38 |
|  |  |  |  |
| **11. BIOGENESIS OF CELLULAR COMPONENTS** | | | |
| **Cell wall** | | | |
| PABG_11875 | Alpha 1,3-glucosidase | 0.47 | 48.58 |
| PABG_05175 | N-acetylglucosamine-induced protein 1 | 0.51 | 15.98 |
|  |  |  |  |
| **Cytoskeleton/structural proteins** | | | |
| PABG_05049 | Coronin-6 | 0.63 | 213.15 |
| PABG_06210 | F-actin-capping protein subunit beta | 0.52 | 29.41 |
| PABG_06519 | Chitin biosynthesis protein | 0.61 | 94.35 |
| PABG_06867 | Actin-66 | 0.13 | 236.57 |
| PABG_04150 | GPI-anchored cell wall protein | 0.10 | 39.24 |
|  |  |  |  |
| **12. MISCELLANEOUS** | | | |
| PABG_01116 | Molybdopterin synthase small subunit CnxG | 0.54 | 11.63 |
| PABG_02713 | PCI domain-containing protein | 0.38 | 96.05 |
| PABG_05425 | NAP family protein | 0.55 | 58.11 |
| PABG_11354 | Zinc metalloprotease | 0.62 | 45.32 |
| PABG_04682 | Type 2A phosphatase activator tip41 | 0.35 | 44.69 |
| PABG_07380 | VHS domain-containing protein | 0.24 | 33.10 |
|  |  |  |  |
| **13. UNCLASSIFIED** | | | |
| PABG_05526 | Hypothetical protein | 0.55 | 27.23 |
| PABG_00682 | Hypothetical protein | 0.64 | 161.75 |
| PABG_01378 | Hypothetical protein | 0.27 | 10.73 |
| PABG_07518 | Hypothetical protein | 0.41 | 19.97 |
| PABG_07299 | Hypothetical protein | 0.35 | 117.35 |
| PABG_03948 | Hypothetical protein | 0.29 | 129.65 |
| PABG_00435 | Hypothetical protein | 0.24 | 19.02 |
| PABG_07406 | Hypothetical protein | 0.10 | 18.11 |
| PABG_03302 | Hypothetical protein | 0.63 | 54.55 |
| PABG_01272 | Hypothetical protein | 0.14 | 11.18 |
| PABG_01577 | Hypothetical protein | 0.42 | 4.70 |
| PABG_02043 | Hypothetical protein | 0.64 | 17.76 |
| PABG_02066 | Hypothetical protein | 0.45 | 16.11 |
| PABG_02498 | Hypothetical protein | 0.30 | 12.58 |
| PABG_03079 | Hypothetical protein | 0.45 | 46.30 |
| PABG_03144 | Hypothetical protein | 0.30 | 10.89 |
| PABG_03291 | Hypothetical protein | 0.52 | 10.80 |
| PABG_03565 | Hypothetical protein | 0.36 | 49.64 |
| PABG_03721 | Hypothetical protein | 0.19 | 15.13 |
| PABG_03735 | Hypothetical protein | 0.60 | 5.35 |
| PABG_03992 | Hypothetical protein | 0.47 | 11.18 |
| PABG_04002 | Hypothetical protein | 0.44 | 12.31 |
| PABG_04756 | Hypothetical protein | 0.63 | 17.54 |
| PABG_05090 | Hypothetical protein | 0.66 | 32.77 |
| PABG_05343 | Hypothetical protein | 0.02 | 6.91 |
| PABG_06426 | Hypothetical protein | 0.23 | 5.99 |
| PABG_06921 | Hypothetical protein | 0.23 | 17.93 |
| PABG_11229 | Hypothetical protein | 0.64 | 6.33 |
| PABG_11333 | Hypothetical protein | 0.11 | 5.95 |
| PABG_11463 | Hypothetical protein | 0.60 | 40.98 |
| PABG_11948 | Hypothetical protein | 0.33 | 24.46 |
| PABG_12207 | Hypothetical protein | 0.60 | 21.50 |
| PABG_12415 | Hypothetical protein | 0.65 | 9.57 |
| PABG_12441 | Hypothetical protein | 0.35 | 16.88 |
| PABG_12446 | Hypothetical protein | 0.26 | 6.08 |
| PABG_12574 | Hypothetical protein | 0.60 | 16.37 |
| PABG_11804 | Hypothetical protein | 0.07 | 32.99 |
| PABG_12023 | Hypothetical protein | 0.27 | 16.18 |
| PABG_12033 | Hypothetical protein | 0.61 | 11.84 |
| PABG_07597 | Hypothetical protein | 0.60 | 26.57 |
| PABG_05701 | Hypothetical protein | 0.50 | 11.83 |
| PABG_03826 | Hypothetical protein | 0.61 | 16.57 |
| PABG_06575 | Hypothetical protein | 0.55 | 21.23 |
| PABG_03151 | Hypothetical protein | 0.56 | 28.09 |
| PABG_05579 | Hypothetical protein | 0.66 | 48.37 |
| PABG_11583 | Hypothetical protein | 0.66 | 23.08 |

^a^ Identification of differentially regulated proteins from *Paracoccidioides* genome database (http://www.broadinstitute.org/annotation/genome/paracoccidioides_brasiliensis/MultiHome.html) using the ProteinLynx Global Server vs. 2.4 (PLGS) (Waters Corporation, Manchester, UK).

^b^ Proteins annotation from *Paracoccidioides* genome database or by homology from NCBI database (<http://www.ncbi.nlm.nih.gov/>).

^c^ Acetate/Glucose means: The level of expression in yeast cells derived from cultured in sodium acetate divided by the level in the control yeast cells cultured in glucose.

^d^ Biological process of differentially expressed proteins from MIPS (http://mips.helmholtz-muenchen.de/funcatDB/) and Uniprot databases (http://www.uniprot.org/).
