# Supplementary material for: Differential Metabolism of a Two-Carbon Substrate by Members of the Paracoccidioides Genus
Source: Front Microbiol. 2017 Nov 27;8:2308. doi: 10.3389/fmicb.2017.02308 (PMC5711815; doi:10.3389/fmicb.2017.02308)
Supplement: Supplementary file 5 [file Table5.DOCX]

**Supplemental Table 5: Proteins up-regulated in** *Paracoccidioides brasiliensis* **isolate 03 after growth for 48 hours in sodium acetate as carbon source.**

| **Accession number^a^** | **Protein Description^b^** | **Acetate/Glucose Ratio^c^** | **Score** |
| --- | --- | --- | --- |
| **Functional categories^d^** | |  |  |
| **1. METABOLISM** | | | |
| **Amino acid metabolism** | | | |
| PABG_07394 | 4-hydroxyphenylpyruvate dioxygenase | 4.05 | 253.46 |
| PABG_00589 | Alanine-glyoxylate aminotransferase | 2.46 | 92.50 |
| PABG_11192 | Amino-acid acetyltransferase, mitochondrial | 1.55 | 5.50 |
| PABG_07531 | Asparagine synthetase | 1.88 | 122.47 |
| PABG_02806 | Aspartate aminotransferase | 1.96 | 91.85 |
| PABG_02012 | Choline dehydrogenase | 1.54 | 145.33 |
| PABG_04712 | Glutamate-5-semialdehyde dehydrogenase | 2.04 | 183.29 |
| PABG_03010 | Glutamine synthetase | 1.84 | 93.06 |
| PABG_04990 | Glycine cleavage T-protein | 1.92 | 23.45 |
| PABG_05965 | Histidine biosynthesis trifunctional protein | 1.63 | 17.44 |
| PABG_07392 | Homogentisate 1,2-dioxygenase | 3.97 | 198.61 |
| PABG_04732 | Isochorismatase domain containing 2b | 2.01 | 54.50 |
| PABG_01965 | Kynureninase | 2.00 | 94.33 |
| PABG_03129 | Kynurenine-oxoglutarate transaminase | 2.12 | 146.59 |
| PABG_07533 | Methionine aminopeptidase | 1.51 | 64.41 |
| PABG_07390 | Maleylacetoacetate isomerase | 2.66 | 111.96 |
| PABG_02827 | Ornithine aminotransferase | 2.62 | 242.23 |
| PABG_02447 | Pentafunctional AROM polypeptide | 3.05 | 206.59 |
| PABG_03361 | S-adenosylmethionine synthetase | 1.91 | 203.87 |
| PABG_01280 | L-threonine 3-dehydrogenase | 4.03 | 90.39 |
| PABG_00544 | TPA: putative Conserved lysine-rich protein | 1.89 | 296.22 |
|  |  |  |  |
| **Nitrogen, sulfur and selenium metabolism** | | | |
| PABG_00102 | Ureidoglycolate hydrolase | 5.42 | 17.84 |
| PABG_07028 | Formamidase | 4.45 | 240.33 |
| PABG_03480 | Nitroreductase | 1.65 | 87.22 |
| PABG_02398 | Urea carboxylase | 1.60 | 416.19 |
|  |  |  |  |
| **Nucleotide/nucleoside/nucleobase metabolism** | | | |
| PABG_04433 | Adenylosuccinate lyase | 1.58 | 95.31 |
| PABG_06340 | Inosine-5'-monophosphate dehydrogenase IMD2 | 1.69 | 316.83 |
| PABG_06368 | Quinone oxidoreductase | 2.66 | 184.61 |
| PABG_01949 | Uricase | 1.52 | 59.50 |
|  |  |  |  |
| **Phosphate metabolism** | | | |
| PABG_04460 | Serine/threonine protein kinase | 4.94 | 16.22 |
|  |  |  |  |
| **C-compound and carbohydrate metabolism** | | | |
| PABG_00225 | 4-coumarate-CoA ligase | 1.57 | 193.77 |
| PABG_01732 | Tautomerase/MIF | 4.05 | 90.54 |
| PABG_05538 | Lactonohydrolase | 1.91 | 94.83 |
| PABG_02864 | Mannitol-1-phosphate 5-dehydrogenase | 1.53 | 241.02 |
| PABG_00813 | NADPH-dependent D-xylose reductase | 1.82 | 155.84 |
| PABG_04877 | Quinone oxidoreductase | 2.06 | 174.79 |
| PABG_01695 | Retrograde regulation protein | 2.89 | 65.11 |
| PABG_01914 | Ribulokinase | 1.73 | 53.46 |
| PABG_12460 | Salicylate hydroxylase | 1.57 | 47.49 |
| PABG_06330 | 4-carboxymuconolactone decarboxylase family protein | 1.58 | 53.38 |
| PABG_06193 | 6-phosphogluconolactonase | 2.08 | 86.31 |
|  |  |  |  |
| **Lipid, fatty acid and isoprenoid metabolism** | | | |
| PABG_02112 | 2-succinylbenzoate-CoA ligase | 1.53 | 183.90 |
| PABG_00747 | 3-ketoacyl-CoA thiolase A | 1.52 | 209.23 |
| PABG_05999 | 3-ketoacyl-acyl carrier protein reductase | 3.90 | 29.01 |
| PABG_05972 | 3-oxoacyl-(acyl-carrier protein) reductase | 1.89 | 331.40 |
| PABG_01791 | Acyl-CoA dehydrogenase | 1.71 | 78.53 |
| PABG_03016 | Enoyl-CoA hydratase | 1.77 | 19.23 |
| PABG_02855 | Acyl-CoA-binding protein | 1.77 | 89.55 |
| PABG_00955 | Acyl-CoA N-acyltransferase | 3.78 | 51.19 |
|  |  |  |  |
| **Metabolism of vitamins, cofactors, and prosthetic groups** | | | |
| PABG_01387 | 6,7-dimethyl-8-ribityllumazine synthase | 1.84 | 37.06 |
| PABG_06591 | Delta-aminolevulinic acid dehydratase | 3.15 | 86.93 |
| PABG_01549 | NUDIX domain-containing protein | 27.49 | 17.47 |
| PABG_04836 | Nudix hydrolase | 1.82 | 36.29 |
| PABG_00890 | Phosphopantothenate-cysteine ligase | 2.24 | 46.28 |
|  |  |  |  |
| **Secondary metabolism** | | | |
| PABG_06389 | Imidazole glycerol phosphate synthase hisHF | 3.16 | 55.33 |
| PABG_06397 | Imidazole glycerol phosphate synthase hisHF | 3.21 | 177.12 |
|  |  |  |  |
| **2. ENERGY** | | | |
| **Glycolysis and gluconeogenesis** | | | |
| PABG_06480 | Glucokinase GLK1 | 1.68 | 42.70 |
| PABG_02052 | Glucose-6-phosphate isomerase | 2.16 | 217.75 |
| PABG_00022 | Glyceraldehyde-3-phosphate dehydrogenase | 1.56 | 363.42 |
| PABG_01237 | Hexokinase | 1.73 | 107.40 |
|  |  |  |  |
| **Ethanol production** | | | |
| PABG_04316 | Alcohol dehydrogenase GroES domain-containing protein | 2.88 | 279.02 |
| PABG_12402 | Alcohol dehydrogenase 1 | 2.66 | 212.74 |
|  |  |  |  |
| **Pentose-phosphate pathway** | | | |
| PABG_02360 | Ribose-phosphate pyrophosphokinase | 1.64 | 173.62 |
|  |  |  |  |
| **Tricarboxylic-acid pathway** | | | |
| PABG_03210 | 2-oxoglutarate dehydrogenase E1 | 1.53 | 486.63 |
| PABG_11957 | Aconitate hydratase, mitochondrial | 1.89 | 405.25 |
| PABG_04594 | ATP-citrate synthase | 4.00 | 343.90 |
| PABG_01382 | Isocitrate dehydrogenase subunit 1 | 1.69 | 216.34 |
| PABG_03494 | Pyruvate dehydrogenase E1 component subunit alpha | 1.87 | 278.19 |
| PABG_03772 | Succinate dehydrogenase flavoprotein subunit | 2.66 | 276.05 |
|  |  |  |  |
| **Methylcytrate cycle** | | | |
| PABG_04323 | Methylisocitrate lyase | 2.54 | 265.71 |
|  |  |  |  |
| **Electron transport and membrane-associated energy conservation** | | | |
| PABG_06161 | ATP synthase delta chain | 3.25 | 28.85 |
| PABG_12007 | ATP synthase F0 subunit 8 | 1.84 | 107.56 |
| PABG_11057 | ATP synthase subunit alpha, mitochondrial | 1.63 | 458.48 |
| PABG_07285 | ATP synthase subunit beta | 1.87 | 349.38 |
| PABG_06178 | ATP synthase subunit delta, | 1.97 | 59.54 |
| PABG_05268 | Cytochrome c oxidase polypeptide Vib | 1.79 | 19.26 |
| PABG_06935 | Formate dehydrogenase | 6.10 | 137.07 |
| PABG_07303 | F-type H+-transporting ATPase subunit epsilon | 1.54 | 14.76 |
| PABG_12311 | NADH-ubiquinone oxidoreductase 49 kDa, mitochondrial | 1.56 | 11.77 |
| PABG_11661 | Succinate dehydrogenase assembly factor 2, mitochondrial | 1.63 | 91.85 |
| PABG_05474 | Vacuolar ATP synthase catalytic subunit A | 1.88 | 247.75 |
| PABG_03708 | NADH-ubiquinone oxidoreductase 12 kDa subunit | 2.98 | 22.65 |
| PABG_03177 | Ubiquinol-cytochrome c reductase subunit 6 | 6.17 | 32.84 |
| PABG_06958 | 12-oxophytodienoate reductase | 3.19 | 284.78 |
| PABG_04666 | Cytochrome b5 | 1.64 | 22.16 |
| PABG_11490 | Electron transfer flavoprotein-ubiquinone oxidoreductase | 9.02 | 10.87 |
|  |  |  |  |
| **3. CELL CYCLE and DNA PROCESSING** | | | |
| PABG_05569 | Cell cycle control protein cwf14 | 2.09 | 15.92 |
| PABG_02067 | Mitochondrial genome maintenance protein MGM101 | 1.76 | 73.79 |
| PABG_04448 | Meiosis specific protein Hop1 | 2.20 | 45.43 |
| PABG_03596 | SNF7 family protein Fti1/Did2 | 3.67 | 15.15 |
| PABG_05201 | Septin-7 | 2.12 | 150.23 |
| PABG_11327 | Proliferating cell nuclear antigen | 2.03 | 284.78 |
|  |  |  |  |
| **4. TRANSCRIPTION** | | | |
| PABG_00085 | Pirin | 2.55 | 95.05 |
| PABG_06262 | PHD finger protein | 2.03 | 18.92 |
| PABG_06274 | NuA3 HAT complex component NTO1 | 4.01 | 60.61 |
| PABG_06004 | Poly(A) polymerase PAPa | 2.77 | 56.98 |
| PABG_01856 | Splicing factor spf30 | 1.77 | 28.68 |
| PABG_03761 | tRNA (guanine(37)-N1)-methyltransferase | 2.71 | 79.13 |
| PABG_03438 | Transcription factor RfeF | 1.56 | 76.06 |
| PABG_05850 | U1 small nuclear ribonucleoprotein | 1.54 | 33.37 |
| PABG_12042 | U2 small nuclear ribonucleoprotein B | 1.51 | 26.05 |
| PABG_00485 | U2 snRNP-associated protein Uap2 | 2.05 | 43.68 |
| PABG_01474 | U4/U6.U5 tri-snRNP-associated protein | 2.59 | 50.62 |
| PABG_00260 | DNA-directed RNA polymerase III subunit Rpc31 | 2.17 | 46.98 |
| PABG_05962 | Basic helix-loop-helix transcription factor Yas1p | 5.32 | 90.70 |
| PABG_01008 | Zinc finger protein | 1.78 | 59.80 |
|  |  |  |  |
| **5. PROTEIN SYNTHESIS** | | | |
| PABG_06421 | 30S ribosomal protein S7 | 1.66 | 163.76 |
| PABG_04888 | 37S ribosomal protein S5 | 4.68 | 18.45 |
| PABG_03113 | 50S ribosomal protein L24 | 1.52 | 19.86 |
| PABG_01500 | 60S ribosomal protein L11 | 2.75 | 70.92 |
| PABG_12185 | 60S ribosomal protein L28 | 1.84 | 310.74 |
| PABG_06407 | Cytochrome P450 monooxygenase | 4.99 | 17.08 |
| PABG_06666 | Mitochondrial ribosomal protein YmL8 | 1.65 | 33.50 |
| PABG_12047 | Ran-specific GTPase-activating protein 1 | 1.58 | 265.86 |
| PABG_01633 | Ribosomal protein L30 | 1.80 | 59.78 |
| PABG_06017 | TCTP family protein | 1.90 | 53.61 |
| PABG_01959 | 67 kDa polymerase-associated factor PAF67 | 1.84 | 93.92 |
| PABG_02129 | Elongation factor 1-alpha | 2.10 | 59.90 |
| PABG_05845 | Elongation factor 2 | 2.79 | 142.18 |
| PABG_01416 | Eukaryotic translation initiation factor 3 110 kDa subunit | 1.84 | 109.69 |
| PABG_06490 | Eukaryotic translation initiation factor 3 subunit B | 2.41 | 192.82 |
| PABG_01208 | Eukaryotic translation initiation factor 3 subunit I | 1.74 | 106.67 |
| PABG_02641 | Translation initiation factor 4B | 2.31 | 206.77 |
| PABG_01301 | Lysine-tRNA ligase | 1.84 | 135.04 |
| PABG_05580 | Seryl-tRNA synthetase | 2.86 | 265.30 |
| PABG_07397 | Lysyl-tRNA synthetase | 2.42 | 145.34 |
|  |  |  |  |
| **6. PROTEIN FATE** | | | |
| PABG_00967 | T-complex protein 1 subunit alpha | 2.68 | 164.60 |
| PABG_01488 | Peptidyl-prolyl cis-trans isomerase B | 1.59 | 111.53 |
| PABG_06135 | TCTP family protein | 1.59 | 106.15 |
| PABG_06572 | T-complex protein 1 subunit beta | 1.53 | 91.90 |
| PABG_07652 | FK506-binding protein | 2.33 | 77.53 |
| PABG_07742 | T-complex protein 1 subunit eta | 2.12 | 117.58 |
| PABG_01434 | Chaperone protein dnaJ | 1.58 | 78.19 |
| PABG_05205 | Intermembrane space import and assembly protein | 2.25 | 25.77 |
| PABG_00818 | Mitochondrial import inner membrane translocase subunit tim9 | 1.54 | 11.98 |
| PABG_01181 | NADH dehydrogenase (ubiquinone) 1 beta subcomplex 8 | 5.77 | 78.45 |
| PABG_00491 | Ubiquitin-like modifier SUMO | 1.53 | 25.45 |
| PABG_03958 | Subtilase-type proteinase psp3 | 1.66 | 123.42 |
| PABG_05357 | 26S proteasome regulatory subunit rpn-8 | 2.10 | 84.59 |
| PABG_05859 | Ubiquitin thiolesterase | 1.62 | 20.56 |
| PABG_06586 | T-complex protein 1 subunit beta | 5.36 | 186.67 |
| PABG_07230 | Xaa-Pro aminopeptidase I | 2.40 | 182.17 |
| PABG_07373 | Proteasome subunit alpha type-4 | 1.81 | 137.44 |
| PABG_01386 | Proteasome subunit alpha type-3 | 1.56 | 141.66 |
| PABG_05365 | V Chain V | 1.67 | 78.82 |
| PABG_06849 | Carboxypeptidase Y | 2.63 | 94.09 |
|  |  |  |  |
| **7. PROTEIN WITH BINDING FUNCTION or COFACTOR REQUIREMENT** | | | |
| PABG_03848 | YjeF-related protein | 1.68 | 53.33 |
| PABG_03778 | Nuclear localization sequence-binding protein | 3.16 | 48.38 |
| PABG_03796 | Translation machinery-associated protein 22 | 3.18 | 13.29 |
| PABG_03042 | APAF1-interacting protein | 1.52 | 50.27 |
| PABG_04194 | Progesterone binding protein | 2.69 | 82.62 |
| PABG_02026 | RNA-binding protein Vip1 | 1.62 | 152.84 |
|  |  |  |  |
| **8. CELLULAR TRANSPORT, TRANSPORT FACILITIES and TRANSPORT ROUTES** | | | |
| PABG_06877 | HATPase_c domain-containing protein | 1.57 | 215.25 |
| PABG_06615 | Heavy metal ion transporter, putative | 1.75 | 31.52 |
| PABG_00598 | Galactose-proton symport | 1.54 | 22.94 |
| PABG_01896 | GTP-binding protein SAS1 | 3.52 | 40.46 |
| PABG_03783 | Exocyst complex component Sec10 | 2.59 | 102.59 |
| PABG_12318 | DUF726 domain-containing protein | 1.67 | 41.63 |
|  |  |  |  |
| **9. CELLULAR COMMUNICATION/SIGNAL TRANSDUCTION MECHANISM** | | | |
| PABG_06817 | Neuronal calcium sensor 1 | 1.72 | 16.47 |
| PABG_03846 | WD repeat-containing protein | 7.39 | 38.28 |
| PABG_00188 | Phosphatase 2A regulatory B subunit | 1.87 | 50.26 |
| PABG_01056 | Kinase binding protein | 1.75 | 33.99 |
| PABG_05936 | CAMK/CAMKL/KIN4 protein kinase | 5.77 | 78.45 |
| PABG_04460 | Serine/threonine protein kinase | 4.94 | 16.22 |
|  |  |  |  |
| **10. CELL RESCUE, DEFENSE AND VIRULENCE** | | | |
| **Stress response** | | | |
| PABG_01369 | Heat shock protein | 2.86 | 235.05 |
| PABG_02373 | Heat shock protein | 1.77 | 243.44 |
| PABG_00374 | Heat shock protein Hsp88 | 1.71 | 643.84 |
| PABG_00350 | Heat shock protein SSB | 1.75 | 325.75 |
| PABG_05342 | Hsp70-like protein | 1.56 | 747.87 |
|  |  |  |  |
| **Detoxification** | | | |
| PABG_03954 | Superoxide dismutase Cu/Zn SOD1 | 2.16 | 72.78 |
| PABG_03204 | Superoxide dismutase Fe/Mn SOD2 | 2.52 | 147.87 |
| PABG_00431 | Superoxide dismutase Cu/Zn SOD3 | 1.58 | 43.93 |
| PABG_03387 | Superoxide dismutase Fe/Mn SOD5 | 1.76 | 168.45 |
| PABG_01943 | Peroxisomal catalase | 7.86 | 400.41 |
| PABG_04219 | Glutathione peroxidase | 3.61 | 32.22 |
| PABG_00949 | Glutathione S-transferase Gst3 | 1.63 | 209.16 |
| PABG_06156 | Protein disulfide-isomerase | 2.27 | 174.38 |
| PABG_05451 | Serine/threonine protein phosphatase 2A | 2.81 | 18.58 |
| PABG_00720 | Cytochrome c peroxidase | 3.20 | 257.66 |
|  |  |  |  |
| **11. BIOGENESIS OF CELLULAR COMPONENTS** | | | |
| **Cell wall** | | | |
| PABG_07783 | Antigenic cell wall protein | 2.55 | 44.49 |
| PABG_06590 | Glucan 1,3-beta-glucosidase | 3.38 | 31.35 |
| PABG_04069 | Neutral alpha-glucosidase AB | 1.56 | 224.70 |
|  |  |  |  |
| **Cytoskeleton/strucutural proteins** | | | |
| PABG_01263 | Actin | 2.02 | 97.21 |
| PABG_07733 | Actin lateral binding protein | 1.66 | 183.02 |
| PABG_03455 | Clathrin light chain | 2.17 | 101.18 |
|  |  |  |  |
| **12. MISCELLANEOUS** | | | |
| PABG_01011 | Urg3 | 1.58 | 90.54 |
| PABG_01127 | Short chain dehydrogenase/reductase | 1.86 | 37.43 |
| PABG_06951 | Polysaccharide deacetylase family protein | 3.96 | 185.15 |
| PABG_01628 | Adenosine deaminase | 1.58 | 24.48 |
| PABG_06653 | CUE domain-containing protein | 2.40 | 11.68 |
| PABG_02064 | DUF833 domain-containing protein | 1.60 | 98.02 |
| PABG_11267 | Alkaline phosphatase | 3.09 | 79.08 |
| PABG_05421 | Glyoxylate reductase | 1.50 | 31.50 |
| PABG_04660 | Propionate-CoA ligase | 1.56 | 35.02 |
| PABG_03731 | Peroxisomal dehydratase | 1.52 | 39.28 |
| PABG_06197 | S-formylglutathione hydrolase | 3.11 | 19.17 |
| PABG_02094 | Globin | 3.36 | 51.68 |
|  |  |  |  |
| **13. UNCLASSIFIED** | | | |
| PABG_01748 | Hypothetical protein | 2.89 | 38.06 |
| PABG_00361 | Hypothetical protein | 46.44 | 34.74 |
| PABG_00446 | Hypothetical protein | 1.64 | 26.97 |
| PABG_05798 | Hypothetical protein | 1.89 | 48.03 |
| PABG_00679 | Hypothetical protein | 1.52 | 17.78 |
| PABG_01780 | Hypothetical protein | 1.94 | 18.43 |
| PABG_01814 | Hypothetical protein | 2.18 | 30.82 |
| PABG_01892 | Hypothetical protein | 2.51 | 4.89 |
| PABG_02578 | Hypothetical protein | 4.23 | 298.74 |
| PABG_03540 | Hypothetical protein | 4.58 | 11.31 |
| PABG_03999 | Hypothetical protein | 2.17 | 16.92 |
| PABG_04137 | Hypothetical protein | 2.06 | 43.70 |
| PABG_05041 | Hypothetical protein | 1.83 | 50.89 |
| PABG_05293 | Hypothetical protein | 5.54 | 5.76 |
| PABG_05692 | Hypothetical protein | 2.81 | 6.16 |
| PABG_07502 | Hypothetical protein | 3.16 | 10.93 |
| PABG_11028 | Hypothetical protein | 2.16 | 28.49 |
| PABG_11204 | Hypothetical protein | 2.18 | 44.75 |
| PABG_11386 | Hypothetical protein | 5.86 | 10.38 |
| PABG_11594 | Hypothetical protein | 9.51 | 10.87 |
| PABG_11755 | Hypothetical protein | 2.89 | 22.06 |
| PABG_12078 | Hypothetical protein | 1.53 | 5.39 |
| PABG_12239 | Hypothetical protein | 105.53 | 10.55 |
| PABG_12338 | Hypothetical protein | 4.75 | 6.03 |
| PABG_12351 | Hypothetical protein | 11.89 | 12.24 |
| PABG_12386 | Hypothetical protein | 1.81 | 18.19 |
| PABG_12408 | Hypothetical protein | 1.58 | 5.77 |
| PABG_12656 | Hypothetical protein | 2.53 | 6.17 |
| PABG_03766 | Hypothetical protein | 1.53 | 59.76 |
| PABG_06624 | Hypothetical protein | 1.57 | 40.31 |
| PABG_01741 | Hypothetical protein | 2.05 | 108.18 |
| PABG_01683 | Hypothetical protein | 7.30 | 34.10 |
| PABG_02970 | Hypothetical protein | 2.65 | 104.93 |
| PABG_06275 | Hypothetical protein | 1.55 | 76.58 |

^a^ Identification of differentially regulated proteins from *Paracoccidioides* genome database (http://www.broadinstitute.org/annotation/genome/paracoccidioides_brasiliensis/MultiHome.html) using the ProteinLynx Global Server vs. 2.4 (PLGS) (Waters Corporation, Manchester, UK).

^b^ Proteins annotation from *Paracoccidioides* genome database or by homology from NCBI database (<http://www.ncbi.nlm.nih.gov/>).

^c^ Acetate/Glucose means: The level of expression in yeast cells derived from cultured in sodium acetate divided by the level in the control yeast cells cultured in glucose.

^d^ Biological process of differentially expressed proteins from MIPS (http://mips.helmholtz-muenchen.de/funcatDB/) and Uniprot databases (http://www.uniprot.org/).
