# Supplementary material for: Differential Metabolism of a Two-Carbon Substrate by Members of the Paracoccidioides Genus
Source: Front Microbiol. 2017 Nov 27;8:2308. doi: 10.3389/fmicb.2017.02308 (PMC5711815; doi:10.3389/fmicb.2017.02308)
Supplement: Supplementary file 6 [file Table6.DOCX]

**Supplemental Table 6: Proteins down-regulated in** *Paracoccidioides brasiliensis* **isolate 339 after growth for 48 hours in sodium acetate as carbon source.**

| **Accession number^a^** | **Protein Description^b^** | **Acetate/Glucose Ratio^c^** | **Score** |
| --- | --- | --- | --- |
| **Functional categories^d^** | |  |  |
| **1- METABOLISM** | | | |
| **Amino acid metabolism** | | | |
| PADG_01536 | Glutamine synthetase | 0.006 | 189.43 |
| PADG_01305 | Argininosuccinate lyase | 0.56 | 138.41 |
| PADG_04356 | Cystathionine gamma-synthase | 0.56 | 47.23 |
| PADG_05888 | Aspartokinase | 0.36 | 52.77 |
| PADG_06955 | Tryptophan synthase | 0.49 | 45.67 |
| PADG_00210 | Glycine dehydrogenase | 0.37 | 364.02 |
| PADG_00402 | 5-carboxymethyl-2-hydroxymuconate semialdehyde | 0.31 | 41.71 |
| PADG_07845 | Imidazole glycerol phosphate synthase hisHF | 0.64 | 65.71 |
| PADG_07269 | Phenylacetate 2-hydroxylase | 0.54 | 37.80 |
| PADG_05058 | Chorismate mutase | 0.34 | 18.79 |
| PADG_01789 | Anthranilate phosphoribosyltransferase | 0.56 | 33.26 |
| PADG_00386 | Phospho-2-dehydro-3-deoxyheptonate aldolase | 0.63 | 102.63 |
| PADG_03167 | 3-isopropylmalate dehydratase | 0.26 | 242.64 |
| PADG_06671 | 3-isopropylmalate dehydrogenase A | 0.57 | 142.25 |
| PADG_08225 | Acetolactate synthase small subunit | 0.47 | 37.94 |
| PADG_01993 | Acetoacetyl-coenzyme A synthetase | 0.18 | 140.90 |
| PADG_03032 | Ornithine decarboxylase | 0.25 | 26.09 |
| PADG_01404 | Aspartate aminotransferase | 0.33 | 177.33 |
| PADG_02726 | Cysteine synthase | 0.57 | 160.19 |
| PADG_04570 | Branched-chain-amino-acid aminotransferase | 0.45 | 247.14 |
| PADG_08662 | Cystathionine beta-lyase | 0.36 | 43.57 |
| PADG_04522 | Homoserine kinase | 0.64 | 62.14 |
| PADG_07241 | Dihydroxy-acid dehydratase | 0.38 | 32.19 |
|  |  |  |  |
| **Nitrogen, sulfur and selenium metabolism** | | | |
| PADG_07471 | 2-nitropropane dioxygenase | 0.24 | 49.64 |
| PADG_08696 | GPR/FUN34 family protein | 0.65 | 24.05 |
|  |  |  |  |
| **Nucleotide/nucleoside/nucleobase metabolism** | | | |
| PADG_07918 | Pyrimidine and pyridine-specific 5'-nucleotidase | 0.33 | 18.78 |
| PADG_02246 | Adenosine kinase | 0.47 | 76.39 |
| PADG_04721 | Amidophosphoribosyltransferase | 0.51 | 65.16 |
| PADG_06585 | GMP synthase | 0.62 | 149.68 |
| PADG_12229 | Phosphoribosylaminoimidazole carboxylase, ATPase subunit | 0.44 | 15.42 |
| PADG_07585 | Inosine-5'-monophosphate dehydrogenase IMD2 | 0.30 | 199.70 |
| PADG_06747 | DNA-directed RNA polymerase II subunit RPB1 | 0.23 | 134.66 |
| PADG_05472 | GMP synthase | 0.58 | 88.64 |
| PADG_01424 | Inosine-uridine preferring nucleoside hydrolase | 0.39 | 37.33 |
| PADG_08530 | Thymidylate synthase | 0.17 | 24.38 |
| PADG_08434 | Nicotinamide mononucleotide adenylyltransferase | 0.66 | 44.15 |
| PADG_00209 | Guanine deaminase | 0.36 | 47.55 |
| PADG_04293 | Adenine phosphoribosyltransferase | 0.36 | 28.72 |
| PADG_05812 | CTP synthase | 0.62 | 26.72 |
| PADG_11922 | Uridine kinase | 0.45 | 27.11 |
| PADG_05643 | Ribonucleotide reductase R2 subunit variant | 0.33 | 32.31 |
| PADG_00988 | Ribonuclease T2 | 0.13 | 18.40 |
| PADG_07888 | Eukaryotic translation initiation factor 5A | 0.60 | 61.25 |
|  |  |  |  |
| **Compound and carbohydrate metabolism** | | | |
| PADG_00315 | Carbonic anhydrase | 0.35 | 22.16 |
| PADG_01636 | 4-hydroxyphenylpyruvate dioxygenase | 0.38 | 43.44 |
| PADG_02625 | 4-coumarate-CoA ligase | 0.30 | 157.12 |
| PADG_03943 | Phosphomannomutase | 0.47 | 77.85 |
| PADG_08474 | Mannose-1-phosphate guanyltransferase | 0.27 | 137.39 |
| PADG_04761 | Glucosidase I | 0.66 | 119.08 |
| PADG_04900 | Alpha,alpha-trehalose-phosphate synthase | 0.42 | 68.93 |
| PADG_02145 | Glycogen phosphorylase | 0.37 | 210.39 |
| PADG_08244 | Neutral trehalase | 0.34 | 58.59 |
| PADG_05778 | Glycogen synthase | 0.23 | 30.08 |
| PADG_12009 | Trehalose 6-phosphate synthase | 0.65 | 21.34 |
| PADG_05419 | UDP-glucose 4-epimerase | 0.67 | 27.75 |
| PADG_00561 | Galactokinase | 0.56 | 97.74 |
| PADG_06878 | Nuclear protein SNF4 | 0.01 | 6.06 |
| PADG_07319 | Sporulation-regulated protein | 0.52 | 176.16 |
| PADG_04754 | General amidase | 0.13 | 22.46 |
| PADG_03984 | Glucosamine-fructose-6-phosphateaminotransferase | 0.19 | 187.73 |
|  |  |  |  |
| **Lipid, fatty acid and isoprenoid metabolism** | | | |
| PADG_02244 | (R)-benzylsuccinyl-CoA dehydrogenase | 0.34 | 113.46 |
| PADG_04827 | Acyl-coenzyme A synthetase O-MACS | 0.64 | 28.94 |
| PADG_08214 | Diphosphomevalonate decarboxylase | 0.62 | 11.51 |
| PADG_01291 | Enoyl-CoA hydratase | 0.50 | 67.28 |
| PADG_05184 | Ethanolamine-phosphate cytidylyltransferase | 0.42 | 60.36 |
| PADG_00255 | Fatty acid synthase subunit beta dehydratase | 0.29 | 779.10 |
| PADG_02174 | Geranylgeranyl pyrophosphate synthetase | 0.30 | 80.02 |
| PADG_00685 | Hydroxymethylglutaryl-CoA synthase | 0.61 | 91.51 |
| PADG_01786 | NMDA receptor-regulated protein | 0.45 | 65.05 |
| PADG_00221 | Short-chain dehydrogenase | 0.01 | 18.54 |
| PADG_00204 | Sterol 24-C-methyltransferase | 0.54 | 16.68 |
| PADG_00244 | Trans-2-enoyl-CoA reductase | 0.51 | 148.67 |
| PADG_05733 | 60 kDa lysophospholipase | 0.53 | 32.35 |
| PADG_01677 | Acetyl-coenzyme A synthetase | 0.55 | 444.46 |
|  |  |  |  |
| **Metabolism of vitamins, cofactors, and prosthetic groups** | | | |
| PADG_04250 | C-1-tetrahydrofolate synthase | 0.41 | 204.60 |
| PADG_07528 | Cysteine desulfurase | 0.52 | 84.11 |
| PADG_12043 | Folylpolyglutamate synthase | 0.66 | 216.61 |
| PADG_01126 | Molybdopterin synthase large subunit CnxH | 0.60 | 33.05 |
| PADG_05564 | Porphobilinogen deaminase | 0.07 | 17.82 |
| PADG_00607 | Riboflavin synthase alpha chain | 0.60 | 105.09 |
| PADG_11535 | Folylpolyglutamate synthase | 0.50 | 76.60 |
|  |  |  |  |
| **2- ENERGY** | | | |
| **Glycolysis and gluconeogenesis** | | | |
| PADG_00192 | 6-phosphofructokinase | 0.65 | 228.35 |
| PADG_01896 | Phosphoglycerate kinase | 0.29 | 440.10 |
|  |  |  |  |
| **Pentose-phosphate pathway** | | | |
| PADG_07771 | 6-phosphogluconolactonase | 0.31 | 84.60 |
| PADG_04989 | Ribose 5-phosphate isomerase A | 0.50 | 66.11 |
| PADG_11295 | Ribose-phosphate pyrophosphokinase 3 | 0.13 | 14.25 |
| PADG_11296 | Ribose-phosphate pyrophosphokinase 3 | 0.35 | 107.68 |
|  |  |  |  |
| **Tricarboxylic-acid pathway** | | | |
| PADG_00317 | Succinyl-CoA ligase subunit beta | 0.30 | 241.32 |
| PADG_02260 | Succinyl-CoA ligase subunit alpha | 0.41 | 172.36 |
|  |  |  |  |
| **Electron transport and membrane-associated energy conservation** | | | |
| PADG_00688 | F-type H+-transporting ATPase subunit h | 0.55 | 12.42 |
| PADG_04729 | ATP synthase D chain, mitochondrial | 0.57 | 97.65 |
| PADG_06978 | Cytochrome c | 0.22 | 46.71 |
| PADG_01841 | Cytochrome c oxidase assembly protein COX19 | 0.35 | 11.31 |
| PADG_06995 | Cytochrome c oxidase subunit 6b | 0.59 | 22.32 |
| PADG_08460 | NADPH dehydrogenase | 0.16 | 27.45 |
| PADG_11605 | NADPH-cytochrome P450 reductase | 0.51 | 18.52 |
| PADG_06956 | Vacuolar ATP synthase subunit B | 0.66 | 192.96 |
| PADG_12152 | ATP synthase subunit g | 0.48 | 42.19 |
|  |  |  |  |
| **3- CELL CYCLE AND DNA PROCESSING** | | | |
| PADG_00656 | Non-histone chromosomal protein 6 | 0.32 | 72.14 |
| PADG_00466 | Mitochondrial genome maintenance protein | 0.59 | 43.94 |
| PADG_07835 | Replication factor-A protein | 0.58 | 73.25 |
| PADG_05798 | Single-strand binding protein family | 0.34 | 108.62 |
| PADG_05900 | Transcription elongation factor 1 | 0.05 | 6.16 |
| PADG_02359 | Cell division control protein | 0.14 | 175.26 |
| PADG_05714 | Cell division control protein | 0.42 | 127.64 |
| PADG_05683 | Cell division control protein | 0.59 | 533.91 |
| PADG_07477 | Ankyrin repeat protein | 0.51 | 22.46 |
| PADG_11711 | ATP-dependent RNA helicase eIF4A | 0.09 | 148.50 |
| PADG_08634 | Bud emergence protein | 0.22 | 45.52 |
| PADG_08425 | CBS domain-containing protein | 0.45 | 5.61 |
| PADG_07070 | Cell division control protein | 0.23 | 111.87 |
| PADG_11337 | Chromodomain helicase hrp3 | 0.43 | 42.40 |
| PADG_03005 | Dynein light chain | 0.06 | 27.84 |
| PADG_05837 | Glycoprotein FP21 | 0.42 | 71.95 |
| PADG_11743 | Meiotic recombination protein DMC1 | 0.04 | 18.98 |
| PADG_00951 | Microtubule-associated protein RP/EB family | 0.08 | 17.08 |
| PADG_05875 | Centromere/microtubule-binding protein CBF5 | 0.61 | 28.47 |
| PADG_11857 | Mitotic checkpoint protein BUB3 | 0.31 | 97.97 |
| PADG_05893 | Nucleosome assembly protein | 0.44 | 222.87 |
| PADG_08676 | RNA binding effector protein Scp160 | 0.46 | 80.06 |
| PADG_00128 | Tubulin alpha chain | 0.15 | 139.30 |
| PADG_08316 | Tubulin alpha-1 chain | 0.36 | 45.78 |
|  |  |  |  |
| **4-TRANSCRIPTION** | | | |
| PADG_08081 | DNA-binding protein HEXBP | 0.50 | 97.05 |
| PADG_00718 | histone chaperone asf1 | 0.33 | 19.61 |
| PADG_01455 | KH domain RNA-binding protein | 0.49 | 39.05 |
| PADG_04307 | mRNA binding post-transcriptional regulator | 0.44 | 107.67 |
| PADG_04730 | Nascent polypeptide-associated complex subunit alpha | 0.48 | 82.97 |
| PADG_04657 | Nascent polypeptide-associated complex subunit beta | 0.34 | 89.53 |
| PADG_06931 | Nitrogen regulatory protein DAL80 | 0.09 | 16.37 |
| PADG_00067 | Polymerase (RNA) II (DNA directed) polypeptide D | 0.55 | 55.09 |
| PADG_06666 | RNA splicing factor Pad-1 | 0.46 | 107.95 |
| PADG_01232 | TFIIH basal transcription factor complex p52 subunit | 0.60 | 11.53 |
| PADG_00034 | Transcription elongation factor S-II | 0.52 | 27.66 |
| PADG_03406 | Transcription initiation factor IIA small subunit | 0.49 | 10.47 |
| PADG_06752 | Transcription initiation factor TFIID subunit 9 | 0.29 | 6.02 |
| PADG_02825 | Splicing factor | 0.03 | 7.00 |
| PADG_03988 | LSM domain-containing protein | 0.04 | 5.79 |
| PADG_00205 | Pre-mRNA-splicing factor slt11 | 0.43 | 23.81 |
| PADG_04107 | Pre-mRNA-splicing factor srp1 | 0.23 | 39.87 |
| PADG_01999 | Small nuclear ribonucleoprotein Sm D3 | 0.48 | 23.87 |
| PADG_02752 | 116 kDa U5 small nuclear ribonucleoprotein component | 0.64 | 175.86 |
| PADG_11945 | Dicer-like protein 1 | 0.55 | 17.75 |
| PADG_05468 | U2 small nuclear ribonucleoprotein A | 0.35 | 41.01 |
| PADG_05210 | tRNA pseudouridine synthase | 0.13 | 6.27 |
|  |  |  |  |
| **5- PROTEIN SYNTHESIS** | | | |
| PADG_00785 | Ribosomal protein S15 | 0.48 | 40.22 |
| PADG_01026 | 60S ribosomal protein L43 | 0.63 | 51.64 |
| PADG_04118 | 60S ribosomal protein L38 | 0.67 | 71.05 |
| PADG_04588 | 60S ribosomal protein L22 | 0.60 | 108.74 |
| PADG_04862 | 50S ribosomal protein Mrp49 | 0.14 | 41.45 |
| PADG_00333 | 40S ribosomal protein S16 | 0.40 | 118.42 |
| PADG_00354 | 40S ribosomal protein S17 | 0.60 | 106.93 |
| PADG_01083 | 60S ribosomal protein L32 | 0.53 | 87.21 |
| PADG_06048 | 40S ribosomal protein S27 | 0.63 | 43.46 |
| PADG_01914 | Ribosomal protein L35 | 0.58 | 40.50 |
| PADG_02056 | 50S ribosomal protein L12 | 0.12 | 77.69 |
| PADG_02797 | 54S ribosomal protein L3 | 0.39 | 23.68 |
| PADG_04030 | 60S acidic ribosomal protein P0 | 0.42 | 87.86 |
| PADG_04475 | Small subunit ribosomal protein YMR-31 | 0.08 | 25.98 |
| PADG_05244 | 60S ribosomal protein L44 | 0.42 | 91.24 |
| PADG_05573 | 54S ribosomal protein L19 | 0.02 | 12.21 |
| PADG_06266 | 40S ribosomal protein S9 | 0.51 | 60.26 |
| PADG_06502 | 40S ribosomal protein S20 | 0.66 | 60.72 |
| PADG_06599 | 40S ribosomal protein S25 | 0.35 | 128.23 |
| PADG_00044 | 28 kDa ribonucleoprotein | 0.54 | 72.82 |
| PADG_07583 | Ribosomal protein S21E | 0.41 | 94.11 |
| PADG_07685 | 40S ribosomal protein S13-1 | 0.49 | 138.04 |
| PADG_08152 | 40S ribosomal protein S26E | 0.53 | 74.30 |
| PADG_08453 | Large subunit ribosomal protein L6 | 0.47 | 37.26 |
| PADG_02691 | Eukaryotic translation initiation factor 6 | 0.64 | 68.59 |
| PADG_04948 | H/ACA ribonucleoprotein complex subunit 1 | 0.31 | 28.80 |
| PADG_05561 | Serine/threonine-protein kinase RIO2 | 0.58 | 24.50 |
| PADG_06315 | Ribosome maturation protein SDO1 | 0.30 | 32.79 |
| PADG_04810 | GTP-binding nuclear protein GSP1/Ran | 0.27 | 195.81 |
| PADG_11083 | Ribosomal protein L13 | 0.23 | 25.38 |
| PADG_12373 | 40S ribosomal protein S30 | 0.25 | 14.06 |
| PADG_02206 | DnaJ homolog subfamily B member 4 | 0.19 | 72.99 |
| PADG_05118 | Translation initiation factor 3 subunit K | 0.52 | 46.32 |
| PADG_04672 | ATP-dependent RNA helicase SUB2 | 0.05 | 167.39 |
| PADG_00692 | Elongation factor 1-alpha | 0.36 | 358.93 |
| PADG_02896 | Elongation factor 1-beta | 0.56 | 52.70 |
| PADG_08125 | Elongation factor 2 | 0.50 | 693.37 |
| PADG_01949 | Elongation factor Tu | 0.56 | 288.95 |
| PADG_01891 | translation initiation factor RLI1 | 0.46 | 137.06 |
| PADG_05879 | Eukaryotic peptide chain release factor subunit 1 | 0.39 | 89.77 |
| PADG_07977 | Eukaryotic translation initiation factor 1A | 0.62 | 74.56 |
| PADG_04210 | Eukaryotic translation initiation factor 2 beta subunit | 0.31 | 51.86 |
| PADG_06160 | Eukaryotic translation initiation factor 2 subunit alpha | 0.13 | 25.23 |
| PADG_04083 | Eukaryotic translation initiation factor 2 subunit gamma | 0.55 | 172.47 |
| PADG_01389 | Eukaryotic translation initiation factor 3 subunit 8 | 0.37 | 151.50 |
| PADG_02296 | Eukaryotic translation initiation factor 3 subunit F | 0.56 | 77.19 |
| PADG_00438 | Eukaryotic translation initiation factor 3 subunit G | 0.36 | 24.30 |
| PADG_11111 | Nuclear transport factor 2 | 0.27 | 36.71 |
| PADG_02588 | Protein phosphatase PP2A regulatory subunit B | 0.43 | 55.01 |
| PADG_00041 | Ran GTPase-activating protein | 0.42 | 149.41 |
| PADG_06110 | Translation factor SUI1 | 0.20 | 49.36 |
| PADG_01079 | Translation initiation factor 4B | 0.59 | 106.95 |
| PADG_00457 | Translation initiation factor 4G | 0.47 | 194.73 |
| PADG_00080 | Translation initiation factor eIF3 | 0.58 | 98.81 |
| PADG_11904 | Translational activator GCN1 | 0.46 | 250.07 |
| PADG_05164 | Transport protein SEC13 | 0.36 | 11.01 |
| PADG_11157 | tRNA (cytosine-5-)-methyltransferase NCL1 | 0.61 | 71.74 |
| PADG_07962 | MADS box transcription factor Mcm1 | 0.12 | 25.06 |
| PADG_04962 | Aspartyl-tRNA synthetase | 0.42 | 58.09 |
| PADG_02340 | Methionyl-tRNA synthetase | 0.27 | 102.74 |
| PADG_04116 | Methionyl-tRNA synthetase | 0.39 | 39.58 |
| PADG_04949 | Threonyl-tRNA synthetase | 0.62 | 134.68 |
| PADG_03642 | Tryptophanyl-tRNA synthetase | 0.40 | 23.39 |
| PADG_03689 | Tyrosyl-tRNA synthetase | 0.51 | 111.10 |
|  |  |  |  |
| **6- PROTEIN FATE** | | | |
| PADG_01432 | Peptidyl-prolyl cis-trans isomerase NIMA-interacting 4 | 0.66 | 13.47 |
| PADG_05011 | Peptidyl-prolyl cis-trans isomerase | 0.60 | 16.83 |
| PADG_08049 | Peptidyl-prolyl cis-trans isomerase cyp15 | 0.53 | 18.35 |
| PADG_05124 | Prefoldin subunit 3 | 0.10 | 5.68 |
| PADG_04034 | Mitochondrial protein import protein MAS5 | 0.29 | 156.98 |
| PADG_08340 | Ubiquitin fusion degradation protein | 0.59 | 43.97 |
| PADG_07804 | Transport protein sec23 | 0.35 | 157.52 |
| PADG_04883 | Transport protein SEC24 | 0.26 | 73.58 |
| PADG_07515 | UBX domain-containing protein | 0.48 | 54.37 |
| PADG_08048 | T-complex protein 1 subunit beta | 0.45 | 164.83 |
| PADG_01114 | Importin subunit alpha-1a | 0.20 | 57.31 |
| PADG_05884 | Mitochondrial import inner membrane translocase TIM8 | 0.51 | 29.77 |
| PADG_05740 | SNARE domain-containing protein | 0.18 | 11.15 |
| PADG_06439 | NEDD8-conjugating enzyme Ubc12 | 0.60 | 24.02 |
| PADG_07156 | Histone acetyltransferase type B catalytic subunit | 0.44 | 97.29 |
| PADG_04880 | Mitogen-activated protein kinase | 0.41 | 18.33 |
| PADG_02197 | DNA damage tolerance protein rad31 | 0.36 | 10.74 |
| PADG_07925 | Ubiquitin-conjugating enzyme | 0.30 | 22.05 |
| PADG_01992 | Mitochondrial-processing peptidase subunit alpha | 0.37 | 29.13 |
| PADG_11423 | A-factor-processing enzyme | 0.57 | 50.03 |
| PADG_06359 | Bleomycin hydrolase | 0.46 | 68.67 |
| PADG_05335 | Iron sulfur cluster assembly protein | 0.57 | 49.04 |
| PADG_06655 | Coatomer subunit delta | 0.17 | 58.08 |
| PADG_00300 | 26S protease regulatory subunit 8 | 0.26 | 130.16 |
| PADG_12469 | 26S proteasome non-ATPase regulatory subunit 6 | 0.55 | 115.87 |
| PADG_04234 | 26S proteasome non-ATPase regulatory subunit 8 | 0.51 | 39.33 |
| PADG_00071 | 26S proteasome regulatory subunit RPN10 | 0.62 | 23.37 |
| PADG_08095 | 26S proteasome regulatory subunit rpn-8 | 0.21 | 61.66 |
| PADG_03944; | 26S proteasome non-ATPase regulatory subunit 10 | 0.40 | 104.76 |
| PADG_06276 | O-sialoglycoprotein endopeptidase | 0.22 | 11.99 |
| PADG_07929 | Ubiquitin carboxyl-terminal hydrolase | 0.53 | 119.33 |
| PADG_11319 | Ubiquitin-conjugating enzyme variant MMS2 | 0.49 | 97.58 |
| PADG_00634 | Vacuolar protease A | 0.56 | 64.16 |
| PADG_06815 | Xaa-Pro dipeptidase | 0.47 | 50.79 |
|  |  |  |  |
| **7- PROTEIN WITH BINDING FUNCTION OR COFACTOR REQUIREMENT** | | | |
| PADG_03895 | Dynein light chain LC8-type | 0.33 | 18.65 |
| PADG_00011 | Actin binding protein | 0.53 | 76.87 |
| PADG_03390 | Molybdenum cofactor synthesis protein cinnamon | 0.57 | 15.55 |
| PADG_01032 | Curved DNA-binding protein | 0.46 | 178.28 |
| PADG_05381 | RNA-binding protein | 0.23 | 10.31 |
| PADG_02022 | Clathrin light chain | 0.66 | 57.40 |
| PADG_01849 | GTP-dependent nucleic acid-binding protein engD | 0.60 | 225.76 |
| PADG_02207 | GTP-binding protein | 0.17 | 37.00 |
| PADG_08304 | GTP-binding protein YPTM2 | 0.18 | 126.10 |
| PADG_04420 | Peptide methionine sulfoxide reductase msrA | 0.41 | 59.54 |
| PADG_05308 | Type 2A phosphatase activator tip41 | 0.17 | 75.35 |
| PADG_11347 | TIA1 cytotoxic granule-associated RNA binding protein | 0.37 | 59.17 |
|  |  |  |  |
| **8- CELLULAR TRANSPORT, TRANSPORT FACILITIES AND TRANSPORT ROUTES** | | | |
| PADG_07957 | Ferric reductase transmembrane component 7 | 0.64 | 34.72 |
| PADG_00622 | Arsenical pump-driving ATPase | 0.35 | 60.57 |
| PADG_04852 | EH domain binding protein epsin 2 | 0.63 | 23.32 |
| PADG_00282 | GTP-binding protein SAS1 | 0.14 | 40.32 |
| PADG_02686 | Reduced viability upon starvation protein | 0.56 | 28.49 |
| PADG_00442 | Arf gtpase-activating protein | 0.38 | 48.44 |
| PADG_03551 | F-actin-capping protein subunit alpha | 0.31 | 107.29 |
| PADG_00937 | Ras-related protein Rab-5C | 0.12 | 119.27 |
| PADG_02833 | ADP-ribosylation factor | 0.41 | 113.72 |
| PADG_07930 | ARP2/3 complex 20 kDa subunit | 0.46 | 75.83 |
| PADG_02401 | ARP2/3 complex 21 kDa subunit | 0.53 | 39.72 |
| PADG_07014 | Vesicular-fusion protein sec17 | 0.60 | 39.59 |
|  |  |  |  |
| **9- CELLULAR COMMUNICATION/SIGNAL TRANSDUCTION MECHANISM** | | | |
| PADG_02300 | Protein phosphatase 2C | 0.66 | 95.94 |
| PADG_02153 | Mitogen-activated protein kinase HOG1 | 0.33 | 20.37 |
| PADG_11275 | CMGC/MAPK protein kinase | 0.23 | 115.16 |
| PADG_11742 | MAP Kinase Interacting Kinase | 0.43 | 26.77 |
| PADG_02800 | Adenylyl cyclase-associated protein | 0.40 | 22.60 |
| PADG_02017 | Calmodulin | 0.53 | 78.01 |
|  |  |  |  |
| **10- CELL RESCUE, DEFENSE AND VIRULENCE** | | | |
| **Stress response** | | | |
| PADG_04379 | Heat shock protein STI1 | 0.39 | 430.59 |
| PADG_02030 | Hsp90 co-chaperone Cdc37 | 0.49 | 215.55 |
| PADG_07715 | Heat shock protein | 0.13 | 913.35 |
|  |  |  |  |
| **Detoxification** | | | |
| PADG_03163 | Cytochrome c peroxidase | 0.42 | 190.52 |
| PADG_05628 | Disulfide-isomerase tigA | 0.66 | 136.92 |
| PADG_03500 | Glutamate-cysteine ligase | 0.44 | 24.59 |
| PADG_02218 | Glutathione synthetase | 0.35 | 6.79 |
| PADG_00529 | Glutaredoxin | 0.22 | 43.50 |
| PADG_03161 | Thioredoxin | 0.44 | 47.29 |
| PADG_05504 | Thioredoxin | 0.29 | 64.81 |
| PADG_05344 | Peroxiredoxin Q/BCP | 0.41 | 72.62 |
| PADG_01263 | Superoxide dismutase Fe/Mn SOD6 | 0.06 | 6.04 |
|  |  |  |  |
| **11-BIOGENESIS OF CELLULAR COMPONENTS** | | | |
| **Cell wall** | | | |
| PADG_08120 | glucosamine 6-phosphate N-acetyltransferase | 0.52 | 50.16 |
|  |  |  |  |
| **Cytoskeleton/structural proteins** | | | |
| PADG_07249 | Actin binding protein | 0.50 | 213.57 |
| PADG_12076 | Actin | 0.12 | 130.34 |
| PADG_12077 | Actin | 0.05 | 277.77 |
| PADG_12426 | 1,4-alpha-glucan-branching enzyme | 0.57 | 149.20 |
| PADG_02125 | Actin | 0.34 | 61.89 |
| PADG_00945 | Actin related protein 2/3 complex, subunit 5 | 0.47 | 26.96 |
| PADG_05538 | Actin | 0.18 | 196.88 |
|  |  |  |  |
| **12- MISCELLANEOUS** | | | |
| PADG_04260 | HET-C protein | 0.66 | 59.08 |
| PADG_05341 | Fimbrin | 0.12 | 136.68 |
| PADG_00967 | Golgi complex component Cog3 | 0.20 | 66.05 |
| PADG_03226 | P450 monooxygenase | 0.18 | 41.23 |
| PADG_03397; | DNA-directed RNA polymerase | 0.54 | 34.40 |
| PADG_03288 | ATP NAD kinase | 0.32 | 15.25 |
| PADG_08290 | KOW motif domain-containing protein | 0.50 | 43.87 |
| PADG_12310 | Histidinol dehydrogenase | 0.34 | 24.25 |
| PADG_12196 | Nucleolar protein 58 | 0.10 | 50.34 |
| PADG_11353 | Signal recognition particle subunit SRP72 | 0.51 | 133.11 |
| PADG_11302 | VosA | 0.54 | 43.80 |
| PADG_02846 | Glutaredoxin domain-containing protein | 0.50 | 32.28 |
| PADG_02664 | SAP domain-containing protein | 0.66 | 14.73 |
| PADG_03121 | DUF757 domain-containing protein | 0.39 | 5.75 |
| PADG_00694 | Ankyrin repeat protein | 0.63 | 29.01 |
| PADG_01160 | PCI domain-containing protein | 0.13 | 75.47 |
| PADG_03244 | HD domain-containing protein | 0.45 | 46.86 |
| PADG_03382 | DUF866 domain-containing protein | 0.66 | 18.76 |
| PADG_08034 | Dienelactone hydrolase family protein | 0.50 | 44.15 |
| PADG_12437 | EF hand domain-containing protein | 0.42 | 167.04 |
| PADG_00999 | CAP20 protein | 0.17 | 12.03 |
| PADG_03830 | WD repeat-containing protein 1-B | 0.64 | 274.62 |
|  |  |  |  |
| **13-UNCLASSIFIED** | | | |
| PADG_08451 | Hypothetical protein | 0.20 | 34.33 |
| PADG_04879 | Hypothetical protein | 0.06 | 17.15 |
| PADG_00237 | Hypothetical protein | 0.28 | 56.24 |
| PADG_05340 | Hypothetical protein | 0.60 | 146.99 |
| PADG_08212 | Hypothetical protein | 0.52 | 24.19 |
| PADG_12237 | Hypothetical protein | 0.27 | 4.84 |
| PADG_01021 | Hypothetical protein | 0.10 | 45.59 |
| PADG_04869 | Hypothetical protein | 0.44 | 69.49 |
| PADG_03654 | Hypothetical protein | 0.48 | 32.32 |
| PADG_07452 | Hypothetical protein | 0.27 | 22.59 |
| PADG_00046 | Hypothetical protein | 0.11 | 18.66 |
| PADG_00222 | Hypothetical protein | 0.18 | 87.21 |
| PADG_00388 | Hypothetical protein | 0.35 | 24.52 |
| PADG_00440 | Hypothetical protein | 0.30 | 28.00 |
| PADG_07979 | Hypothetical protein | 0.58 | 70.27 |
| PADG_00541 | Hypothetical protein | 0.52 | 33.49 |
| PADG_00555 | Hypothetical protein | 0.50 | 16.23 |
| PADG_00769 | Hypothetical protein | 0.07 | 14.58 |
| PADG_00921 | Hypothetical protein | 0.08 | 27.52 |
| PADG_00944 | Hypothetical protein | 0.45 | 66.92 |
| PADG_01148 | Hypothetical protein | 0.28 | 6.29 |
| PADG_01285 | Hypothetical protein | 0.50 | 36.81 |
| PADG_01287 | Hypothetical protein | 0.63 | 48.47 |
| PADG_05491 | Hypothetical protein | 0.28 | 37.75 |
| PADG_01409 | Hypothetical protein | 0.44 | 6.58 |
| PADG_02092 | Hypothetical protein | 0.22 | 49.52 |
| PADG_02307 | Hypothetical protein | 0.31 | 44.23 |
| PADG_02346 | Hypothetical protein | 0.62 | 6.37 |
| PADG_02671 | Hypothetical protein | 0.11 | 5.02 |
| PADG_02759 | Hypothetical protein | 0.14 | 91.01 |
| PADG_03110 | Hypothetical protein | 0.34 | 9.25 |
| PADG_03135 | Hypothetical protein | 0.04 | 6.28 |
| PADG_03159 | Hypothetical protein | 0.24 | 5.67 |
| PADG_03428 | Hypothetical protein | 0.43 | 26.01 |
| PADG_03612 | Hypothetical protein | 0.48 | 16.32 |
| PADG_03631 | Hypothetical protein | 0.06 | 44.77 |
| PADG_03645 | Hypothetical protein | 0.13 | 11.83 |
| PADG_03660 | Hypothetical protein | 0.58 | 131.28 |
| PADG_03827 | Hypothetical protein | 0.38 | 23.83 |
| PADG_04057 | Hypothetical protein | 0.21 | 116.34 |
| PADG_05921 | Hypothetical protein | 0.11 | 17.62 |
| PADG_04357 | Hypothetical protein | 0.36 | 72.76 |
| PADG_04423 | Hypothetical protein | 0.66 | 21.64 |
| PADG_04494 | Hypothetical protein | 0.55 | 58.26 |
| PADG_04512 | Hypothetical protein | 0.66 | 17.30 |
| PADG_05274 | Hypothetical protein | 0.02 | 15.43 |
| PADG_05408 | Hypothetical protein | 0.31 | 21.89 |
| PADG_05462 | Hypothetical protein | 0.30 | 83.91 |
| PADG_05935 | Hypothetical protein | 0.39 | 44.40 |
| PADG_06220 | Hypothetical protein | 0.14 | 17.50 |
| PADG_06435 | Hypothetical protein | 0.11 | 22.82 |
| PADG_06473 | Hypothetical protein | 0.27 | 18.61 |
| PADG_06690 | Hypothetical protein | 0.55 | 81.80 |
| PADG_06798 | Hypothetical protein | 0.11 | 10.65 |
| PADG_07205 | Hypothetical protein | 0.44 | 6.83 |
| PADG_07264 | Hypothetical protein | 0.63 | 15.30 |
| PADG_02475 | Hypothetical protein | 0.16 | 36.57 |
| PADG_07414 | Hypothetical protein | 0.61 | 29.26 |
| PADG_07520 | Hypothetical protein | 0.62 | 40.32 |
| PADG_07935 | Hypothetical protein | 0.01 | 6.06 |
| PADG_08037 | Hypothetical protein | 0.27 | 24.34 |
| PADG_08116 | Hypothetical protein | 0.39 | 44.75 |
| PADG_08238 | Hypothetical protein | 0.15 | 26.79 |
| PADG_08305 | Hypothetical protein | 0.50 | 19.26 |
| PADG_08342 | Hypothetical protein | 0.47 | 73.54 |
| PADG_08368 | Hypothetical protein | 0.18 | 156.18 |
| PADG_08413 | Hypothetical protein | 0.29 | 42.13 |
| PADG_08480 | Hypothetical protein | 0.64 | 72.47 |
| PADG_08483 | Hypothetical protein | 0.63 | 39.18 |
| PADG_08693 | Hypothetical protein | 0.28 | 41.79 |
| PADG_11091 | Hypothetical protein | 0.04 | 6.89 |
| PADG_11366 | Hypothetical protein | 0.17 | 27.08 |
| PADG_11406 | Hypothetical protein | 0.13 | 5.15 |
| PADG_11572 | Hypothetical protein | 0.62 | 5.85 |
| PADG_11593 | Hypothetical protein | 0.01 | 5.68 |
| PADG_11622 | Hypothetical protein | 0.02 | 5.13 |
| PADG_11992 | Hypothetical protein | 0.37 | 4.73 |
| PADG_12058 | Hypothetical protein | 0.11 | 5.46 |
| PADG_12194 | Hypothetical protein | 0.46 | 16.62 |
| PADG_05205 | Hypothetical protein | 0.46 | 6.04 |
| PADG_00735 | Hypothetical protein | 0.19 | 107.46 |
| PADG_12364 | Hypothetical protein | 0.32 | 6.42 |

^a^ Identification of differentially regulated proteins from *Paracoccidioides* genome database (http://www.broadinstitute.org/annotation/genome/paracoccidioides_brasiliensis/MultiHome.html) using the ProteinLynx Global Server vs. 2.4 (PLGS) (Waters Corporation, Manchester, UK).

^b^ Proteins annotation from *Paracoccidioides* genome database or by homology from NCBI database (<http://www.ncbi.nlm.nih.gov/>).

^c^ Acetate/Glucose means: The level of expression in yeast cells derived from cultured in sodium acetate divided by the level in the control yeast cells cultured in glucose.

^d^ Biological process of differentially expressed proteins from MIPS (http://mips.helmholtz-muenchen.de/funcatDB/) and Uniprot databases (http://www.uniprot.org/).
