# Supplementary material for: Differential Metabolism of a Two-Carbon Substrate by Members of the Paracoccidioides Genus
Source: Front Microbiol. 2017 Nov 27;8:2308. doi: 10.3389/fmicb.2017.02308 (PMC5711815; doi:10.3389/fmicb.2017.02308)
Supplement: Supplementary file 7 [file Table7.DOCX]

**Supplemental Table 7: Proteins up-regulated in** *Paracoccidioides brasiliensis* **isolate 339 after growth for 48 hours in sodium acetate as carbon source.**

| **Accession number^a^** | **Protein Description^b^** | **Acetate/Glucose Ratio^c^** | **Score** |
| --- | --- | --- | --- |
| **Functional categories^d^** | |  |  |
| **1- METABOLISM** | | | |
| **Amino acid metabolism** | | | |
| PADG_04686 | Glutamine synthetase | 8.93 | 59.90 |
| PADG_05085 | Delta-1-pyrroline-5-carboxylate dehydrogenase | 1.86 | 297.72 |
| PADG_04419 | Carbapenem antibiotics biosynthesis protein carD | 1.77 | 47.21 |
| PADG_05337 | Gamma-glutamyl phosphate reductase | 6.95 | 207.31 |
| PADG_03825 | NAD-specific glutamate dehydrogenase | 2.47 | 760.59 |
| PADG_00663 | Homoserine dehydrogenase | 3.83 | 164.03 |
| PADG_02456 | Cystathionine gamma-lyase | 2.18 | 67.15 |
| PADG_11705 | L-serine dehydratase | 3.02 | 70.21 |
| PADG_06213 | Phosphoserine aminotransferase | 1.59 | 188.76 |
| PADG_05896 | D-3-phosphoglycerate dehydrogenase | 1.51 | 69.27 |
| PADG_06301 | Imidazoleglycerol-phosphate dehydratase | 1.60 | 45.04 |
| PADG_01286 | Homoisocitrate dehydrogenase | 1.87 | 80.02 |
| PADG_08092 | L-aminoadipate-semialdehyde dehydrogenase large subunit | 1.98 | 211.00 |
| PADG_08452 | 3',5'-bisphosphate nucleotidase | 2.63 | 47.97 |
| PADG_07369 | Isovaleryl-CoA dehydrogenase | 1.63 | 258.19 |
| PADG_00637 | Arginase | 2.88 | 59.92 |
| PADG_07029 | Acetylornithine aminotransferase | 1.80 | 95.62 |
| PADG_04516 | NADP-specific glutamate dehydrogenase | 2.65 | 163.29 |
| PADG_08176 | Cobalamin-independent synthase | 2.79 | 78.70 |
| PADG_01886 | Adenosylhomocysteinase | 1.91 | 236.81 |
| PADG_03058 | Succinate-semialdehyde dehydrogenase | 2.33 | 240.51 |
| PADG_01228 | 3-hydroxybutyryl-CoA dehydrogenase | 1.87 | 141.99 |
| PADG_07274 | Anthranilate synthase component 2 | 2.13 | 283.23 |
| PADG_07366 | Methylcrotonoyl-CoA carboxylase subunit alpha | 1.92 | 139.22 |
| PADG_02214 | 4-aminobutyrate aminotransferase | 1.62 | 231.77 |
| PADG_00066 | tRNA methyltransferase Trm5 | 2.05 | 93.01 |
| PADG_01314 | YggS family pyridoxal phosphate enzyme | 5.78 | 98.63 |
| PADG_03671 | Phenylpyruvate tautomerase | 2.03 | 141.35 |
| PADG_03964 | Fumarylacetoacetate hydrolase domain-containing protein | 2.27 | 119.23 |
| PADG_04142 | Fumarylacetoacetate hydrolase domain-containing protein | 2.81 | 50.39 |
|  |  |  |  |
| **Nitrogen, sulfur and selenium metabolism** | | | |
| PADG_01697 | Carbonic anhydrase | 2.23 | 44.05 |
| PADG_07674 | Carbonic anhydrase | 2.40 | 182.02 |
| PADG_06490 | Formamidase | 1.68 | 237.65 |
| PADG_08009 | Monoxygenase | 1.53 | 45.63 |
| PADG_00734 | Urea carboxylase | 5.50 | 185.29 |
| PADG_02728 | Sulfite oxidase | 1.67 | 153.46 |
| PADG_02048 | Nitroreductase family protein | 1.55 | 84.01 |
|  |  |  |  |
| **Nucleotide/nucleoside/nucleobase metabolism** | | | |
| PADG_08098 | Adenylate kinase cytosolic | 4.65 | 93.05 |
| PADG_02183 | ADP-ribose pyrophosphatase | 6.15 | 77.89 |
| PADG_04340 | DNA-directed RNA polymerase II subunit RPB2 | 2.78 | 188.52 |
| PADG_06897 | mRNA turnover protein | 5.23 | 12.90 |
| PADG_08066 | Purine nucleoside phosphorylase | 1.71 | 28.86 |
| PADG_12503 | RNA-directed DNA polymerase | 2.84 | 83.25 |
| PADG_01100 | Uracil phosphoribosyltransferase | 2.72 | 38.01 |
| PADG_06197 | Xanthine dehydrogenase | 3.77 | 207.86 |
|  |  |  |  |
| **Phosphate metabolism** | | | |
| PADG_01617 | Phosphotransmitter protein Ypd1 | 1.89 | 15.33 |
| PADG_06273 | Calcineurin subunit B | 1.69 | 18.99 |
|  |  |  |  |
| **C-compound and carbohydrate metabolism** | | | |
| PADG_01372 | Mannitol-1-phosphate 5-dehydrogenase | 1.52 | 267.33 |
| PADG_03278 | Myo-inositol-1-phosphate synthase | 1.74 | 134.21 |
| PADG_04687 | 3-beta-hydroxysteroid dehydrogenase | 1.61 | 64.42 |
| PADG_04939 | Succinyl-CoA:3-ketoacid-coenzyme A transferase subunit B | 1.52 | 175.46 |
| PADG_06765 | NADP-dependent leukotriene B4 12-hydroxydehydrogenase | 4.51 | 81.09 |
| PADG_03268 | NADPH-dependent D-xylose reductase | 1.89 | 169.53 |
| PADG_07618 | Vanillin dehydrogenase | 2.27 | 111.12 |
| PADG_08722 | Glycogen synthase kinase 3 beta | 1.68 | 123.02 |
| PADG_02847 | HpcH/HpaI aldolase | 2.49 | 101.30 |
|  |  |  |  |
| **Lipid, fatty acid and isoprenoid metabolism** | | | |
| PADG_00513 | 2-succinylbenzoate-CoA ligase | 2.07 | 164.96 |
| PADG_06876 | 3-hydroxyisobutyryl-CoA hydrolase | 3.15 | 187.30 |
| PADG_01687 | 3-ketoacyl-CoA thiolase | 1.71 | 307.68 |
| PADG_04495 | 4-coumarate-CoA ligase | 2.18 | 94.45 |
| PADG_08468 | 4-hydroxyphenylpyruvate dioxygenase | 1.69 | 234.68 |
| PADG_02751 | Acetyl-CoA acetyltransferase/ tiolase | 1.51 | 195.73 |
| PADG_02597 | Acetyl-CoA hydrolase | 1.59 | 17.87 |
| PADG_02991 | Acyl-coenzyme A oxidase/ desidrogenase | 1.56 | 194.48 |
| PADG_07023 | Carnitine O-acetyltransferase | 9.11 | 220.24 |
| PADG_05783 | Farnesyl pyrophosphate synthetase | 1.56 | 128.79 |
| PADG_00254 | Fatty acid synthase subunit alpha reductase | 4.09 | 511.16 |
| PADG_00608 | Formyl-coenzyme A transferase | 1.66 | 148.41 |
| PADG_07031 | Hydroxymethylglutaryl-CoA lyase | 1.92 | 134.59 |
| PADG_05281 | Propionate-CoA ligase | 2.05 | 21.27 |
| PADG_07699 | S-formylglutathione hydrolase | 1.57 | 69.52 |
| PADG_01486 | Short chain dehydrogenase/reductase family | 2.17 | 122.05 |
|  |  |  |  |
| **Metabolism of vitamins, cofactors, and prosthetic groups** | | | |
| PADG_08108 | Coproporphyrinogen III oxidase | 3.52 | 78.55 |
| PADG_05490 | Molybdopterin binding domain-containing protein | 1.76 | 89.22 |
| PADG_05947 | Nicotinate-nucleotide pyrophosphorylase | 1.53 | 211.13 |
| PADG_06088 | Ubiquinone biosynthesis protein coq-4 | 1.77 | 47.39 |
| PADG_04032 | Uroporphyrinogen decarboxylase | 1.79 | 127.64 |
|  |  |  |  |
| **Secondary metabolism** | | | |
| PADG_02981 | ThiJ/PfpI family protein | 5.21 | 207.87 |
|  |  |  |  |
| **ENERGY** | | | |
| **Glycolysis and gluconeogenesis** | | | |
| PADG_05109 | 2,3-bisphosphoglycerate-independent phosphoglycerate mutase | 1.75 | 179.69 |
| PADG_05951 | Aldose 1-epimerase | 1.52 | 35.78 |
| PADG_01706 | Fructose-1,6-bisphosphatase | 1.93 | 123.19 |
| PADG_02411 | Glyceraldehyde-3-phosphate dehydrogenase | 2.25 | 404.23 |
| PADG_11132 | Phosphoglucomutase | 1.85 | 521.06 |
|  |  |  |  |
| **Ethanol production** | | | |
| PADG_04701 | Alcohol dehydrogenase | 4.63 | 246.56 |
| PADG_11405 | Alcohol dehydrogenase 1 | 1.89 | 209.72 |
|  |  |  |  |
| **Pentose-phosphate pathway** | | | |
| PADG_04604 | Transketolase | 1.88 | 362.84 |
| PADG_07420 | Transaldolase | 1.52 | 339.23 |
| PADG_00780 | Ribose-phosphate pyrophosphokinase | 2.86 | 169.19 |
| PADG_07217 | Ribose-phosphate pyrophosphokinase | 2.01 | 39.11 |
| PADG_11977 | Ribokinase | 2.05 | 57.87 |
|  |  |  |  |
| **Tricarboxylic-acid pathway** | | | |
| PADG_01762 | 2-oxoglutarate dehydrogenase E1 | 1.65 | 419.90 |
| PADG_04993 | ATP-citrate synthase subunit 1 | 2.02 | 287.66 |
| PADG_01546 | Citrate lyase subunit beta | 2.77 | 92.07 |
| PADG_08119 | Fumarate hydratase | 3.25 | 180.84 |
| PADG_12250 | Pyruvate dehydrogenase kinase | 3.65 | 59.22 |
| PADG_07213 | Pyruvate dehydrogenase protein X component | 1.83 | 254.39 |
|  |  |  |  |
|  |  |  |  |
| **Methylcytrate cycle** | | | |
| PADG_04710 | 2-methylcitrate synthase | 2.32 | 421.12 |
| PADG_04709 | Mitochondrial 2-methylisocitrate lyase | 2.98 | 225.72 |
|  |  |  |  |
| **Electron transport and membrane-associated energy conservation** | | | |
| PADG_05750 | Cytochrome c oxidase subunit Via | 1.88 | 35.10 |
| PADG_07813 | ATP synthase gamma chain | 1.91 | 130.08 |
| PADG_02561 | ATPase alpha subunit | 1.93 | 446.71 |
| PADG_00171 | Cytochrome b2 | 2.91 | 217.80 |
| PADG_06221 | Formate dehydrogenase | 3.88 | 167.37 |
| PADG_04175 | Inorganic pyrophosphatase | 1.69 | 221.03 |
| PADG_05343 | NADH-ubiquinone oxidoreductase 21.3 kDa subunit | 1.60 | 16.70 |
| PADG_06201 | Oxidoreductase ucpA | 1.64 | 69.11 |
| PADG_05436 | Ubiquinol-cytochrome c reductase iron-sulfur subunit | 1.73 | 45.49 |
| PADG_04501 | Ubiquinol-cytochrome c reductase subunit 7 | 5.84 | 31.65 |
| PADG_04319 | V-type ATPase, G subunit | 4.30 | 162.12 |
| PADG_01519 | NADPH dehydrogenase | 3.94 | 134.21 |
| PADG_06196 | 12-oxophytodienoate reductase | 3.03 | 393.26 |
|  |  |  |  |
| **3- CELL CYCLE AND DNA PROCESSING** | | | |
| PADG_01391 | DNA repair and recombination protein RAD26 | 1.57 | 114.81 |
| PADG_06911 | DNA repair and recombination protein pif1 | 1.87 | 18.51 |
| PADG_08606 | DNA mismatch repair protein Msh3 | 2.44 | 71.26 |
| PADG_04056 | 14-3-3 protein epsilon | 1.71 | 265.12 |
| PADG_02724 | Asparagine-rich protein | 1.72 | 56.47 |
| PADG_05369 | Cell division control protein | 1.54 | 26.77 |
| PADG_02157 | Cytoskeletal adaptor protein SagA | 2.00 | 119.38 |
| PADG_05906 | Histone H2a | 6.64 | 85.50 |
| PADG_05907 | Histone H2B type 1-A | 1.84 | 100.20 |
| PADG_11679 | Proliferating cell nuclear antigen | 1.65 | 254.32 |
| PADG_04004 | Serine/threonine-protein kinase Chk2 | 76.71 | 27.88 |
| PADG_06182 | Transcriptional repressor TUP1 | 1.98 | 204.90 |
| PADG_02900 | Tubulin beta chain | 1.73 | 44.21 |
|  |  |  |  |
| **4- TRANSCRIPTION** | | | |
| PADG_07629 | C2H2 finger domain-containing protein | 4.27 | 54.51 |
| PADG_00873 | Histone H3 | 2.55 | 76.34 |
| PADG_00872 | Histone H4 | 3.48 | 127.89 |
| PADG_07134 | Histone H4.2 | 3.33 | 152.75 |
| PADG_04910 | NGG1-interacting factor 3 | 1.80 | 22.23 |
| PADG_04966 | Phosducin family protein | 1.73 | 36.21 |
| PADG_02473 | Pirin | 3.49 | 21.15 |
| PADG_07509 | Transcription initiation factor TFIID subunit 6 | 1.92 | 20.86 |
| PADG_08345 | Transcriptional regulator | 2.27 | 32.84 |
| PADG_05885 | Cell cycle control protein cwf14 | 1.98 | 35.57 |
| PADG_03696 | Nuclear polyadenylated RNA-binding protein Nab2 | 2.87 | 26.71 |
| PADG_02996 | PAB1 binding protein | 2.71 | 70.85 |
| PADG_05545 | Pre-mRNA-splicing factor ATP-dependent RNA helicase | 3.33 | 105.36 |
| PADG_04796 | Pre-mRNA-splicing factor rse1 | 3.62 | 196.23 |
| PADG_01783 | Splicing factor 3a subunit 2 | 4.76 | 30.86 |
| PADG_05587 | U2 small nuclear ribonucleoprotein B | 1.53 | 70.06 |
| PADG_11352 | Transcription factor RfeF | 1.98 | 30.54 |
| PADG_03431 | G4 quadruplex nucleic acid binding protein | 2.34 | 120.50 |
|  |  |  |  |
| **5- PROTEIN SYNTHESIS** | | | |
| PADG_12324 | 40S ribosomal protein S19 | 2.05 | 170.31 |
| PADG_06680 | 40S ribosomal protein S22 | 2.20 | 130.72 |
| PADG_03315 | 40S ribosomal protein S4 | 1.58 | 253.27 |
| PADG_07803 | 60S ribosomal protein L12 | 1.56 | 151.54 |
| PADG_05939 | 60S ribosomal protein L27a | 1.54 | 121.40 |
| PADG_03781 | 60S ribosomal protein L30 | 2.66 | 63.90 |
| PADG_11585 | 60S ribosomal protein L37 | 2.58 | 50.69 |
| PADG_11379 | 60S ribosomal protein L5 | 1.85 | 256.30 |
| PADG_02888 | 60S ribosomal protein L6 | 1.67 | 238.98 |
| PADG_01568 | Mitochondrial 54S ribosomal protein YmL36 | 90.96 | 11.32 |
| PADG_02064 | NAM9+ protein | 2.74 | 48.36 |
| PADG_06768 | rRNA 2'-O-methyltransferase fibrillarin | 1.96 | 69.01 |
| PADG_05787 | ATP-dependent RNA helicase FAL1 | 2.76 | 87.65 |
| PADG_06265 | Elongation factor 1-gamma 1 | 1.93 | 284.67 |
| PADG_04016 | Eukaryotic translation initiation factor 3 subunit A | 3.35 | 164.70 |
| PADG_08033 | Eukaryotic translation initiation factor 3 subunit B | 1.98 | 176.04 |
| PADG_00626 | Eukaryotic translation initiation factor 3 subunit E | 5.04 | 97.58 |
| PADG_01865 | Eukaryotic translation initiation factor 3 subunit H | 3.60 | 82.97 |
| PADG_06681 | Importin subunit beta-1 | 1.55 | 44.67 |
| PADG_06997 | Nuclear cap-binding protein | 1.52 | 23.87 |
| PADG_05199 | Nuclear cap-binding protein subunit 2 | 2.90 | 4.89 |
| PADG_02339 | Nucleoporin-17 | 2.75 | 58.96 |
| PADG_02908 | Ubiquitin-like modifier SUMO | 2.43 | 20.75 |
| PADG_04863 | Leucyl-tRNA synthetase | 2.54 | 190.09 |
| PADG_07732 | Aspartyl-tRNA synthetase | 4.75 | 31.38 |
| PADG_08472 | Lysyl-tRNA synthetase | 3.98 | 221.46 |
|  |  |  |  |
| **6- PROTEIN FATE** | | | |
| PADG_00501 | DnaJ domain-containing protein | 2.06 | 109.44 |
| PADG_00928 | T-complex protein 1 subunit gamma | 1.67 | 141.38 |
| PADG_01565 | Calreticulin | 3.92 | 91.58 |
| PADG_05129 | UDP-glucose:glycoprotein glucosyltransferase | 3.13 | 105.89 |
| PADG_06998 | Transport protein SEC31 | 2.57 | 188.97 |
| PADG_04795 | Deubiquitination-protection protein dph1 | 2.27 | 119.91 |
| PADG_05032 | Hsp90 binding co-chaperone (Sba1) | 1.81 | 55.05 |
| PADG_05094 | T-complex protein 1 subunit zeta | 1.58 | 142.62 |
| PADG_08484 | T-complex protein 1 subunit epsilon | 4.35 | 126.45 |
| PADG_08587 | FK506-binding protein | 1.60 | 33.10 |
| PADG_00590 | Cyclin-K | 4.28 | 23.20 |
| PADG_03040 | GTP-binding protein ypt5 | 4.92 | 93.94 |
| PADG_07064 | Intermembrane space import and assembly protein | 1.87 | 41.46 |
| PADG_00741 | Peroxisomal targeting signal 2 receptor | 2.46 | 15.21 |
| PADG_00910 | Serine/threonine-protein kinase ksg1 | 1.59 | 21.74 |
| PADG_02997 | Kinase family protein | 1.61 | 73.19 |
| PADG_07558 | Ubiquitin carboxyl-terminal hydrolase | 3.43 | 148.30 |
| PADG_12186 | Ankyrin repeat protein | 4.66 | 54.84 |
| PADG_06766 | Mitochondrial-processing peptidase subunit beta | 2.75 | 189.90 |
| PADG_08328 | ATP-dependent Clp protease ATP-binding subunit clpX | 1.72 | 479.84 |
| PADG_03221 | Saccharolysin | 1.80 | 232.98 |
| PADG_03290 | Tripeptidyl-peptidase | 3.13 | 124.82 |
| PADG_04167 | Aspartyl aminopeptidase | 2.24 | 236.72 |
| PADG_05193 | Xaa-Pro aminopeptidase | 1.87 | 175.50 |
| PADG_06051 | 26S proteasome regulatory subunit rpn5 | 4.69 | 96.71 |
| PADG_02636 | 26S protease regulatory subunit 4 | 1.72 | 55.81 |
| PADG_03735 | Prolyl peptidase | 1.56 | 60.12 |
| PADG_04877 | 26S proteasome non-ATPase regulatory subunit 13 | 1.69 | 115.17 |
| PADG_04076 | Proteasome component C11 | 1.60 | 98.31 |
| PADG_05160 | Dipeptidyl-peptidase | 1.70 | 278.49 |
| PADG_12323 | peptidyl-prolyl cis-trans isomerase | 3.51 | 133.30 |
| PADG_08442 | Proteasome component Y13 | 2.44 | 151.73 |
| PADG_00051 | 26S protease regulatory subunit 8 | 8.27 | 134.43 |
| PADG_02735 | Proteasome component PRE6 | 4.59 | 136.67 |
| PADG_03982 | Proteasome component C1 | 1.51 | 120.68 |
| PADG_05560 | 26S proteasome regulatory subunit rpn-1 | 2.05 | 122.81 |
| PADG_04451 | COP9 signalosome complex subunit 5 | 2.53 | 46.07 |
| PADG_11128 | 26S protease regulatory subunit 6B | 1.86 | 119.51 |
| PADG_01652 | E3 ubiquitin-protein ligase HUWE1 | 1.88 | 183.85 |
| PADG_06314 | Carboxypeptidase Y | 2.44 | 102.34 |
| PADG_07460 | Vacuolar aminopeptidase | 2.26 | 163.45 |
|  |  |  |  |
| **7-PROTEIN WITH BINDING FUNCTION OR COFACTOR REQUIREMENT** | | | |
| PADG_00352 | SH3 domain-containing protein | 1.56 | 45.03 |
| PADG_02134 | coatomer subunit epsilon | 1.62 | 57.89 |
| PADG_03098 | DUF858 domain-containing protein | 2.36 | 16.46 |
| PADG_07317 | RNA binding domain-containing protein | 2.34 | 38.76 |
| PADG_03459 | Replication factor-A protein | 1.96 | 42.03 |
| PADG_11421 | Histone H2A.Z | 3.96 | 107.71 |
| PADG_01566 | APAF1-interacting protein | 1.97 | 73.96 |
| PADG_04559 | Progesterone binding protein | 3.70 | 86.55 |
| PADG_06294 | Hsp70 nucleotide exchange factor fes1 | 1.97 | 34.23 |
| PADG_00554 | Rho GTPase | 4.37 | 21.28 |
|  |  |  |  |
| **8-CELLULAR TRANSPORT, TRANSPORT FACILITIES AND TRANSPORT ROUTES** | | | |
| PADG_07508 | CRAL/TRIO domain-containing protein | 4.35 | 78.39 |
| PADG_08401 | Phosphatidylinositol-phosphatidylcholine transfer protein | 4.40 | 28.31 |
| PADG_02640 | ATP-binding cassette sub-family F member 2 | 2.80 | 88.18 |
| PADG_04302 | Trafficking protein particle complex subunit 3 | 1.92 | 33.27 |
| PADG_06033 | NIPSNAP family protein | 2.63 | 229.81 |
| PADG_08423 | Vacuolar protein sorting-associated protein | 1.80 | 75.40 |
|  |  |  |  |
| **9-CELLULAR COMMUNICATION/SIGNAL TRANSDUCTION MECHANISM** | | | |
| PADG_01243 | Rab GDP-dissociation inhibitor | 1.78 | 309.06 |
| PADG_01787 | 1-phosphatidylinositol phosphodiesterase | 2.06 | 59.81 |
| PADG_02845 | Integrin beta-1-binding protein | 1.70 | 92.25 |
| PADG_06778 | TGF-beta signaling pathway | 4.25 | 57.42 |
| PADG_06103 | GTPase activating protein | 4.38 | 72.15 |
| PADG_11474 | HAL protein kinase | 1.91 | 42.79 |
| PADG_05608 | GTP-binding protein ypt7 | 2.73 | 17.23 |
|  |  |  |  |
| **10-CELL RESCUE, DEFENSE AND VIRULENCE** | | | |
| **Stress response** | | | |
| PADG_00778 | Hsp70 | 1.77 | 149.35 |
| PADG_02895 | Heat shock protein | 2.26 | 155.71 |
| PADG_03562 | Hsp70-like protein | 1.62 | 482.62 |
| PADG_05139 | Heat shock 70 kd protein cognate 1 | 2.15 | 99.91 |
| PADG_02785 | Heat shock protein Hsp88 | 2.24 | 683.66 |
|  |  |  |  |
| **Disease, virulence and defense** | | | |
| PADG_01479 | Gamma-glutamyltranspeptidase | 1.51 | 184.59 |
|  |  |  |  |
| **Detoxification** | | | |
| PADG_01954 | Superoxide dismutase Fe/Mn SOD5 | 1.73 | 125.51 |
| PADG_01755 | Superoxide dismutase Fe/Mn SOD2 | 2.24 | 99.33 |
| PADG_07418 | Superoxide dismutase Cu/Zn SOD1 | 2.27 | 114.85 |
| PADG_01400 | Superoxide dismutase Cu/Zn SOD4 | 1.80 | 61.42 |
| PADG_01551 | Thioredoxin reductase | 1.69 | 81.39 |
| PADG_00324 | Peroxisomal catalase | 3.57 | 356.41 |
| PADG_04587 | Peroxiredoxin HYR1 | 1.89 | 73.03 |
|  |  |  |  |
| **11. BIOGENESIS OF CELLULAR COMPONENTS** | | | |
| **Cell wall** | | | |
| PADG_07913 | Chitin synthase | 4.81 | 40.27 |
| PADG_03872 | Chitin synthase | 1.76 | 58.96 |
|  |  |  |  |
| **12- MISCELLANEOUS** | | | |
| PADG_06763 | Septum formation protein Maf | 2.09 | 27.03 |
| PADG_01499 | ISWI chromatin-remodeling complex ATPase ISW1 | 1.94 | 73.30 |
| PADG_02565 | FAS1 domain-containing protein | 2.49 | 27.57 |
| PADG_00111 | PKHD-type hydroxylase TPA1 | 3.47 | 73.21 |
| PADG_00422 | Actin cytoskeleton protein (VIP1) | 2.56 | 140.32 |
| PADG_03031 | CobW domain-containing protein | 78.49 | 121.17 |
| PADG_03436 | 3' exoribonuclease family protein | 5.74 | 12.63 |
| PADG_03526 | M protein repeat protein | 2.90 | 183.53 |
| PADG_03534 | lipase/esterase family protein | 8.49 | 49.12 |
| PADG_04223 | 2-dehydropantoate 2-reductase | 2.65 | 38.96 |
| PADG_04477 | mediator of RNA polymerase II transcription subunit 18 | 2.12 | 34.05 |
| PADG_06202 | Arp2/3 complex subunit Arc16 | 4.42 | 133.09 |
| PADG_07469 | RNase III domain-containing protein | 1.64 | 81.95 |
| PADG_07627 | 4-carboxymuconolactone decarboxylase family protein | 2.09 | 49.25 |
| PADG_11060 | viral A-type inclusion protein repeat protein | 1.60 | 112.06 |
| PADG_11652 | Poly(rC)-binding protein | 1.92 | 229.44 |
| PADG_11131 | oligoribonuclease | 1.91 | 15.84 |
| PADG_05356 | isochorismatase domain-containing protein | 5.65 | 119.19 |
| PADG_11752 | ATP-binding cassette sub-family G member 4 | 1.65 | 24.04 |
| PADG_12274 | YjeF family domain-containing protein | 2.18 | 58.70 |
| PADG_03959 | ARP2/3 actin-organizing complex subunit Sop2 | 2.99 | 177.81 |
| PADG_12252 | Phosphotransferase enzyme family protein | 1.84 | 27.91 |
| PADG_03276 | S-(hydroxymethyl)glutathione dehydrogenase | 2.57 | 132.79 |
| PADG_06906 | Triosephosphate isomerase | 2.20 | 203.21 |
|  |  |  |  |
| **13-UNCLASSIFIED** | | | |
| PADG_00085 | Hypothetical protein | 2.81 | 144.28 |
| PADG_00452 | Hypothetical protein | 3.74 | 22.84 |
| PADG_00472 | Hypothetical protein | 5.43 | 39.06 |
| PADG_00496 | Hypothetical protein | 10.27 | 64.83 |
| PADG_01009 | Hypothetical protein | 1.67 | 17.58 |
| PADG_01010 | Hypothetical protein | 1.59 | 223.24 |
| PADG_01130 | Hypothetical protein | 10.05 | 13.74 |
| PADG_01204 | Hypothetical protein | 1.84 | 24.73 |
| PADG_01488 | Hypothetical protein | 1.53 | 121.99 |
| PADG_01639 | Hypothetical protein | 1.58 | 18.75 |
| PADG_01818 | Hypothetical protein | 4.15 | 13.10 |
| PADG_01867 | Hypothetical protein | 1.54 | 93.10 |
| PADG_02114 | Hypothetical protein | 1.68 | 46.34 |
| PADG_02336 | Hypothetical protein | 10.07 | 11.27 |
| PADG_02343 | Hypothetical protein | 3.09 | 121.43 |
| PADG_02658 | Hypothetical protein | 7.78 | 42.62 |
| PADG_02678 | Hypothetical protein | 2.50 | 27.16 |
| PADG_02948 | Hypothetical protein | 2.51 | 31.04 |
| PADG_02967 | Hypothetical protein | 1.61 | 324.58 |
| PADG_03210 | Hypothetical protein | 1.75 | 36.54 |
| PADG_03410 | Hypothetical protein | 11.70 | 6.83 |
| PADG_03429 | Hypothetical protein | 2.05 | 22.15 |
| PADG_03570 | Hypothetical protein | 1.86 | 26.11 |
| PADG_03745 | Hypothetical protein | 2.13 | 5.13 |
| PADG_03785 | Hypothetical protein | 11.66 | 48.20 |
| PADG_03835 | Hypothetical protein | 2.78 | 15.35 |
| PADG_03886 | Hypothetical protein | 3.78 | 105.12 |
| PADG_04002 | Hypothetical protein | 2.60 | 33.65 |
| PADG_04243 | Hypothetical protein | 1.55 | 24.34 |
| PADG_04343 | Hypothetical protein | 1.76 | 22.53 |
| PADG_04389 | Hypothetical protein | 2.13 | 16.92 |
| PADG_04430 | Hypothetical protein | 3.08 | 22.69 |
| PADG_04457 | Hypothetical protein | 3.08 | 29.71 |
| PADG_04907 | Hypothetical protein | 3.07 | 62.07 |
| PADG_05152 | Hypothetical protein | 1.60 | 17.34 |
| PADG_05157 | Hypothetical protein | 6.80 | 25.47 |
| PADG_05445 | Hypothetical protein | 4.94 | 5.68 |
| PADG_05600 | Hypothetical protein | 77.87 | 17.39 |
| PADG_05703 | Hypothetical protein | 1.79 | 38.74 |
| PADG_06037 | Hypothetical protein | 1.56 | 16.30 |
| PADG_06080 | Hypothetical protein | 3.66 | 48.50 |
| PADG_06136 | Hypothetical protein | 1.54 | 7.14 |
| PADG_06179 | Hypothetical protein | 2.19 | 21.43 |
| PADG_06514 | Hypothetical protein | 1.61 | 12.22 |
| PADG_06893 | Hypothetical protein | 2.17 | 11.70 |
| PADG_07103 | Hypothetical protein | 1.69 | 16.18 |
| PADG_07355 | Hypothetical protein | 2.67 | 67.39 |
| PADG_07402 | Hypothetical protein | 6.20 | 21.50 |
| PADG_07670 | Hypothetical protein | 1.64 | 70.44 |
| PADG_08218 | Hypothetical protein | 2.72 | 21.19 |
| PADG_08724 | Hypothetical protein | 1.94 | 65.47 |
| PADG_11063 | Hypothetical protein | 1.81 | 5.88 |
| PADG_11100 | Hypothetical protein | 4.89 | 10.55 |
| PADG_11101 | Hypothetical protein | 3.57 | 69.11 |
| PADG_11247 | Hypothetical protein | 1.71 | 10.44 |
| PADG_11360 | Hypothetical protein | 4.95 | 12.00 |
| PADG_11437 | Hypothetical protein | 23.50 | 5.36 |
| PADG_11941 | Hypothetical protein | 80.02 | 6.19 |
| PADG_12124 | Hypothetical protein | 3.30 | 22.94 |
| PADG_12272 | Hypothetical protein | 2.17 | 12.38 |
| PADG_12309 | Hypothetical protein | 1.81 | 18.83 |
| PADG_12353 | Hypothetical protein | 36.57 | 17.78 |
| PADG_12502 | Hypothetical protein | 11.61 | 16.41 |
| PADG_06898 | Hypothetical protein | 1.75 | 63.05 |
| PADG_01935 | Hypothetical protein | 2.28 | 113.43 |
| PADG_00627 | Hypothetical protein | 9.28 | 55.50 |
| PADG_01632 | Hypothetical protein | 2.11 | 29.79 |
| PADG_02887 | Hypothetical protein | 1.99 | 90.86 |
| PADG_04612 | Hypothetical protein | 7.43 | 21.66 |
| PADG_08603 | Hypothetical protein | 3.52 | 10.80 |
| PADG_01626 | Hypothetical protein | 3.48 | 52.96 |
| PADG_07214 | Hypothetical protein | 1.63 | 37.18 |
| PADG_01630 | Hypothetical protein | 1.90 | 62.33 |
| PADG_04817 | Hypothetical protein | 2.02 | 37.78 |
| PADG_01047 | Hypothetical protein | 4.23 | 43.43 |

^a^ Identification of differentially regulated proteins from *Paracoccidioides* genome database (http://www.broadinstitute.org/annotation/genome/paracoccidioides_brasiliensis/MultiHome.html) using the ProteinLynx Global Server vs. 2.4 (PLGS) (Waters Corporation, Manchester, UK).

^b^ Proteins annotation from *Paracoccidioides* genome database or by homology from NCBI database (<http://www.ncbi.nlm.nih.gov/>).

^c^ Acetate/Glucose means: The level of expression in yeast cells derived from cultured in sodium acetate divided by the level in the control yeast cells cultured in glucose.

^d^ Biological process of differentially expressed proteins from MIPS (http://mips.helmholtz-muenchen.de/funcatDB/) and Uniprot databases (http://www.uniprot.org/).
