# Supplementary material for: Differential Metabolism of a Two-Carbon Substrate by Members of the Paracoccidioides Genus
Source: Front Microbiol. 2017 Nov 27;8:2308. doi: 10.3389/fmicb.2017.02308 (PMC5711815; doi:10.3389/fmicb.2017.02308)
Supplement: Supplementary file 8 [file Table8.DOCX]

**Supplemental Table 8: Proteins down-regulated in** *Paracoccidioides brasiliensis* **isolate EPM83 after growth for 48 hours in sodium acetate as carbon source.**

| **Accession number^a^** | **Protein Description^b^** | **Acetate/Glucose Ratio^c^** | **Score** |
| --- | --- | --- | --- |
| **Functional categories^d^** | |  |  |
| **1- METABOLISM** | | | |
| **Amino acid metabolism** | | | |
| PADG_03984 | Glucosamine-fructose-6-phosphateaminotransferase | 0.11 | 374.05 |
| PADG_05888 | Aspartokinase | 0.57 | 65.69 |
| PADG_06955 | Tryptophan synthase | 0.33 | 90.45 |
| PADG_02914 | Aminomethyltransferase | 0.54 | 348.25 |
| PADG_08648 | D-3-phosphoglycerate dehydrogenase | 0.47 | 37.89 |
| PADG_01621 | Aspartate aminotransferase | 0.38 | 273.91 |
| PADG_07010 | Urease accessory protein ureG | 0.59 | 93.75 |
| PADG_01536 | Glutamine synthetase | 0.46 | 55.97 |
| PADG_07146 | Pyrroline-5-carboxylate reductase | 0.19 | 16.90 |
| PADG_04689 | Acetylglutamate kinase | 0.60 | 205.14 |
| PADG_00888 | Argininosuccinate synthase | 0.55 | 297.70 |
| PADG_01404 | Aspartate aminotransferase | 0.52 | 253.15 |
| PADG_04356 | Cystathionine gamma-synthase | 0.62 | 74.93 |
| PADG_03500 | Glutamate-cysteine ligase | 0.52 | 34.07 |
| PADG_01615 | Homocitrate synthase | 0.32 | 243.77 |
| PADG_04487 | Chorismate synthase | 0.57 | 23.59 |
| PADG_00386 | Phospho-2-dehydro-3-deoxyheptonate aldolase | 0.60 | 95.20 |
| PADG_00215 | Aromatic-L-amino-acid decarboxylase | 0.52 | 83.41 |
| PADG_02263 | 5-proFAR isomerase | 0.51 | 22.11 |
| PADG_08304 | Acetolactate synthase small subunit | 0.46 | 123.00 |
| PADG_06671 | 3-isopropylmalate dehydrogenase A | 0.51 | 93.20 |
|  |  |  |  |
| **Nucleotide/nucleoside/nucleobase metabolism** | | | |
| PADG_04293 | Adenine phosphoribosyltransferase | 0.38 | 116.43 |
| PADG_06112 | Phosphoribosylaminoimidazole-succinocarboxamidesynthase | 0.47 | 26.67 |
| PADG_06585 | GMP synthase | 0.57 | 159.49 |
| PADG_00331 | Uricase | 0.58 | 119.92 |
| PADG_08530 | Thymidylate synthase | 0.54 | 53.15 |
| PADG_06897 | mRNA turnover protein | 0.32 | 6.51 |
|  |  |  |  |
| **C-compound and carbohydrate metabolism** | | | |
| PADG_01697 | Carbonic anhydrase | 0.61 | 10.44 |
| PADG_01745 | Mannose-1-phosphate guanyltransferase | 0.62 | 155.31 |
| PADG_03943 | Phosphomannomutase | 0.48 | 189.31 |
| PADG_08474 | Mannose-1-phosphate guanyltransferase | 0.47 | 174.14 |
| PADG_04374 | UTP-glucose-1-phosphate uridylyltransferase | 0.19 | 237.33 |
| PADG_08100 | NADP:D-xylose dehydrogenase | 0.44 | 5.38 |
| PADG_04900 | Alpha,alpha-trehalose-phosphate synthase | 0.62 | 64.63 |
| PADG_12009 | Trehalose 6-phosphate synthase/phosphatase | 0.55 | 5.74 |
|  |  |  |  |
| **Lipid, fatty acid and isoprenoid metabolism** | | | |
| PADG_02751 | Acetyl-CoA acetyltransferase | 0.49 | 250.18 |
| PADG_00254 | Fatty acid synthase subunit alpha reductase | 0.55 | 713.59 |
| PADG_00255 | Fatty acid synthase subunit beta dehydratase | 0.59 | 846.61 |
| PADG_00434 | Long-chain-fatty-acid-CoA ligase | 0.59 | 38.09 |
| PADG_02789 | 3-ketoacyl-CoA thiolase | 0.42 | 63.41 |
| PADG_03194 | 3-ketoacyl-CoA thiolase B | 0.60 | 184.05 |
| PADG_05130 | Long-chain specific acyl-CoA dehydrogenase | 0.62 | 85.67 |
| PADG_05431 | Phosphomevalonate kinase | 0.40 | 39.02 |
|  |  |  |  |
| **Metabolism of vitamins, cofactors, and prosthetic groups** | | | |
| PADG_02384 | Delta-aminolevulinic acid dehydratase | 0.33 | 16.71 |
| PADG_05357 | Thiamine-phosphate pyrophosphorylase | 0.49 | 28.11 |
| PADG_07528 | Methylenetetrahydrofolate reductase | 0.38 | 165.22 |
| PADG_00464 | Biotin-protein ligase | 0.54 | 133.36 |
|  |  |  |  |
| **Secondary metabolism** | | | |
| PADG_00262 | Inositol monophosphatase | 0.51 | 66.08 |
|  |  |  |  |
| **2- ENERGY** | | | |
| **Glycolysis and gluconeogenesis** | | | |
| PADG_03813 | Hexokinase | 0.64 | 195.24 |
| PADG_07202 | Fructose-2,6-bisphosphatase | 0.29 | 25.21 |
|  |  |  |  |
| **Tricarboxylic-acid pathway** | | | |
| PADG_07213 | Pyruvate dehydrogenase protein X component | 0.40 | 343.13 |
| PADG_04827 | Acyl-coenzyme A synthetase O-MACS | 0.62 | 17.07 |
| PADG_04993 | ATP-citrate synthase subunit 1 | 0.54 | 395.54 |
|  |  |  |  |
| **Electron transport and membrane-associated energy conservation** | | | |
| PADG_02912 | Vacuolar ATP synthase subunit C 1 | 0.64 | 146.07 |
| PADG_03175 | Vacuolar ATP synthase subunit F | 0.63 | 82.02 |
| PADG_04596 | Vacuolar ATP synthase 16 kDa proteolipid subunit | 0.51 | 6.33 |
| PADG_08391 | Plasma membrane ATPase | 0.24 | 52.75 |
| PADG_12152 | F-type H+-transporting ATPase subunit g | 0.25 | 46.39 |
|  |  |  |  |
| **3- CELL CYCLE AND DNA PROCESSING** | | | |
| PADG_08684 | WD repeat protein Cac2 | 0.40 | 61.63 |
| PADG_02683 | UV excision repair protein RAD23 | 0.64 | 60.74 |
| PADG_01646 | AAA family ATPase Pontin | 0.64 | 65.77 |
| PADG_04047 | Serine/threonine-protein phosphatase PP1 | 0.63 | 6.63 |
| PADG_01405 | Protein phosphatase PP2A regulatory subunit A | 0.30 | 85.86 |
| PADG_06697 | Poly(A) polymerase pla1 | 0.31 | 17.81 |
| PADG_03531 | Elongation factor 1-alpha | 0.38 | 108.28 |
| PADG_02359 | Cell division control protein | 0.40 | 62.16 |
| PADG_02900 | Tubulin beta chain | 0.35 | 96.42 |
| PADG_00128 | Tubulin alpha chain | 0.21 | 191.55 |
| PADG_12076 | Actin beta/gamma 1 | 0.66 | 133.20 |
| PADG_12077 | Actin beta/gamma 1 | 0.43 | 230.47 |
| PADG_07477 | Ankyrin repeat protein | 0.06 | 5.83 |
| PADG_02069 | C-type cyclin | 0.28 | 4.81 |
| PADG_02763 | Cyclin-dependent kinases regulatory subunit | 0.55 | 35.60 |
| PADG_03637 | Cell division control protein | 0.31 | 34.92 |
| PADG_06182 | Transcriptional repressor TUP1 | 0.56 | 218.31 |
| PADG_11857 | Cell cycle arrest protein BUB3 | 0.49 | 107.61 |
| PADG_05885 | Cell cycle control protein cwf14 | 0.60 | 28.49 |
| PADG_00932 | Eukaryotic translation initiation factor 3 | 0.57 | 88.07 |
| PADG_05893 | Nucleosome assembly protein | 0.39 | 202.55 |
|  |  |  |  |
| **4- TRANSCRIPTION** | | | |
| PADG_07962 | MADS box transcription factor Mcm1 | 0.25 | 28.80 |
| PADG_04307 | mRNA binding post-transcriptional regulator (Csx1) | 0.49 | 133.52 |
| PADG_05545 | Pre-mRNA-splicing factor ATP-dependent RNA helicase | 0.41 | 105.77 |
| PADG_01783 | Splicing factor 3a subunit 2 | 0.46 | 72.45 |
| PADG_08423 | Transcriptional regulator | 0.36 | 84.89 |
| PADG_03074 | Transcription elongation factor spt5 | 0.56 | 64.74 |
| PADG_00568 | General negative regulator of transcription subunit 1 | 0.54 | 111.85 |
| PADG_05975 | Exosome complex exonuclease RRP4 | 0.03 | 5.20 |
| PADG_00718 | Histone chaperone asf1 | 0.58 | 19.36 |
| PADG_01455 | KH domain RNA-binding protein | 0.59 | 119.48 |
|  |  |  |  |
| **5- PROTEIN SYNTHESIS** | | | |
| PADG_02445 | 40S ribosomal protein S15 | 0.63 | 76.46 |
| PADG_03315 | 40S ribosomal protein S4 | 0.63 | 224.26 |
| PADG_04493 | SUMO-conjugating enzyme ubc9 | 0.45 | 29.86 |
| PADG_00080 | Translation initiation factor eIF3 | 0.45 | 158.08 |
| PADG_04672 | ATP-dependent RNA helicase SUB2 | 0.21 | 179.09 |
| PADG_07073 | Nonsense-mediated mRNA decay protein | 0.29 | 71.83 |
| PADG_00342 | Eukaryotic translation initiation factor 3 | 0.33 | 74.44 |
| PADG_00626 | Eukaryotic translation initiation factor 3 subunit E | 0.18 | 141.47 |
| PADG_08033 | Eukaryotic translation initiation factor 3 subunit B | 0.52 | 135.30 |
| PADG_08592 | Eukaryotic translation initiation factor 4E-1 | 0.15 | 56.33 |
| PADG_07285 | RNA binding domain-containing protein | 0.53 | 103.27 |
| PADG_11711 | ATP-dependent RNA helicase eIF4A | 0.25 | 188.21 |
| PADG_00002 | Alanyl-tRNA synthetase | 0.47 | 446.82 |
| PADG_04962 | Aspartyl-tRNA synthetase | 0.65 | 108.86 |
| PADG_04116 | Methionyl-tRNA synthetase | 0.39 | 32.26 |
|  |  |  |  |
| **6- PROTEIN FATE** | | | |
| PADG_00050 | T-complex protein 1 subunit alpha | 0.52 | 215.94 |
| PADG_00928 | T-complex protein 1 subunit gamma | 0.22 | 223.15 |
| PADG_03441 | T-complex protein 1 subunit alpha | 0.63 | 313.99 |
| PADG_05108 | Prefoldin beta subunit | 0.42 | 58.76 |
| PADG_05124 | Prefoldin subunit 3 | 0.44 | 11.47 |
| PADG_04034 | Mitochondrial protein import protein MAS5 | 0.31 | 166.98 |
| PADG_04048 | Small COPII coat GTPase sar1 | 0.47 | 61.99 |
| PADG_08048 | T-complex protein 1 subunit beta | 0.52 | 352.83 |
| PADG_01114 | Importin subunit alpha-1a | 0.22 | 52.42 |
| PADG_02637 | Ubiquitin-conjugating enzyme | 0.65 | 121.06 |
| PADG_06439 | NEDD8-conjugating enzyme Ubc12 | 0.66 | 39.57 |
| PADG_07925 | Ubiquitin-conjugating enzyme | 0.43 | 33.75 |
| PADG_03793 | Oligosaccharyltransferase alpha subunit ostA | 0.53 | 32.06 |
| PADG_05837 | Glycoprotein FP21 | 0.65 | 121.93 |
| PADG_02625 | 4-coumarate-CoA ligase | 0.49 | 271.98 |
| PADG_01021 | Actin monomer binding protein | 0.54 | 68.19 |
| PADG_05099 | Calcium/calmodulin-dependent protein kinase type I | 0.17 | 11.53 |
| PADG_06655 | Coatomer subunit delta | 0.60 | 71.28 |
| PADG_00300 | 26S protease regulatory subunit 8 | 0.33 | 138.38 |
| PADG_02636 | 26S protease regulatory subunit 4 | 0.59 | 53.96 |
| PADG_06851 | 26S proteasome non-ATPase regulatory subunit 11 | 0.60 | 65.33 |
| PADG_08095 | 26S proteasome regulatory subunit rpn-8 | 0.51 | 95.00 |
| PADG_00051 | 26S protease regulatory subunit 8 | 0.41 | 87.43 |
| PADG_00599 | 26S protease regulatory subunit 6A | 0.29 | 96.20 |
| PADG_11701 | 26S protease subunit RPT4 [ | 0.51 | 25.28 |
| PADG_05428 | Proteasome maturation factor | 0.60 | 52.17 |
| PADG_06051 | 26S proteasome regulatory subunit rpn5 | 0.57 | 74.27 |
|  |  |  |  |
| **7- PROTEIN WITH BINDING FUNCTION OR COFACTOR REQUIREMENT** | | | |
| PADG_00011 | Actin binding protein | 0.47 | 85.48 |
| PADG_03098 | DUF858 domain-containing protein | 0.44 | 38.14 |
| PADG_07714 | NTF2 and RRM domain-containing protein | 0.48 | 50.15 |
| PADG_02924 | G2/M phase checkpoint control protein Sum2 | 0.42 | 171.06 |
| PADG_06294 | Hsp70 nucleotide exchange factor fes1 | 0.36 | 7.56 |
| PADG_02207 | GTP-binding protein | 0.49 | 47.49 |
|  |  |  |  |
| **8- CELLULAR TRANSPORT, TRANSPORT FACILITIES AND TRANSPORT ROUTES** | | | |
| PADG_08263 | Outer mitochondrial membrane protein porin 1 | 0.60 | 96.07 |
| PADG_08725 | Translin-associated protein X | 0.50 | 26.51 |
| PADG_00282 | GTP-binding protein SAS1 | 0.41 | 70.36 |
| PADG_02203 | Membrane biogenesis protein Yop1 | 0.39 | 12.67 |
| PADG_02686 | Reduced viability upon starvation protein | 0.66 | 37.98 |
| PADG_04100 | Clathrin heavy chain 1 | 0.65 | 372.85 |
| PADG_03551 | F-actin-capping protein subunit alpha | 0.30 | 79.37 |
| PADG_07756 | F-actin-capping protein subunit beta | 0.49 | 47.59 |
| PADG_04965 | Coatomer subunit gamma-1 | 0.57 | 143.97 |
| PADG_07014 | Vesicular-fusion protein sec17 | 0.45 | 107.29 |
| PADG_05839 | Transport protein particle subunit bet5 | 0.34 | 21.89 |
|  |  |  |  |
| **9- CELLULAR COMMUNICATION/SIGNAL TRANSDUCTION MECHANISM** | | | |
| PADG_01787 | 1-phosphatidylinositol phosphodiesterase | 0.48 | 46.91 |
| PADG_02300 | Protein phosphatase 2C | 0.59 | 112.83 |
| PADG_08337 | GTP-binding protein rho1 | 0.56 | 160.54 |
| PADG_02153 | Mitogen-activated protein kinase HOG1 | 0.59 | 38.32 |
| PADG_06243 | cAMP-independent regulatory protein pac2 | 0.42 | 17.03 |
|  |  |  |  |
| **10- CELL RESCUE, DEFENSE AND VIRULENCE** | | | |
| **Stress response** | | | |
| PADG_03180 | DNA damage-inducible protein | 0.59 | 93.63 |
| PADG_03095 | Mitochondrial peroxiredoxin PRX1 | 0.22 | 198.88 |
| PADG_02761 | Heat shock protein SSB1 | 0.64 | 332.21 |
| PADG_00778 | Hsp70 | 0.55 | 240.21 |
| PADG_07715 | Heat shock protein | 0.19 | 881.46 |
|  |  |  |  |
| **Detoxification** | | | |
| PADG_00529 | Glutaredoxin | 0.39 | 45.72 |
| PADG_02846 | Glutaredoxin domain-containing protein | 0.37 | 47.47 |
| PADG_01551 | Thioredoxin reductase | 0.58 | 84.67 |
| PADG_03161 | Thioredoxin | 0.55 | 41.19 |
|  |  |  |  |
| **11-BIOGENESIS OF CELLULAR COMPONENTS** | | | |
| **Cell wall** | | | |
| PADG_05937 | chitin synthase activator | 0.33 | 39.53 |
|  |  |  |  |
| **12- MISCELLANEOUS** | | | |
| PADG_02587 | COP9 signalosome complex subunit 4 | 0.54 | 60.09 |
| PADG_03343 | developmental protein fluG | 0.43 | 42.01 |
| PADG_00922 | bud site selection protein | 0.48 | 142.57 |
| PADG_05341 | fimbrin | 0.32 | 237.90 |
| PADG_00219 | actin-like protein arp9 | 0.64 | 56.54 |
| PADG_00260 | DNA-binding protein HGH1 | 0.49 | 47.76 |
| PADG_01052 | 3-demethylubiquinone-9 3-methyltransferase | 0.43 | 33.32 |
| PADG_01160 | PCI domain-containing protein | 0.30 | 88.65 |
| PADG_01487 | OTU domain-containing protein 6B | 0.45 | 26.71 |
| PADG_01688 | DlpA domain-containing protein | 0.51 | 59.74 |
| PADG_03012 | FluG domain-containing protein | 0.59 | 41.19 |
| PADG_04223 | 2-dehydropantoate 2-reductase | 0.47 | 35.81 |
| PADG_05580 | PP-loop family protein | 0.59 | 28.42 |
| PADG_11395 | K(+)/H(+) antiporter 1 | 0.43 | 78.21 |
| PADG_11982 | vacuolar protein sorting-associated protein | 0.31 | 101.46 |
| PADG_12125 | phytanoyl-CoA dioxygenase family protein | 0.56 | 58.50 |
| PADG_12381 | rab geranylgeranyl transferase escort protein | 0.42 | 16.77 |
| PADG_01032 | DNA-binding protein, 42 kDa | 0.66 | 173.77 |
| PADG_04030 | 60S acidic ribosomal protein P0 | 0.67 | 159.12 |
| PADG_04420 | Peptide methionine sulfoxide reductase msrA | 0.56 | 91.23 |
| PADG_05308 | Type 2A phosphatase activator tip41 | 0.65 | 64.33 |
|  |  |  |  |
| **13-- UNCLASSIFIED** | | | |
| PADG_04012 | Hypothetical protein | 0.63 | 22.15 |
| PADG_03005 | Hypothetical protein | 0.32 | 39.52 |
| PADG_11649 | Hypothetical protein | 0.66 | 17.20 |
| PADG_03698 | Hypothetical protein | 0.66 | 26.03 |
| PADG_00602 | Hypothetical protein | 0.66 | 32.42 |
| PADG_02526 | Hypothetical protein | 0.49 | 11.04 |
| PADG_02349 | Hypothetical protein | 0.26 | 11.64 |
| PADG_02919 | Hypothetical protein | 0.64 | 39.48 |
| PADG_03431 | Hypothetical protein | 0.57 | 181.38 |
| PADG_05627 | Hypothetical protein | 0.58 | 61.62 |
| PADG_00206 | Hypothetical protein | 0.63 | 36.67 |
| PADG_00211 | Hypothetical protein | 0.64 | 75.91 |
| PADG_00316 | Hypothetical protein | 0.24 | 17.13 |
| PADG_00421 | Hypothetical protein | 0.50 | 26.80 |
| PADG_02887 | Hypothetical protein | 0.21 | 80.60 |
| PADG_00541 | Hypothetical protein | 0.27 | 12.51 |
| PADG_00939 | Hypothetical protein | 0.52 | 10.45 |
| PADG_01287 | Hypothetical protein | 0.52 | 19.35 |
| PADG_01516 | Hypothetical protein | 0.56 | 27.11 |
| PADG_01857 | Hypothetical protein | 0.45 | 35.81 |
| PADG_02044 | Hypothetical protein | 0.19 | 38.01 |
| PADG_02086 | Hypothetical protein | 0.39 | 66.57 |
| PADG_02439 | Hypothetical protein | 0.41 | 12.34 |
| PADG_02478 | Hypothetical protein | 0.30 | 14.23 |
| PADG_04924 | Hypothetical protein | 0.50 | 80.63 |
| PADG_02666 | Hypothetical protein | 0.23 | 27.74 |
| PADG_03185 | Hypothetical protein | 0.64 | 31.76 |
| PADG_04430 | Hypothetical protein | 0.39 | 37.36 |
| PADG_04438 | Hypothetical protein | 0.07 | 5.78 |
| PADG_04457 | Hypothetical protein | 0.44 | 12.33 |
| PADG_04509 | Hypothetical protein | 0.35 | 10.73 |
| PADG_05294 | Hypothetical protein | 0.60 | 10.47 |
| PADG_05408 | Hypothetical protein | 0.61 | 17.33 |
| PADG_06620 | Hypothetical protein | 0.07 | 5.36 |
| PADG_07103 | Hypothetical protein | 0.57 | 40.93 |
| PADG_07495 | Hypothetical protein | 0.28 | 29.75 |
| PADG_07520 | Hypothetical protein | 0.54 | 70.84 |
| PADG_07633 | Hypothetical protein | 0.43 | 27.89 |
| PADG_08037 | Hypothetical protein | 0.64 | 34.90 |
| PADG_08065 | Hypothetical protein | 0.52 | 22.79 |
| PADG_04586 | Hypothetical protein | 0.33 | 17.06 |
| PADG_04311 | Hypothetical protein | 0.64 | 66.10 |
| PADG_08345 | Hypothetical protein | 0.17 | 16.41 |
| PADG_08715 | Hypothetical protein | 0.65 | 155.19 |
| PADG_01583 | Hypothetical protein | 0.39 | 16.15 |
| PADG_08368 | Hypothetical protein | 0.61 | 196.20 |
| PADG_11090 | Hypothetical protein | 0.53 | 23.18 |
| PADG_11191 | Hypothetical protein | 0.57 | 20.26 |
| PADG_00187 | Hypothetical protein | 0.62 | 75.89 |
| PADG_11593 | Hypothetical protein | 0.00 | 10.93 |
| PADG_11719 | Hypothetical protein | 0.02 | 5.47 |
| PADG_11913 | Hypothetical protein | 0.49 | 22.81 |
| PADG_11921 | Hypothetical protein | 0.55 | 9.39 |
| PADG_12050 | Hypothetical protein | 0.57 | 23.38 |
| PADG_12244 | Hypothetical protein | 0.39 | 13.13 |

^a^ Identification of differentially regulated proteins from *Paracoccidioides* genome database (http://www.broadinstitute.org/annotation/genome/paracoccidioides_brasiliensis/MultiHome.html) using the ProteinLynx Global Server vs. 2.4 (PLGS) (Waters Corporation, Manchester, UK).

^b^ Proteins annotation from *Paracoccidioides* genome database or by homology from NCBI database (<http://www.ncbi.nlm.nih.gov/>).

^c^ Acetate/Glucose means: The level of expression in yeast cells derived from cultured in sodium acetate divided by the level in the control yeast cells cultured in glucose.

^d^ Biological process of differentially expressed proteins from MIPS (http://mips.helmholtz-muenchen.de/funcatDB/) and Uniprot databases (http://www.uniprot.org/).
