# Supplementary material for: Differential Metabolism of a Two-Carbon Substrate by Members of the Paracoccidioides Genus
Source: Front Microbiol. 2017 Nov 27;8:2308. doi: 10.3389/fmicb.2017.02308 (PMC5711815; doi:10.3389/fmicb.2017.02308)
Supplement: Supplementary file 9 [file Table9.DOCX]

**Supplemental Table 9: Proteins up-regulated in** *Paracoccidioides brasiliensis* **isolate EPM83 after growth for 48 hours in sodium acetate as carbon source.**

| **Accession number^a^** | **Protein Description^b^** | **Acetate/Glucose Ratio^c^** | **Score** |
| --- | --- | --- | --- |
| **Functional categories^d^** | |  |  |
| **1- METABOLISM** | | | |
| **Amino acid metabolism** | | | |
| PADG_00210 | Glycine dehydrogenase | 2.70 | 371.67 |
| PADG_05085 | Delta-1-pyrroline-5-carboxylate dehydrogenase | 3.20 | 366.33 |
| PADG_04062 | Peptidase family protein | 2.05 | 117.81 |
| PADG_08662 | Cystathionine beta-lyase | 2.35 | 61.59 |
| PADG_02726 | Cysteine synthase | 2.59 | 219.17 |
| PADG_01886 | Adenosylhomocysteinase | 1.98 | 341.59 |
| PADG_04522 | Homoserine kinase | 2.01 | 60.82 |
| PADG_08376 | Aspartate-semialdehyde dehydrogenase | 1.94 | 178.88 |
| PADG_00405 | Choline dehydrogenase | 1.78 | 133.41 |
| PADG_00402 | 5-carboxymethyl-2-hydroxymuconate semialdehyde dehydrogenase | 1.55 | 124.04 |
| PADG_01228 | 3-hydroxybutyryl-CoA dehydrogenase | 1.76 | 173.26 |
| PADG_00336 | Prephenate dehydrogenase | 2.02 | 86.09 |
| PADG_07214 | Precorrin-2 dehydrogenase | 5.61 | 63.03 |
| PADG_02728 | Sulfite oxidase | 1.58 | 174.66 |
| PADG_08465 | Fumarylacetoacetase | 1.61 | 284.68 |
| PADG_07609 | Dihydroxy-acid dehydratase | 1.82 | 169.14 |
| PADG_04570 | Branched-chain-amino-acid aminotransferase | 1.86 | 192.92 |
| PADG_07370 | Methylcrotonoyl-CoA carboxylase beta chain | 1.70 | 192.95 |
| PADG_00643 | Mitochondrial methylglutaconyl-CoA hydratase | 2.31 | 137.03 |
| PADG_01328 | Ornithine aminotransferase | 1.64 | 218.66 |
| PADG_00637 | Arginase | 1.57 | 83.84 |
| PADG_00663 | Homoserine dehydrogenase | 1.85 | 165.59 |
| PADG_05277 | Serine hydroxymethyltransferase | 1.71 | 250.23 |
| PADG_01963 | Glycine cleavage system H protein | 1.52 | 117.87 |
| PADG_08604 | ATP phosphoribosyltransferase | 1.75 | 36.85 |
| PADG_00349 | Kynureninase | 2.15 | 34.44 |
| PADG_08466 | Homogentisate 1,2-dioxygenase | 2.93 | 196.05 |
| PADG_00875 | Pentafunctional AROM polypeptide | 1.75 | 475.88 |
| PADG_03466 | 3-hydroxyisobutyrate dehydrogenase | 1.52 | 250.46 |
|  |  |  |  |
| **Nitrogen, sulfur and selenium metabolism** | | | |
| PADG_01150 | 2-nitropropane dioxygenase | 1.64 | 121.25 |
| PADG_01061 | Cyanate hydratase | 3.94 | 68.89 |
| PADG_03852 | HIRA interacting protein | 1.76 | 29.45 |
| PADG_00446 | Oxidoreductase 2-nitropropane dioxygenase family | 2.47 | 266.23 |
|  |  |  |  |
| **Nucleotide/nucleoside/nucleobase metabolism** | | | |
| PADG_02246 | Adenosine kinase | 1.58 | 109.66 |
| PADG_04340 | DNA-directed RNA polymerase II subunit RPB2 | 2.06 | 193.11 |
| PADG_06424 | DNA-directed RNA polymerase III subunit RPC1 | 1.57 | 315.75 |
| PADG_08066 | Purine nucleoside phosphorylase | 1.61 | 44.65 |
| PADG_07918 | Pyrimidine 5'-nucleotidase | 2.04 | 15.15 |
| PADG_02493 | Ureidoglycolate lyase | 6.09 | 17.60 |
|  |  |  |  |
| **C-compound and carbohydrate metabolism** | | | |
| PADG_02123 | Aflatoxin B1 aldehyde reductase member 2 | 2.23 | 144.99 |
| PADG_00401 | Glucosamine-6-phosphate isomerase | 1.53 | 21.02 |
| PADG_06765 | NADP-dependent leukotriene B4 12-hydroxydehydrogenase | 2.40 | 92.25 |
| PADG_03859 | NADP-dependent mannitol dehydrogenase | 1.81 | 171.99 |
| PADG_00604 | Phosphoacetylglucosamine mutase | 1.70 | 300.70 |
| PADG_03710 | Retrograde regulation protein | 1.82 | 168.78 |
| PADG_04312 | UDP-N-acetylglucosamine pyrophosphorylase | 1.52 | 253.34 |
|  |  |  |  |
| **Lipid, fatty acid and isoprenoid metabolism** | | | |
| PADG_01687 | 3-ketoacyl-CoA thiolase | 1.96 | 309.73 |
| PADG_06721 | 3-oxoacyl-[acyl-carrier-protein] reductase | 2.39 | 44.20 |
| PADG_06805 | Acyl-CoA dehydrogenase | 2.47 | 272.08 |
| PADG_07023 | Carnitine O-acetyltransferase | 2.15 | 254.86 |
| PADG_06425 | Delta(3,5)-Delta(2,4)-dienoyl-CoA isomerase | 1.86 | 131.58 |
| PADG_01209 | Enoyl-CoA hydratase | 2.08 | 267.66 |
| PADG_12025 | Glutaryl-CoA dehydrogenase | 1.55 | 224.08 |
| PADG_07031 | Hydroxymethylglutaryl-CoA lyase | 2.29 | 117.10 |
| PADG_07411 | NAD-dependent 15-hydroxyprostaglandin dehydrogenase | 2.21 | 47.96 |
| PADG_00601 | Peroxisomal dehydratase | 3.01 | 145.59 |
| PADG_05281 | Propionate-CoA ligase | 3.50 | 103.15 |
| PADG_07699 | S-formylglutathione hydrolase | 1.65 | 70.58 |
| PADG_02527 | Short chain dehydrogenase family protein | 2.46 | 25.33 |
| PADG_04939 | Succinyl-CoA:3-ketoacid-coenzyme A transferase subunit B | 2.23 | 227.51 |
| PADG_00244 | Trans-2-enoyl-CoA reductase | 2.39 | 228.05 |
|  |  |  |  |
| **Metabolism of vitamins, cofactors, and prosthetic groups** | | | |
| PADG_03598 | 3-methyl-2-oxobutanoatehydroxymethyltransferase | 1.65 | 56.16 |
| PADG_03213 | 5-formyltetrahydrofolate cyclo-ligase | 1.58 | 46.61 |
| PADG_03983 | 6,7-dimethyl-8-ribityllumazine synthase | 1.72 | 81.54 |
| PADG_04879 | Uroporphyrinogen-III synthase | 2.40 | 11.99 |
| PADG_00821 | Glutamyl-tRNA synthetase | 2.69 | 436.08 |
| PADG_05490 | Molybdopterin binding domain-containing protein | 1.68 | 105.44 |
| PADG_01126 | Molybdopterin synthase large subunit CnxH | 1.63 | 10.02 |
| PADG_03697 | Protoporphyrinogen oxidase | 1.54 | 65.69 |
| PADG_12043 | Cysteine desulfurase, mitochondrial | 2.06 | 286.83 |
|  |  |  |  |
| **Secondary metabolism** | | | |
| PADG_04899 | Metallo-beta-lactamase domain-containing protein | 2.48 | 48.87 |
| PADG_02981 | ThiJ/PfpI family protein | 1.99 | 176.19 |
|  |  |  |  |
| **2-ENERGY** | | | |
| **Glycolysis and gluconeogenesis** | | | |
| PADG_06187 | 6-phosphofructo-2-kinase | 1.88 | 118.86 |
| PADG_00852 | Aldolase | 4.07 | 27.60 |
| PADG_01706 | Fructose-1,6-bisphosphatase | 1.56 | 173.53 |
|  |  |  |  |
| **Ethanol production** | | | |
| PADG_04701 | Alcohol dehydrogenase | 4.64 | 212.97 |
| PADG_11405 | Alcohol dehydrogenase 1 | 3.52 | 195.36 |
| PADG_03099 | Aldehyde dehydrogenase | 1.50 | 145.31 |
| PADG_03403 | Aldehyde dehydrogenase A | 2.18 | 161.09 |
| PADG_00714 | Pyruvate decarboxylase | 1.52 | 326.01 |
|  |  |  |  |
| **Tricarboxylic-acid pathway** | | | |
| PADG_01762 | 2-oxoglutarate dehydrogenase E1 | 1.56 | 503.51 |
| PADG_04994 | ATP-citrate-lyase | 1.55 | 215.40 |
| PADG_08387 | Citrate synthase | 2.18 | 304.62 |
| PADG_06494 | Dihydrolipoyl dehydrogenase | 1.61 | 417.52 |
| PADG_08119 | Fumarate hydratase | 1.79 | 220.38 |
| PADG_02592 | Fumarate reductase | 1.74 | 388.88 |
| PADG_03977 | Isocitrate dehydrogenase subunit 1 | 1.65 | 231.54 |
| PADG_07210 | Malate dehydrogenase | 1.62 | 408.49 |
| PADG_03268 | NADPH-dependent D-xylose reductase | 1.52 | 43.52 |
| PADG_00052 | Succinate dehydrogenase flavoprotein subunit | 2.25 | 188.33 |
| PADG_08013 | Succinate dehydrogenase iron-sulfur subunit | 4.24 | 62.30 |
| PADG_02260 | Succinyl-CoA ligase subunit alpha | 1.55 | 212.60 |
|  |  |  |  |
| **Glyoxylate cycle** | | | |
| PADG_01483 | Isocitrate lyase | 1.99 | 295.45 |
| PADG_04702 | Malate synthase | 2.11 | 260.12 |
|  |  |  |  |
| **Methylcytrate cycle** | | | |
| PADG_04710 | 2-methylcitrate synthase | 2.62 | 439.39 |
| PADG_04718 | 2-methylcitrate dehydratase | 1.89 | 507.90 |
| PADG_04709 | Mitochondrial 2-methylisocitrate lyase | 1.67 | 272.44 |
|  |  |  |  |
| **Electron transport and membrane-associated energy conservation** | | | |
| PADG_02561 | ATPase alpha subunit | 3.46 | 551.56 |
| PADG_02745 | NADH-ubiquinone oxidoreductase | 2.67 | 22.20 |
| PADG_04397 | cytochrome c oxidase subunit 4, mitochondrial | 1.61 | 44.68 |
| PADG_04501 | ubiquinol-cytochrome c reductase subunit 7 | 2.47 | 18.67 |
| PADG_05343 | NADH-ubiquinone oxidoreductase 21.3 kDa subunit | 2.09 | 4.29 |
| PADG_06978 | Cytochrome c | 3.25 | 124.44 |
| PADG_06995 | Cytochrome c oxidase polypeptide VIb [Paracoccidioides lutzii Pb01] | 1.97 | 43.20 |
| PADG_07042 | ATP synthase subunit 5 | 1.55 | 139.19 |
| PADG_08373 | F-type H+-transporting ATPase subunit epsilon [Exophiala dermatitidis NIH/UT8656] | 1.93 | 21.50 |
| PADG_08292 | Cytochrome-c oxidase chain VIIc | 1.62 | 6.83 |
| PADG_02578 | ATP synthase subunit 4 | 1.96 | 127.09 |
| PADG_03747 | Alternative oxidase | 3.55 | 31.20 |
| PADG_04729 | ATP synthase D chain, mitochondrial | 2.28 | 60.01 |
| PADG_05750 | Cytochrome c oxidase subunit 5a | 2.34 | 120.55 |
| PADG_02468 | F-type H+-transporting ATP synthase subunit | 1.89 | 32.29 |
| PADG_07813 | ATP synthase gamma chain | 1.85 | 92.56 |
| PADG_08349 | ATP synthase subunit beta | 4.44 | 414.51 |
| PADG_07789 | ATP synthase delta chain | 4.96 | 84.73 |
| PADG_08394 | Cytochrome b-c1 complex subunit 2 | 3.77 | 224.93 |
| PADG_01841 | Cytochrome c oxidase assembly protein COX19 | 1.90 | 54.09 |
| PADG_00688 | F-type H+-transporting ATPase subunit h | 5.20 | 19.80 |
| PADG_11981 | V-type H+-transporting ATPase subunit A | 1.93 | 284.14 |
| PADG_05290 | Cytochrome b5 | 1.80 | 30.70 |
| PADG_12148 | NADH-ubiquinone oxidoreductase 78 kDa subunit | 2.63 | 11.93 |
|  |  |  |  |
| **3-CELL CYCLE AND DNA PROCESSING** | | | |
| PADG_08606 | DNA mismatch repair protein Msh3 | 2.30 | 44.93 |
| PADG_01391 | DNA repair and recombination protein RAD26 | 2.09 | 164.70 |
| PADG_00237 | Histone-lysine N-methyltransferase | 2.94 | 40.25 |
| PADG_04614 | Prohibitin-1 | 1.66 | 99.13 |
| PADG_07835 | Replication factor-A protein | 1.84 | 99.63 |
| PADG_05798 | Single-strand binding protein family | 1.54 | 155.48 |
| PADG_00759 | Prefoldin subunit 4 | 1.94 | 49.66 |
| PADG_01668 | Centrin-3 | 1.90 | 28.73 |
| PADG_05906 | Histone H2a | 2.49 | 69.68 |
| PADG_00422 | Actin cytoskeleton protein (VIP1) | 1.53 | 206.56 |
|  |  |  |  |
| **4- TRANSCRIPTION** | | | |
| PADG_02752 | 116 kDa U5 small nuclear ribonucleoprotein component | 1.87 | 242.69 |
| PADG_07629 | C2H2 finger domain-containing protein | 3.29 | 91.62 |
| PADG_00873 | Histone H3 | 1.87 | 86.33 |
| PADG_00872 | Histone H4 | 2.09 | 123.77 |
| PADG_07134 | Histone H4.2 | 2.06 | 153.08 |
| PADG_04521 | Mediator of RNA polymerase II transcription subunit 5 | 2.73 | 53.58 |
| PADG_02555 | RNA binding domain-containing protein | 1.92 | 289.49 |
| PADG_06666 | RNA splicing factor Pad-1 | 1.75 | 125.55 |
| PADG_05900 | Transcription elongation factor 1 | 1.70 | 16.43 |
| PADG_05924 | ATP-dependent RNA helicase DHH1 | 2.27 | 118.07 |
| PADG_05415 | ATP-dependent RNA helicase suv3 | 1.75 | 42.86 |
| PADG_02783 | RNA-binding La domain-containing protein | 1.57 | 131.64 |
| PADG_02825 | Splicing factor | 1.73 | 14.02 |
| PADG_05587 | U2 small nuclear ribonucleoprotein B | 2.56 | 81.31 |
| PADG_04301 | WD repeat-containing protein | 1.74 | 17.61 |
| PADG_06487 | D-tyrosyl-tRNA(Tyr) deacylase | 1.59 | 27.94 |
|  |  |  |  |
| **5- PROTEIN SYNTHESIS** | | | |
| PADG_05562 | Large subunit ribosomal protein L23 | 1.76 | 22.58 |
| PADG_00627 | Large subunit ribosomal protein L49 | 2.26 | 48.06 |
| PADG_08453 | Large subunit ribosomal protein L6 | 2.89 | 25.90 |
| PADG_11743 | Ribosomal RNA-processing protein 7 | 1.81 | 47.80 |
| PADG_01854 | Small subunit ribosomal protein S11 | 1.98 | 12.57 |
| PADG_04475 | Small subunit ribosomal protein YMR-31 | 2.07 | 48.83 |
| PADG_07519 | 50S ribosomal protein L1 | 2.42 | 4.69 |
| PADG_02056 | 50S ribosomal protein L12 | 2.20 | 127.81 |
| PADG_01647 | 50S ribosomal protein L24 | 1.61 | 25.15 |
| PADG_05177 | 54S ribosomal protein L9 | 1.66 | 10.54 |
| PADG_05847 | Brix domain-containing protein | 2.61 | 45.74 |
| PADG_05206 | Mitochondrial 54S ribosomal protein L38 | 1.55 | 17.56 |
| PADG_11083 | Ribosomal protein L13 | 1.94 | 24.15 |
| PADG_04449 | Ribosomal protein L23e | 1.80 | 91.17 |
| PADG_03829 | Ribosomal protein S18 | 1.62 | 54.89 |
| PADG_05118 | Translation initiation factor 3 subunit K | 1.74 | 46.73 |
| PADG_03046 | Elongation factor G 1 | 1.90 | 269.60 |
| PADG_04083 | Eukaryotic translation initiation factor 2 subunit gamma | 2.41 | 160.88 |
| PADG_05388 | Asparaginyl-tRNA synthetase | 1.80 | 147.91 |
| PADG_08472 | Lysyl-tRNA synthetase | 2.07 | 243.09 |
| PADG_05897 | Seryl-tRNA synthetase | 1.72 | 275.50 |
| PADG_00821 | Glutamyl-tRNA synthetase | 2.69 | 436.08 |
|  |  |  |  |
| **6-PROTEIN FATE** | | | |
| PADG_02895 | ATP-dependent Clp protease ATP-binding subunit ClpB | 1.50 | 191.00 |
| PADG_00501 | DnaJ domain-containing protein | 2.66 | 49.04 |
| PADG_08587 | FK506-binding protein | 1.79 | 39.06 |
| PADG_06992 | Mitochondrial co-chaperone GrpE | 1.93 | 212.32 |
| PADG_05129 | UDP-glucose:glycoprotein glucosyltransferase | 1.76 | 256.94 |
| PADG_02431 | Translocation protein SEC66 | 1.53 | 24.26 |
| PADG_04241 | Coatomer subunit alpha | 1.77 | 227.55 |
| PADG_00839 | Diphthine synthase | 1.61 | 23.41 |
| PADG_02197 | DNA damage tolerance protein rad31 | 1.51 | 4.87 |
| PADG_04063 | Histidine kinase M7 | 1.83 | 59.52 |
| PADG_07156 | Histone acetyltransferase type B catalytic subunit | 1.56 | 168.09 |
| PADG_06766 | Mitochondrial-processing peptidase subunit beta | 2.05 | 230.10 |
| PADG_05980 | Protein kinase byr1 | 1.53 | 33.13 |
| PADG_07834 | Ubiquitin carboxyl-terminal hydrolase | 2.22 | 163.85 |
| PADG_05335 | Iron sulfur cluster assembly protein | 1.50 | 74.19 |
| PADG_04167 | Aspartyl aminopeptidase | 1.73 | 270.11 |
| PADG_08109 | Gamma-aminobutyric acid receptor associated protein | 1.64 | 15.26 |
| PADG_04125 | Mitochondrial presequence protease | 2.10 | 384.78 |
| PADG_03982 | Proteasome component C1 | 1.50 | 132.00 |
| PADG_03965 | Proteasome component Pre4 | 1.65 | 114.48 |
| PADG_08442 | Proteasome component Y13 | 1.70 | 152.27 |
| PADG_07422 | Subtilase-type proteinase psp3 | 1.57 | 97.95 |
| PADG_05193 | Xaa-Pro aminopeptidase | 2.10 | 149.15 |
| PADG_12323 | Peptidyl-prolyl cis-trans isomerase | 1.69 | 172.73 |
|  |  |  |  |
| **7- PROTEIN WITH BINDING FUNCTION OR COFACTOR REQUIREMENT** | | | |
| PADG_00352 | SH3 domain-containing protein | 2.05 | 47.10 |
| PADG_03091 | WD repeat-containing protein | 12.84 | 16.91 |
| PADG_06997 | Nuclear cap-binding protein | 3.98 | 30.03 |
|  |  |  |  |
| **8- CELLULAR TRANSPORT, TRANSPORT FACILITIES AND TRANSPORT ROUTES** | | | |
| PADG_05084 | Ctr copper transporter family protein | 2.40 | 5.70 |
| PADG_07415 | Phosphatidylinositol transporter | 4.55 | 37.70 |
| PADG_01440 | ADP,ATP carrier protein | 1.65 | 136.43 |
| PADG_01847 | Stomatin family protein | 2.36 | 33.02 |
| PADG_02640 | ATP-binding cassette sub-family F member 2 | 1.70 | 137.14 |
| PADG_04883 | Transport protein SEC24 | 2.15 | 60.43 |
| PADG_07288 | Polyubiquitin binding protein | 1.72 | 27.32 |
| PADG_06998 | Transport protein SEC31 | 1.51 | 197.35 |
|  |  |  |  |
| **9- CELL RESCUE, DEFENSE AND VIRULENCE** | | | |
| **Stress response** | | | |
| PADG_03963 | 30 kDa heat shock protein | 2.37 | 226.88 |
| PADG_08369 | Heat shock protein | 2.23 | 833.68 |
| PADG_03562 | Hsp70-like protein | 1.54 | 536.57 |
| PADG_00430 | Heat shock protein SSC1 | 1.81 | 606.34 |
|  |  |  |  |
| **Detoxification** | | | |
| PADG_01954 | Superoxide dismutase Fe/Mn SOD5 | 2.28 | 90.74 |
| PADG_07418 | Superoxide dismutase Cu/Zn SOD1 | 2.49 | 76.85 |
| PADG_01755 | Superoxide dismutase Fe/Mn SOD2 | 3.13 | 141.53 |
| PADG_03423 | Glutathione S-transferase Gst3 | 2.74 | 143.10 |
| PADG_04587 | Peroxiredoxin HYR1 | 1.57 | 17.20 |
| PADG_00324 | Peroxisomal catalase | 2.00 | 318.42 |
| PADG_07815 | Disulfide-isomerase | 1.73 | 224.03 |
|  |  |  |  |
| **11-BIOGENESIS OF CELLULAR COMPONENTS** | | | |
| **Cell wall** | | | |
| PADG_02865 | Cell wall biogenesis protein phosphatase Ssd1 | 1.75 | 88.42 |
|  |  |  |  |
| **12- MISCELLANEOUS** | | | |
| PADG_12145 | Uracil-regulated protein 1 | 1.96 | 94.70 |
| PADG_11421 | Histone H2A.Z | 1.77 | 93.26 |
| PADG_07836 | Quinone oxidoreductase | 3.32 | 197.36 |
| PADG_08034 | Dienelactone hydrolase family protein | 1.62 | 94.41 |
| PADG_05010 | Arrestin domain-containing protein | 1.65 | 68.59 |
| PADG_03638 | Oxidoreductase | 2.11 | 17.77 |
| PADG_03436 | 3' exoribonuclease family protein | 2.63 | 30.70 |
| PADG_03526 | M protein repeat protein | 1.92 | 345.35 |
| PADG_03544 | Ser/Thr protein phosphatase family protein | 1.62 | 198.85 |
| PADG_00345 | HHE domain-containing protein | 1.55 | 21.23 |
| PADG_01862 | Stress responsive A/B barrel domain-containing protein | 3.18 | 16.31 |
| PADG_03031 | CobW domain-containing protein | 2.61 | 216.78 |
| PADG_03316 | MYB DNA-binding domain-containing protein | 2.14 | 37.99 |
| PADG_02596 | Transcriptional regulatory protein SIN3 | 1.72 | 79.43 |
| PADG_12204 | Hydroxyacylglutathione hydrolase | 1.54 | 39.73 |
|  |  |  |  |
| **13-UNCLASSIFIED** | | | |
| PADG_05474 | Hypothetical protein | 1.79 | 143.49 |
| PADG_11446 | Hypothetical protein | 1.64 | 38.40 |
| PADG_00046 | Hypothetical protein | 2.64 | 26.87 |
| PADG_00060 | Hypothetical protein | 1.55 | 59.84 |
| PADG_00388 | Hypothetical protein | 3.00 | 45.95 |
| PADG_00459 | Hypothetical protein | 2.89 | 44.14 |
| PADG_00465 | Hypothetical protein | 1.57 | 14.24 |
| PADG_00496 | Hypothetical protein | 3.66 | 63.52 |
| PADG_00646 | Hypothetical protein | 1.67 | 72.31 |
| PADG_00674 | Hypothetical protein | 1.55 | 41.06 |
| PADG_01488 | Hypothetical protein | 2.17 | 199.11 |
| PADG_01618 | Hypothetical protein | 3.02 | 16.62 |
| PADG_01727 | Hypothetical protein | 4.66 | 36.73 |
| PADG_01855 | Hypothetical protein | 8.31 | 62.66 |
| PADG_01867 | Hypothetical protein | 1.56 | 110.12 |
| PADG_01991 | Hypothetical protein | 2.19 | 5.66 |
| PADG_01885 | Hypothetical protein | 5.48 | 16.89 |
| PADG_01560 | Hypothetical protein | 2.63 | 11.15 |
| PADG_02272 | Hypothetical protein | 2.56 | 12.28 |
| PADG_02301 | Hypothetical protein | 5.83 | 5.69 |
| PADG_02307 | Hypothetical protein | 2.18 | 65.50 |
| PADG_02336 | Hypothetical protein | 2.85 | 6.14 |
| PADG_02557 | Hypothetical protein | 2.05 | 51.81 |
| PADG_02633 | Hypothetical protein | 1.79 | 22.17 |
| PADG_02658 | Hypothetical protein | 1.55 | 51.55 |
| PADG_02758 | Hypothetical protein | 1.67 | 47.43 |
| PADG_02759 | Hypothetical protein | 1.80 | 76.93 |
| PADG_02858 | Hypothetical protein | 1.75 | 45.83 |
| PADG_02878 | Hypothetical protein | 1.80 | 27.97 |
| PADG_03340 | Hypothetical protein | 2.52 | 57.47 |
| PADG_03570 | Hypothetical protein | 2.33 | 23.64 |
| PADG_07896 | Hypothetical protein | 3.09 | 33.95 |
| PADG_07064 | Hypothetical protein | 6.48 | 44.18 |
| PADG_03579 | Hypothetical protein | 2.14 | 20.54 |
| PADG_03612 | Hypothetical protein | 1.63 | 11.84 |
| PADG_03660 | Hypothetical protein | 1.57 | 134.38 |
| PADG_03761 | Hypothetical protein | 1.61 | 27.17 |
| PADG_03785 | Hypothetical protein | 1.67 | 50.65 |
| PADG_03995 | Hypothetical protein | 1.73 | 9.73 |
| PADG_04057 | Hypothetical protein | 2.06 | 135.28 |
| PADG_04229 | Hypothetical protein | 1.57 | 192.60 |
| PADG_04256 | Hypothetical protein | 1.67 | 15.97 |
| PADG_04423 | Hypothetical protein | 2.89 | 43.34 |
| PADG_04439 | Hypothetical protein | 1.82 | 53.57 |
| PADG_04494 | Hypothetical protein | 2.11 | 16.49 |
| PADG_04627 | Hypothetical protein | 4.86 | 11.06 |
| PADG_04685 | Hypothetical protein | 1.59 | 26.74 |
| PADG_04806 | Hypothetical protein | 2.25 | 22.68 |
| PADG_04907 | Hypothetical protein | 4.26 | 54.11 |
| PADG_04953 | Hypothetical protein | 1.92 | 32.62 |
| PADG_05104 | Hypothetical protein | 1.75 | 17.69 |
| PADG_05157 | Hypothetical protein | 6.53 | 30.67 |
| PADG_05701 | Hypothetical protein | 1.51 | 105.71 |
| PADG_05703 | Hypothetical protein | 1.68 | 58.47 |
| PADG_01796 | Hypothetical protein | 2.63 | 35.34 |
| PADG_06021 | Hypothetical protein | 1.98 | 17.33 |
| PADG_06136 | Hypothetical protein | 3.17 | 56.11 |
| PADG_06239 | Hypothetical protein | 2.69 | 11.40 |
| PADG_06846 | Hypothetical protein | 2.96 | 5.36 |
| PADG_06986 | Hypothetical protein | 1.51 | 10.53 |
| PADG_06974 | Hypothetical protein | 3.21 | 5.12 |
| PADG_07075 | Hypothetical protein | 1.66 | 23.18 |
| PADG_07298 | Hypothetical protein | 2.04 | 17.30 |
| PADG_07565 | Hypothetical protein | 5.36 | 39.97 |
| PADG_07696 | Hypothetical protein | 2.22 | 21.16 |
| PADG_07768 | Hypothetical protein | 2.63 | 50.70 |
| PADG_07793 | Hypothetical protein | 1.64 | 16.47 |
| PADG_08152 | Hypothetical protein | 1.69 | 75.18 |
| PADG_08214 | Hypothetical protein | 1.58 | 52.38 |
| PADG_08420 | Hypothetical protein | 1.67 | 32.45 |
| PADG_08478 | Hypothetical protein | 6.24 | 16.95 |
| PADG_08689 | Hypothetical protein | 1.50 | 17.72 |
| PADG_11044 | Hypothetical protein | 1.90 | 11.47 |
| PADG_11111 | Hypothetical protein | 1.74 | 34.44 |
| PADG_07689 | Hypothetical protein | 2.11 | 107.03 |
| PADG_11157 | Hypothetical protein | 1.62 | 65.96 |
| PADG_11170 | Hypothetical protein | 1.55 | 10.91 |
| PADG_11357 | Hypothetical protein | 4.71 | 9.10 |
| PADG_11487 | Hypothetical protein | 7.10 | 29.09 |
| PADG_11558 | Hypothetical protein | 1.52 | 12.09 |
| PADG_11833 | Hypothetical protein | 1.82 | 169.57 |
| PADG_11904 | Hypothetical protein | 3.51 | 729.03 |
| PADG_12072 | Hypothetical protein | 1.68 | 22.65 |
| PADG_12182 | Hypothetical protein | 6.69 | 38.51 |
| PADG_12186 | Hypothetical protein | 1.54 | 71.59 |
| PADG_12285 | Hypothetical protein | 1.87 | 6.72 |
| PADG_12292 | Hypothetical protein | 4.43 | 5.03 |
| PADG_12311 | Hypothetical protein | 1.73 | 11.36 |
| PADG_12392 | Hypothetical protein | 1.59 | 34.40 |
| PADG_00580 | Hypothetical protein | 1.55 | 47.40 |
| PADG_07449 | Hypothetical protein | 2.43 | 54.99 |
| PADG_03559 | Hypothetical protein | 5.98 | 11.45 |
| PADG_12408 | Hypothetical protein | 1.68 | 28.98 |
| PADG_12411 | Hypothetical protein | 1.63 | 5.73 |
| PADG_12419 | Hypothetical protein | 3.62 | 18.83 |
| PADG_12447 | Hypothetical protein | 2.01 | 56.85 |
| PADG_12503 | Hypothetical protein | 2.41 | 302.59 |

^a^ Identification of differentially regulated proteins from *Paracoccidioides* genome database (http://www.broadinstitute.org/annotation/genome/paracoccidioides_brasiliensis/MultiHome.html) using the ProteinLynx Global Server vs. 2.4 (PLGS) (Waters Corporation, Manchester, UK).

^b^ Proteins annotation from *Paracoccidioides* genome database or by homology from NCBI database (<http://www.ncbi.nlm.nih.gov/>).

^c^ Acetate/Glucose means: The level of expression in yeast cells derived from cultured in sodium acetate divided by the level in the control yeast cells cultured in glucose.

^d^ Biological process of differentially expressed proteins from MIPS (http://mips.helmholtz-muenchen.de/funcatDB/) and Uniprot databases (http://www.uniprot.org/).
