# Supplementary material for: Differential Metabolism of a Two-Carbon Substrate by Members of the Paracoccidioides Genus
Source: Front Microbiol. 2017 Nov 27;8:2308. doi: 10.3389/fmicb.2017.02308 (PMC5711815; doi:10.3389/fmicb.2017.02308)
Supplement: Supplementary file 10 [file Table10.DOCX]

**Supplemental Table 10: Synthesis of the proteins related to the main metabolic pathways in** *Paracoccidioides lutzii* **and** *P. brasiliensis* **isolates** **after growth for 48 hours in sodium acetate as carbon source.**

| **Functional categories^a^ / Protein description^b^** | **Acetate/Glucose Ratio^c^** | | | | **Acession number^d^** |
| --- | --- | --- | --- | --- | --- |
| **Isolates** | *Pb***01** | *Pb***03** | *Pb***339** | *Pb***EPM83** |  |
| **1. ENERGY** | | | | | |
| **Glycolysis / Gluconeogenesis** | | | | | |
| UTP-glucose-1-phosphate uridylyltransferase | 0.1060 | * | * | 0.1921 | PAAG_06817; PADG_04374 |
| Glycogen synthase | 2.3158 | * | 0.2000 | * | PAAG_07276; PADG_05778 |
| 1,4-alpha-glucan-branching enzyme | 1.9364 | * | * | * | PAAG_08038 |
| Glycogen phosphorylase | * | * | 0.3650 | * | PADG_02145 |
| Phosphoglucomutase | * | * | 1.8547 | * | PADG_11132 |
| Glucokinase | 2.1820 | 1.6758 | * | * | PAAG_06172; PABG_06480 |
| Hexokinase | 0.5411 | 1.7294 | * | 0.6444 | PAAG_01015; PABG_01237; PADG_03813 |
| Glucose-6-phosphate isomerase | * | 2.1592 | * | * | PABG_02052 |
| Phosphofructokinase 1 | 0.0286 | 0.6565 | 0.6475 | * | PAAG_01583; PABG_03640; PADG_00192 |
| Fructose-1,6-bisphosphatase | 1.5726 | * | 1.9291 | 1.5608 | PAAG_02682; PADG_01706 |
| Fructose-biphosphate aldolase | 1.5640 | * | * | 4.0431 | PAAG_01995; PADG_00852 |
| Triosephosphate isomerase | * | * | 2.1992 | * | PADG_06906 |
| Glyceraldehyde-3-phosphate dehydrogenase | 1.5272 | 1.5622 | 2.2502 | * | PAAG_08468; PABG_00022; PADG_02411 |
| Phosphoglycerate kinase | * | 0.4094 | 0.2854 | * | PABG_03332; PADG_01896 |
| Phosphoglycerate mutase | * | * | 1.7510 | * | PADG_05109 |
| 6-phosphofructo-2-kinase | 0.6565 | * | * | * | PAAG_03643 |
| Fructose-2,6-bisphosphatase | * | * | * | 0.2902 | PADG_07202 |
|  |  |  |  |  |  |
| **Ethanol production** | | | | | |
| Aldehyde dehydrogenase A | * | * | * | 2.18 | PADG_03403 |
| Aldehyde dehydrogenase | 1.7035 | * | * | * | PAAG_03910 |
| Pyruvate decarboxylase | 1.6585 | * | * | 1.5160 | PAAG_02050; PADG_00714 |
| Alcohol dehydrogenase 1 | 5.8833 | 2.6590 | 1.8945 | 3.5222 | PAAG_00403; PABG_04316; PADG_11405 |
| Alcohol dehydrogenase | * | 2.8854 | 4.6278 | 4.6365 | PABG_04316; PADG_04701 |
| Alcohol dehydrogenase | 2.3540 | 0.4684 | * | * | PAAG_08911; PABG_07767 |
|  |  |  |  |  |  |
| **Citrate cycle (TCA cycle)** | | | | | |
| Acetyl-CoA hydrolase | * | * | 0.5468 | * | PADG_02597 |
| Acetyl-coenzyme A synthetase | * | * | 1.5934 | * | PADG_01677 |
| Aconitate hydratase | 2.5739 | 1.8855 | * | 2.0604 | PAAG_05048; PABG_11957; PADG_11845 |
| ATP-citrate synthase subunit 1 | * | 3.9958 | 2.0159 | 0.5420 | PABG_04594; PADG_04993 |
| ATP-citrate-lyase | * | 0.5640 | * | 1.5492 | PABG_04595; PADG_04994 |
| Citrate lyase subunit beta | * | * | 2.7667 | * | PADG_01546 |
| Citrate synthase | 1.9503 | * | * | 2.1828 | PAAG_08075; PADG_08387 |
| Dihydrolipoamide succinyltransferase | 2.1272 | * | * | * | PAAG_08915 |
| Dihydrolipoyl dehydrogenase | 0.0395 | * | * | 1.6093 | PAAG_03330; PADG_06494 |
| Fumarate hydratase | 2.0430 | * | 3.2527 | 1.7921 | PAAG_00588; PADG_08119 |
| Fumarate reductase | * | * | * | 1.7370 | PADG_02592 |
| Isocitrate dehydrogenase [NAD] subunit 1 | 1.5573 | 1.6896 | * | 1.6513 | PAAG_00856; PABG_01382; PADG_03977 |
| Malate dehydrogenase | 1.5893 | * | * | 1.6212 | PAAG_00053; PADG_07210 |
| Oxoglutarate dehydrogenase | 1.6015 | 1.5253 | 1.6444 | 1.5575 | PAAG_02732; PABG_03210; PADG_01762 |
| Pyruvate dehydrogenase complex dihydrolipoamide acetyltransferase | 1.6615 | * | 1.8264 | 0.4023 | PAAG_11035; PADG_07213 |
| Pyruvate dehydrogenase kinase | * | * | 3.6490 | * | PADG_12250 |
| Succinate dehydrogenase [ubiquinone] flavoprotein subunit | 3.4568 | 2.6561 | * | 2.2497 | PAAG_01725; PABG_03772; PADG_00052 |
| Succinate dehydrogenase [ubiquinone] iron-sulfur subunit | 3.2987 | * | * | 4.2371 | PAAG_06103; PADG_06553; PADG_08013 |
| Succinyl-CoA sintetase subunit alpha | 1.7090 | * | 0.4054 | 1.5504 | PAAG_00417; PADG_02260 |
|  |  |  |  |  |  |
| **Glyoxylate cycle** | | | | | |
| Isocitrate lyase | 2.9921 | * | * | 1.9860 | PAAG_06951; PABG_01483 |
| Malate synthase | 1.7279 | * | * | 2.1061 | PAAG_04542; PADG_04702 |
|  |  |  |  |  |  |
| **Oxidation of fatty acids** | | | | | |
| 3-hydroxybutyryl-CoA dehydrogenase | 2.4003 | * | 1.8680 | 1.7645 | PAAG_06329; PADG_01228 |
| 3-ketoacyl-CoA thiolase | 2.1333 | 1.5187 | 1.7094 | 1.7562 | PAAG_02664; PABG_00747; PADG_01687 |
| 3-ketoacyl-CoA thiolase B, peroxisomal | * | * | * | 0.5984 | PADG_03194 |
| 60 kDa lysophospholipase | * | * | 0.5333 | * | PADG_05733 |
| Acyl-CoA dehydrogenase | 2.0032 | 1.7105 | * | 2.4653 | PAAG_05454; PABG_01791; PADG_06805 |
| Acyl-coenzyme A desidrogenase | 2.4629 | * | 1.5613 | * | PAAG_03116; PADG_02991 |
| Carnitine O-acetyltransferase | * | * | * | 2.1491 | PADG_07023 |
| Delta(3,5)-Delta(2,4)-dienoyl-CoA isomerase | * | 0.5139 | * | 1.8556 | PABG_07087; PADG_06721 |
| Enoyl-CoA hydratase | 1.5675 | 1.7676 | * | 2.0790 | PAAG_06309; PABG_03016; PADG_01209 |
| Enoyl-CoA hydratase/isomerase family protein | 1.5699 | * | * | 0.5033 | PAAG_06392; PADG_01291 |
| Short chain dehydrogenase family protein | * | * | * | 2.4576 | PADG_02527 |
| Short-chain dehydrogenase | 4.7118 | * | 0.1428 | * | PAAG_01557; PADG_00221 |
|  |  |  |  |  |  |
| **Synthesis and degradation of ketone bodies** | | | | | |
| Acetyl-CoA acetyltransferase | 2.1667 | * | 1.5126 | * | PAAG_03447; PADG_02751 |
| Hydroxymethylglutaryl-CoA synthase | * | * | 0.6010 | * | PADG_00685 |
| Hydroxymethylglutaryl-CoA lyase | * | * | 1.9153 | 2.2882 | PADG_07031 |
| Succinyl-CoA:3-ketoacid-coenzyme A transferase | 1.6768 | * | 1.5178 | 2.2355 | PAAG_05093; PADG_04939 |
| Acetoacetate-CoA ligase | * | 0.4800 | 0.1778 | * | PABG_03423; PADG_01993 |
|  |  |  |  |  |  |
| **Fatty acid biosynthesis** | | | | | |
| 3-oxoacyl-[acyl-carrier-protein] reductase | 0.2060 | * | * | 2.3929 | PAAG_03123; PADG_06721 |
| Acyl-coenzyme A synthetase | * | * | 0.5963 | 0.6245 | PADG_04827 |
| Fatty acid synthase subunit alpha | * | * | 4.0867 | 0.5526 | PADG_00254 |
| Fatty acid synthase subunit beta dehydratase | 0.3496 | 0.5839 | 0.2939 | 0.5910 | PAAG_01524; PABG_03585; PADG_00255 |
| Long-chain-fatty-acid-CoA ligase | * | * | * | 0.5856 | PADG_00434 |
| Trans-2-enoyl-CoA reductase | * | * | 0.5134 | 2.3908 | PADG_00244 |
|  |  |  |  |  |  |
| **Methylcitrate cycle** | | | | | |
| 2-methylcitrate synthase | 2.6721 | * | 2.3207 | 2.6157 | PAAG_04550; PADG_04710 |
| 2-methylcitrate dehydratase | 2.0037 | * | * | 1.8910 | PAAG_04559; PADG_04718 |
| Methylisocitrate lyase | * | 2.5429 | 2.9785 | 1.6659 | PABG_04323; PADG_04709 |
| Propionate-CoA ligase | * | 1.5638 | 2.0471 | 3.5024 | PABG_04660; PADG_05281 |
|  |  |  |  |  |  |
| **Electron transport and membrane-associated energy conservation** | | | | | |
| NADH-ubiquinone oxidoreductase 21.3 kDa subunit | * | * | 1.5948 | 2.0924 | PADG_05343 |
| Alternative oxidase, mitochondrial | * | * | * | 3.5486 | PADG_03747 |
| ATP synthase delta chain | * | 3.2513 | * | 1.5543 | PABG_06161; PADG_07042 |
| ATP synthase F1, gamma subunit | 2.1728 | * | 1.9140 | 1.8508 | PAAG_05576; PADG_07813 |
| ATP synthase subunit 4 | * | * | * | 1.9560 | PADG_02578 |
| ATP synthase subunit alpha | 0.0678 | 1.6317 | 1.9289 | 3.4632 | PAAG_04820; PABG_11057; PADG_02561 |
| ATP synthase subunit beta | 4.0577 | 1.8668 | * | 4.4407 | PAAG_08037; PABG_07285; PADG_08349 |
| ATP synthase subunit D | * | * | 0.5723 | 2.2771 | PADG_04729 |
| ATP synthase subunit delta | 4.2276 | 1.9723 | * | 4.9575 | PAAG_05605; PABG_06178;PADG_07789 |
| ATP synthase subunit G | 2.4386 | * | * | * | PAAG_08551 |
| Cytochrome b5 | * | 1.6289 | * | * | PABG_04666 |
| Cytochrome b-c1 complex subunit 2 | 3.1252 | * | * | 3.7722 | PAAG_08088; PADG_08394 |
| Cytochrome c | 1.5212 | * | 0.2200 | 3.2504 | PAAG_06268; PADG_06978 |
| Cytochrome c oxidase assembly protein COX19 | * | * | 0.3454 | 1.9027 | PADG_01841 |
| Cytochrome c oxidase polypeptide VIb | * | 1.7915 | * | 1.5998 | PABG_05268; PADG_08292 |
| NADH-ubiquinone oxidoreductase 49 kDa subunit, mitochondrial | * | 1.5429 | * | 2.6265 | PABG_12311; PADG_12148 |
| NADPH dehydrogenase | * | * | 2.7925 | * | PADG_01519 |
| Putative cytochrome c oxidase subunit VIa | * | * | 1.8851 | * | PADG_05750 |
| Ubiquinol-cytochrome c reductase subunit 7 | * | * | 5.8276 | * | PADG_04501 |
|  |  |  |  |  |  |
| **2. CELL WALL** | | | | | |
| Chitin synthase class V | * | * | 4.8078 | * | PADG_07913 |
| Chitin biosynthesis protein | * | 0.6085 | * | * | PABG_06519 |
| Glucan 1,3-beta-glucosidase | * | 1.5845 | * | * | PABG_06330 |
| Glucosamine-fructose-6-phosphate aminotransferase | 0.0947 | * | 0.1893 | 0.1098 | PAAG_00850; PADG_03984 |
| Sterol 24-C-methyltransferase | * | * | 0.5381 | * | PADG_00204 |
| Glucosamine-6-phosphate deaminase | * | * | * | 1.5413 | PADG_00401 |
| Phosphoacetylglucosamine mutase | * | * | * | 1.6978 | PADG_00604 |
| UDP-N-acetylglucosamine pyrophosphorylase | * | * | * | 1.5221 | PADG_04312 |
|  |  |  |  |  |  |
| **3. AMINO ACID DEGRADATION** | | | | | |
| **Methionine, threonine, valine leucine and isoleucine** | | | | | |
| 2-oxoisovalerate dehydrogenase subunit beta | 1.6422 | 0.5631 | * | * | PAAG_01194; PABG_01773 |
| 3-hydroxyisobutyrate dehydrogenase | * | * | * | 1.5232 | PADG_03466 |
| Adenosylhomocysteinase | 1.9887 | * | 1.9063 | 1.9792 | PAAG_02859; PADG_01886 |
| Branched-chain amino acid aminotransferase | 0.2930 | 0.5525 | 0.4502 | 1.8625 | PAAG_04401 |
| Cystathionine beta-synthase | * | 0.5917 | * | * | PABG_04676 |
| Cystathionine gamma-lyase | * | * | 2.1645 | * | PADG_02456 |
| Dihydrolipoamide branched chain transacylase | * | 0.3502 | * | * | PABG_03242 |
| Isovaleryl-CoA dehydrogenase | * | * | 1.6305 | * | PADG_07369 |
| Methylcrotonoyl-CoA carboxylase beta chain | 1.5500 | * | * | 1.7011 | PAAG_04103; PADG_07370 |
| Methylcrotonoyl-CoA carboxylase subunit alpha | * | * | 1.9142 | * | PADG_07366 |
| Methylmalonate-semialdehyde dehydrogenase | 1.6596 | * | * | * | PAAG_07036 |
| Mitochondrial methylglutaconyl-CoA hydratase | * | * | * | 2.3069 | PADG_00643 |
| Threonine dehydratase | 2.3523 | * | * | * | PAAG_03168 |
|  |  |  |  |  |  |
| **Threonine, alanine, glycine and serine** | | | | | |
| Alanine aminotransferase | 1.7302 | * | * | * | PAAG_08207 |
| Alanine-glyoxylate aminotransferase | * | 2.4552 | * | * | PABG_00589 |
| Glycine cleavage system H protein | * | * | * | 1.5237 | PADG_01963 |
| Glycine cleavage system T protein | * | * | * | 0.5432 | PADG_02914 |
| Glycine dehydrogenase | 1.5611 | 1.9209 | 0.3708 | 2.7044 | PAAG_1568; PABG_04990; PADG_00210 |
| L-serine dehydratase | * | * | 3.0201 | * | PADG_11705 |
| Serine hydroxymethyltransferase | * | 0.5413 | * | * | PABG_07147 |
| Threonine dehydratase | * | 4.0242 | * | * | PABG_01280 |
|  |  |  |  |  |  |
| **Aspartate** | | | | | |
| Aspartate aminotransferase | 0.9755 | 1.9558 | 0.3257 | 0.4548 | PABG_02806; PADG_01404 |
|  |  |  |  |  |  |
| **Arginine, histidine, glutamate and proline** | | | | | |
| Arginase | * | * | 2.8753 | 1.5659 | PADG_00637 |
| 1-pyrroline-5-carboxylate dehydrogenase | 3.7092 | * | 1.8568 | 3.1996 | PAAG_05253, PADG_05085 |
| 4-aminobutyrate aminotransferase | * | * | 1.6145 | * | PADG_02214 |
| Amino-acid acetyltransferase | * | 1.5500 | * | * | PABG_11192 |
| Glutamate decarboxylase | 0.3816 | * | * | * | PAAG_03506 |
| Glutamate dehydrogenase | * | * | 2.6447 | * | PADG_04516 |
| Glutamate-5-semialdehyde dehydrogenase | 1.7370 | 2.0399 | 6.9476 | * | PAAG_07954; PABG_04712; PADG_05337 |
| Glutamine synthetase | * | 1.8440 | 0.0060 | 0.4642 | PABG_03010; PADG_01536 |
| Ornithine aminotransferase | * | 2.6378 | * | 1.6393 | PABG_02827; PADG_01328 |
| Succinate-semialdehyde dehydrogenase | * | * | 2.3322 | * | PADG_03058 |
|  |  |  |  |  |  |
| **Phenylalanine, tyrosine, lysine and tryptophan** | | | | | |
| 4-hydroxyphenylpyruvate dioxygenase | * | 4.0525 | 1.6926 | * | PABG_07394; PADG_08468 |
| Fumarylacetoacetase | 1.8464 | * | * | 1.6096 | PAAG_08163; PADG_08465 |
| Glutaryl-CoA dehydrogenase | 1.5648 | * | * | 1.5538 | PAAG_05984; PADG_12025 |
| Homogentisate 1,2-dioxygenase | 2.8449 | 3.9693 | * | 2.9274 | PAAG_08164; PABG_07392; PADG_08466 |
| Kynureninase | * | 1.9978 | * | 2.1469 | PABG_01965; PADG_00349 |
| Kynurenine-oxoglutarate transaminase | 3.3878 | 2.1199 | * | * | PAAG_02644; PABG_03129 |
| Maleylacetoacetate isomerase | 1.8658 | 2.6612 | * | * | PAAG_08162; PABG_07390 |
| Phenylpyruvate tautomerase | * | * | 2.0329 | * | PADG_03671 |
|  |  |  |  |  |  |
| **4. STRESS RESPONSE / DETOXIFICATION** | | | | | |
| Cytochrome c peroxidase | * | 3.2024 | 0.4209 | * | PABG_00720; PADG_03163 |
| Glutamate-cysteine ligase | 5.5711 | * | 0.4408 | 0.5170 | PAAG_05860; PADG_03500 |
| Glutathione peroxidase | * | 3.6064 | * | * | PABG_04219; |
| Glutathione synthetase | * | * | 0.3462 | * | PADG_02218 |
| Heat shock 70 kd protein cognate 1 | * | * | 2.1446 | * | PADG_05139 |
| Heat shock protein 30 kDa | 3.4272 | * | * | 2.3727 | PAAG_00871; PADG_03963 |
| Heat shock protein Hsp88 | * | 1.7109 | 2.2386 | * | PABG_00374; PADG_02785; |
| Heat shock protein STI1 | * | * | 0.3898 | * | PADG_04379 |
| Hsp70 | * | * | 1.7698 | 0.5490 | PADG_00778 |
| Hsp75-like protein | 0.6087 | * | * | 0.6386 | PAAG_07775; PADG_02761 |
| Hsp90-like protein | 0.0729 | * | 0.1264 | 0.1902 | PAAG_05679; PADG_07715 |
| Peroxisomal catalase P | 2.7216 | 7.8608 | 3.5746 | 1.9969 | PAAG_01454; PADG_00324; PABG_01943 |
| Subtilase-type proteinase psp3 | * | 1.6536 | * | * | PABG_03958 |
| Superoxide dismutase SOD1 Cu/Zn | 3.3803 | 2.1639 | 2.2682 | 2.4857 | PAAG_04164; PABG_03954; PADG_07418 |
| Superoxide dismutase SOD2 Fe/Mn | 3.7060 | 2.5223 | 2.2353 | 3.1283 | PAAG_02725; PABG_03204; PADG_01755 |
| Superoxide dismutase SOD3 Cu/Zn | * | 1.5782 | * | * | PABG_00431 |
| Superoxide dismutase SOD4 Cu/Zn | * | * | 1.7988 | * | PADG_01400 |
| Superoxide dismutase SOD5 Fe/Mn | 2.5451 | 1.5782 | 1.7258 | 2.2816 | PAAG_02926 PABG_03387 PADG_01954 |
| Thioredoxin | * | * | * | 0.5506 | PADG_03161 |
| Thioredoxin reductase | * | 0.5212 | 1.6851 | 0.5843 | PABG_03023; PADG_01551 |

^a^Biological process of differentially expressed proteins from MIPS (http://mips.helmholtz-muenchen.de/funcatDB/) and Uniprot databases (<http://www.uniprot.org/>).

^b^Proteins annotation from *Paracoccidioides* genome database or by homology from NCBI database (<http://www.ncbi.nlm.nih.gov/>).

^c^Acetate/Glucose means: The level of expression in yeast cells derived from cultured in sodium acetate divided by the level in the control yeast cells cultured in glucose, in the differents isolates: *P. lutzii* (*Pb*01) and *P. brasiliensis* (*Pb*03, *Pb*339, and *Pb*EPM83).

^d^Identification of differentially regulated proteins from *Paracoccidioides* genome database (http://www.broadinstitute.org/annotation/genome/paracoccidioides_brasiliensis/MultiHome.html) using the ProteinLynx Global Server vs. 2.4 (PLGS) (Waters Corporation, Manchester, UK).

*Constitutive protein or not regulated.
