# Supplementary material for: Differential Metabolism of a Two-Carbon Substrate by Members of the Paracoccidioides Genus
Source: Front Microbiol. 2017 Nov 27;8:2308. doi: 10.3389/fmicb.2017.02308 (PMC5711815; doi:10.3389/fmicb.2017.02308)
Supplement: Supplementary file 11 [file Image1.PDF]

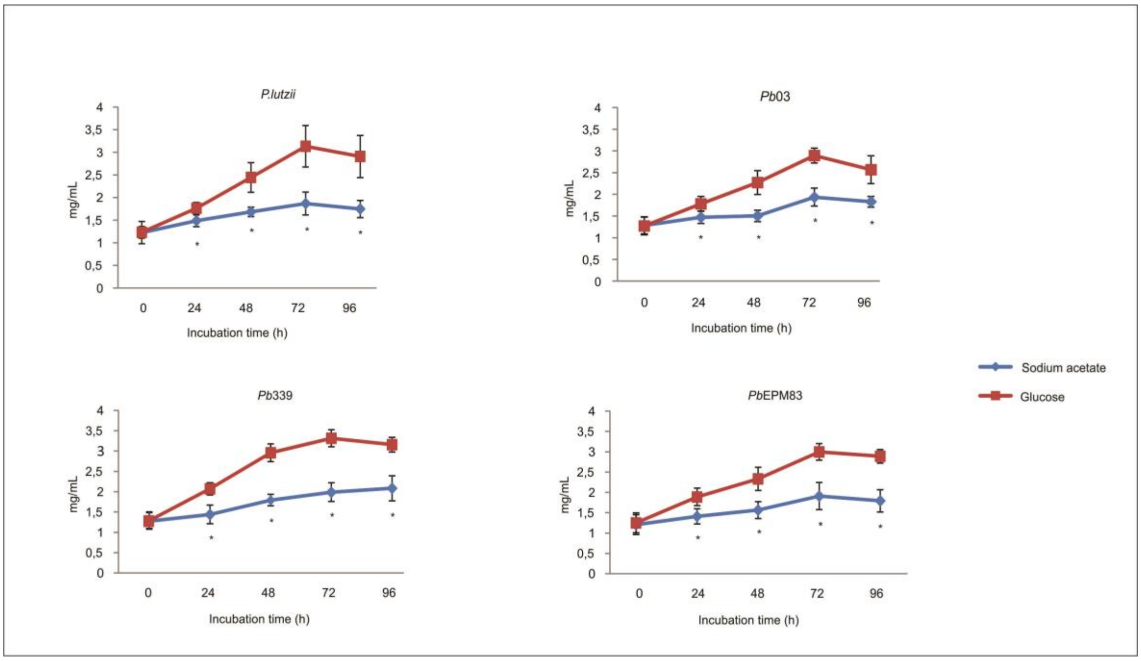

Supplemental Figure 1: Growth of *P. lutzii* (*Pb*01) **and** *P. brasiliensis* **isolates** *Pb*03, *Pb*339, **and** *Pb*EPM83 **in glucose and sodium acetate as carbon sources**. A total of $1x10^6$ cells/50 mL were incubated in MMcM medium with glucose (100 mM) or sodium acetate (100 mM) for 96 hours. Cells were collected at time intervals, killed by heat, lyophilized and the dry weight was determined. Data are expressed as the mean ± standard deviation of the triplicates of independent experiments. Student's *t-test* was used.*, significantly different from in glucose condition, at *p*-value of ≤ 0.05.
