# Supplementary material for: Differential Metabolism of a Two-Carbon Substrate by Members of the Paracoccidioides Genus
Source: Front Microbiol. 2017 Nov 27;8:2308. doi: 10.3389/fmicb.2017.02308 (PMC5711815; doi:10.3389/fmicb.2017.02308)
Supplement: Supplementary file 12 [file Image2.PDF]

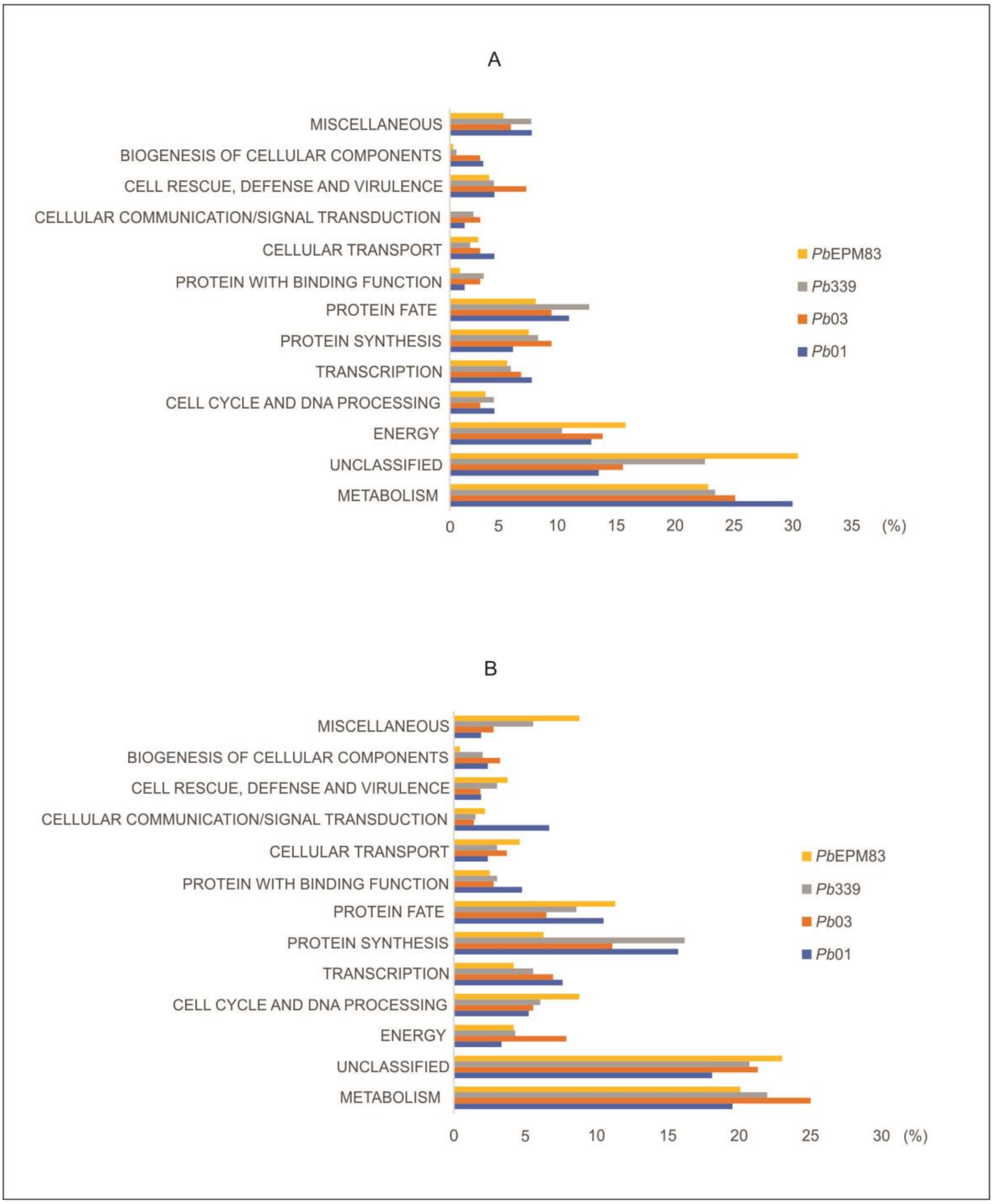

Supplemental Figure 2: Functional classification of proteins regulated in yeast cells of *P.lutzii* **and** *P. brasiliensis* **grown in 100mM sodium acetate as carbon source for 48 hours and obtained by NanoUPLC-MS^{E} analysis**. **(A)** Biological processes of induced proteins. **(B)** Biological processes of repressed proteins. The biological processes of the differentially expressed proteins in the isolates were obtained using the Pedant in MIPS for *P. lutzii* (http://pedant.helmholtzmuenchen.de/pedant3htmlview/pedant3view?Method=analysis&Db=p3_r48325_Par_lutzi), *Pb*03 (http://pedant.helmholtzmuenchen.de/pedant3htmlview/pedant3view?Method=analysis&Db=p3_p27779_Par_brasi_Pb03). and *Pb339*. *Pb*EPM83 (http://pedant.helmholtzmuenchen.de/pedant3htmlview/pedant3view?Method=analysis

&Db=p3_p28733_Par_brasi_Pb18), Uniprot (http://www.uniprot.org/) and KEGG: Kyoto Encyclopedia of Genes and Genomes (www.genome.jp/kegg/).
