# Supplementary material for: Differential Metabolism of a Two-Carbon Substrate by Members of the Paracoccidioides Genus
Source: Front Microbiol. 2017 Nov 27;8:2308. doi: 10.3389/fmicb.2017.02308 (PMC5711815; doi:10.3389/fmicb.2017.02308)
Supplement: Supplementary file 13 [file Image3.PDF]

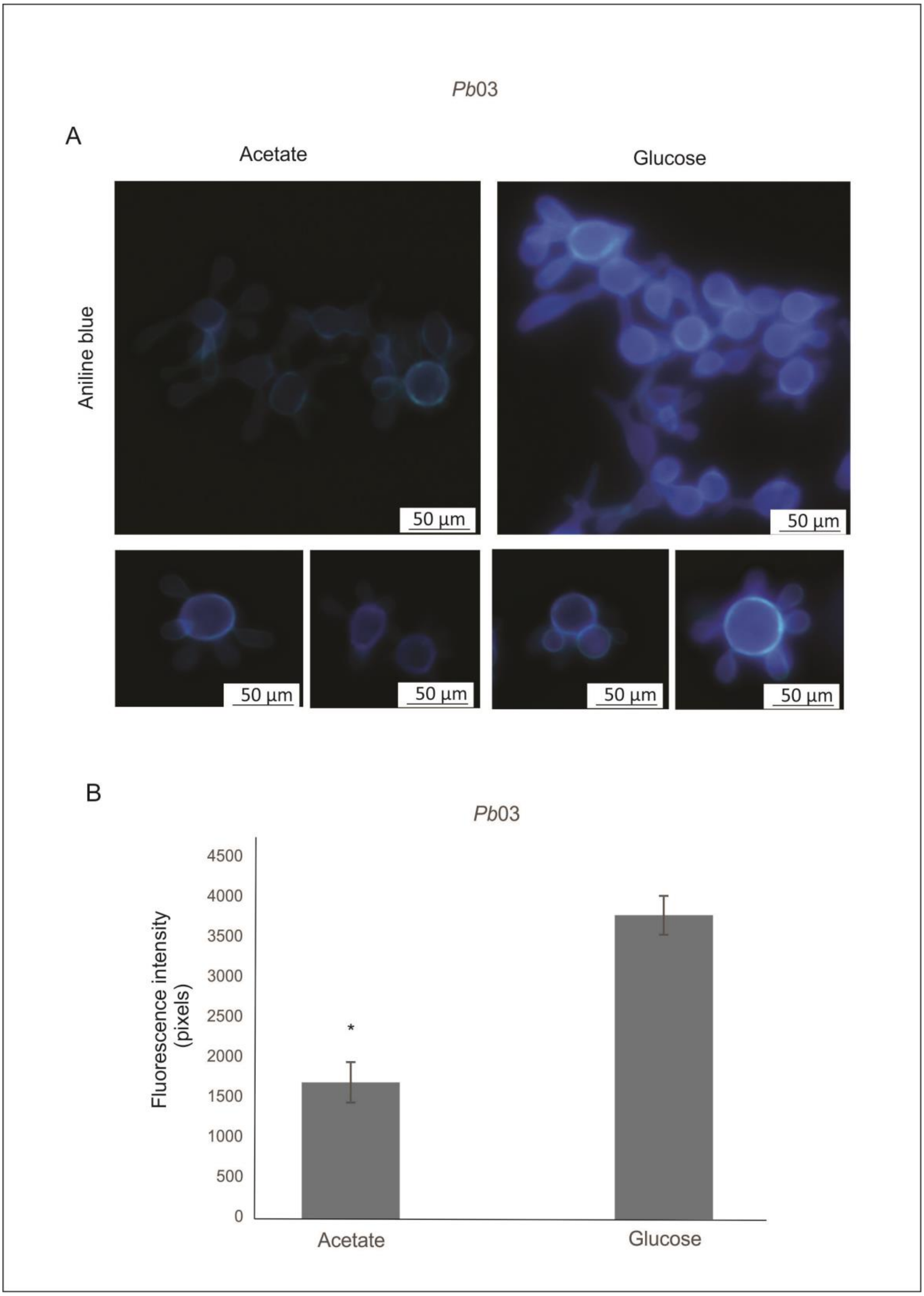

Supplemental Figure 3: Evaluation of β-1,3 glucan quantities in the cell wall of *P. brasiliensis* **isolate** *Pb*03**. (A)** Aniline blue was used to evaluate, by fluorescence microscopy, the presence of β-1,3 glucan in the cell wall of *Pb*03, after growth in MMcM medium with sodium acetate or glucose for 48 h. **(B)** Fluorescence intensity graph. The values of fluorescence intensity (in pixels) and the standard error of each analysis were

used to plot the graph. Data are expressed as mean ± standard error (represented using error bars). (*) represents $p \leq 0.05$.
