# Supplementary material for: Differential Metabolism of a Two-Carbon Substrate by Members of the Paracoccidioides Genus
Source: Front Microbiol. 2017 Nov 27;8:2308. doi: 10.3389/fmicb.2017.02308 (PMC5711815; doi:10.3389/fmicb.2017.02308)
Supplement: Supplementary file 14 [file Image4.pdf]

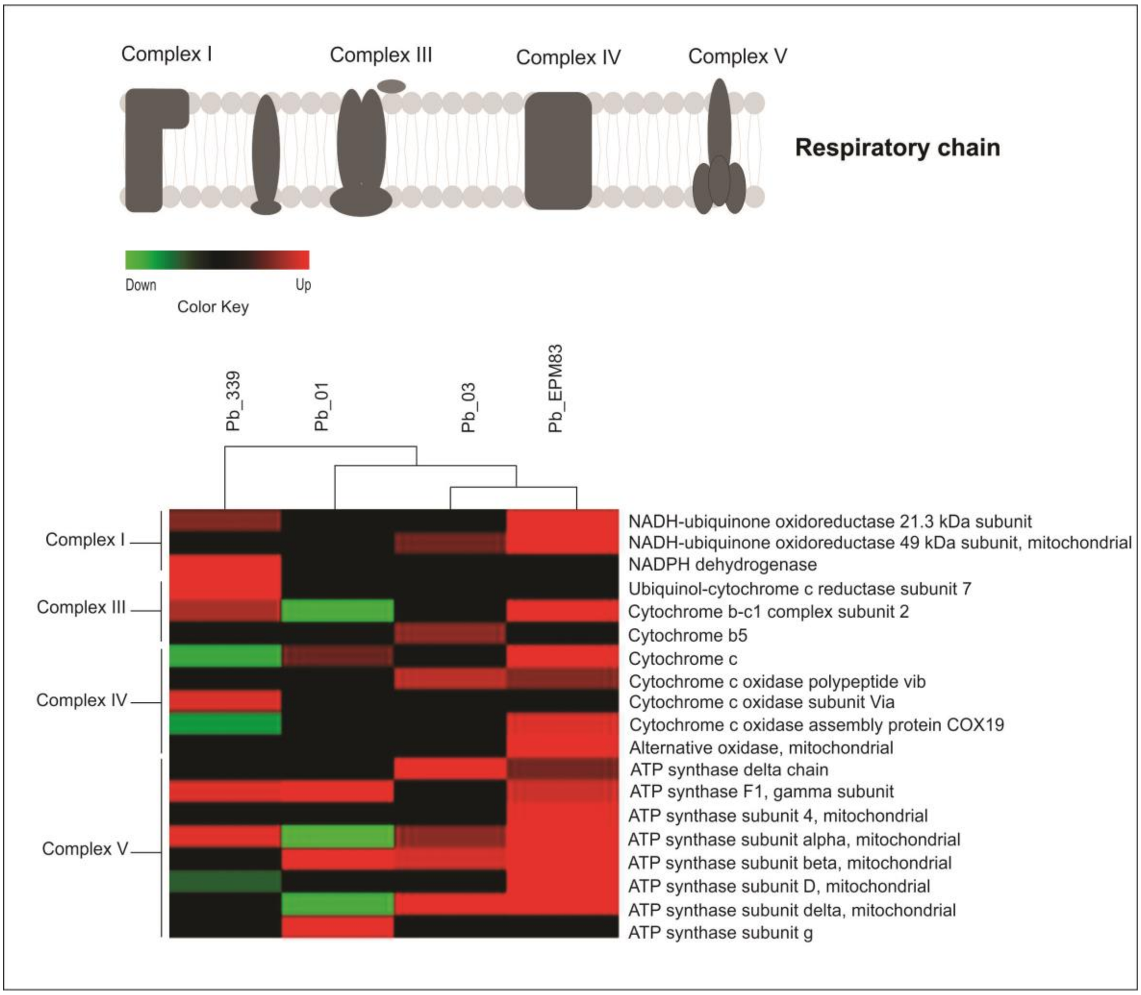

Supplemental Figure 4: Comparison of protein profiles related to electron transport and membrane-associated energy conservation in *P. lutzii* and isolates of *P. brasiliensis*. Data of expression set comparisons were carried out with the MultiExperiment Viewer software V.4.8 (www.tm4.org/mev/) that was used to group and compare data of expression and ANOVA test, applying a cut off at 1.5-fold expression changes. Representative diagram of the electron transport and membrane-associated energy conservation in pathways depicting down regulated (green) and up regulated (red) in the isolates. Black means that no significant difference was observed. Changes in expression levels upon yeast cells incubated with acetate in *Pb*01, *Pb*03, *Pb*339 and *Pb*EPM83 in presence of sodium acetate 100 mM are represented in a heat map format. Mean values of experimental triplicates are shown for down regulation (green) and up regulation (red).
