# Supplementary material for: Differential Metabolism of a Two-Carbon Substrate by Members of the Paracoccidioides Genus
Source: Front Microbiol. 2017 Nov 27;8:2308. doi: 10.3389/fmicb.2017.02308 (PMC5711815; doi:10.3389/fmicb.2017.02308)
Supplement: Supplementary file 15 [file Image5.PDF]

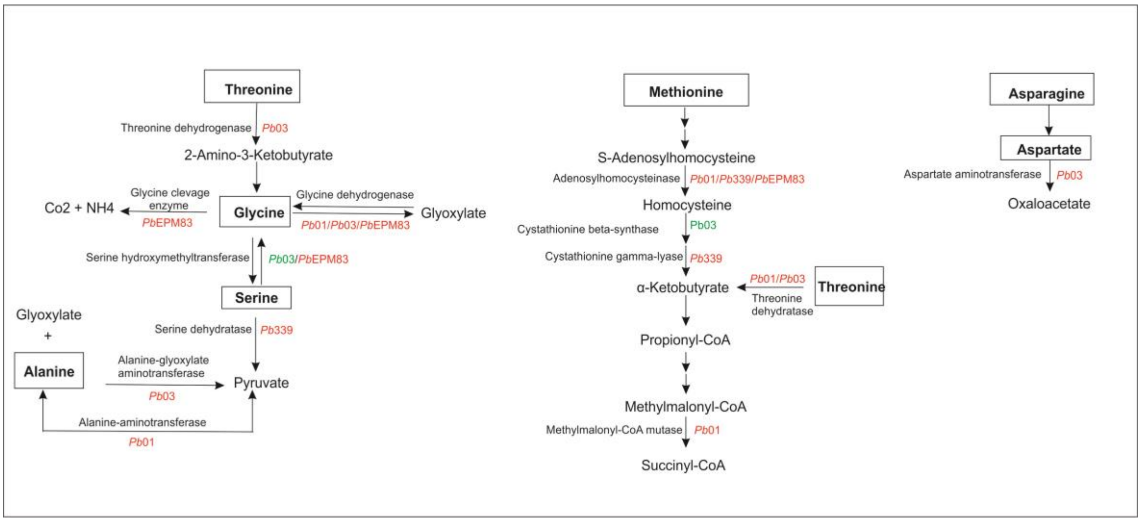


**Supplemental Figure 5: Schematic representation of the degradation of the amino acids methionine, threonine, glycine, serine, alanine and aspartate in** *P. lutzii* (*Pb*01) **and** *P. brasiliensis Pb*03, *Pb*339 and *Pb*EPM83 **in presence of sodium acetate 100 mM**. Proteins up regulated and down regulated in each isolate were depicted by colors red and green, respectively.
