# Supplementary material for: Differential Metabolism of a Two-Carbon Substrate by Members of the Paracoccidioides Genus
Source: Front Microbiol. 2017 Nov 27;8:2308. doi: 10.3389/fmicb.2017.02308 (PMC5711815; doi:10.3389/fmicb.2017.02308)
Supplement: Supplementary file 16 [file Image6.PDF]

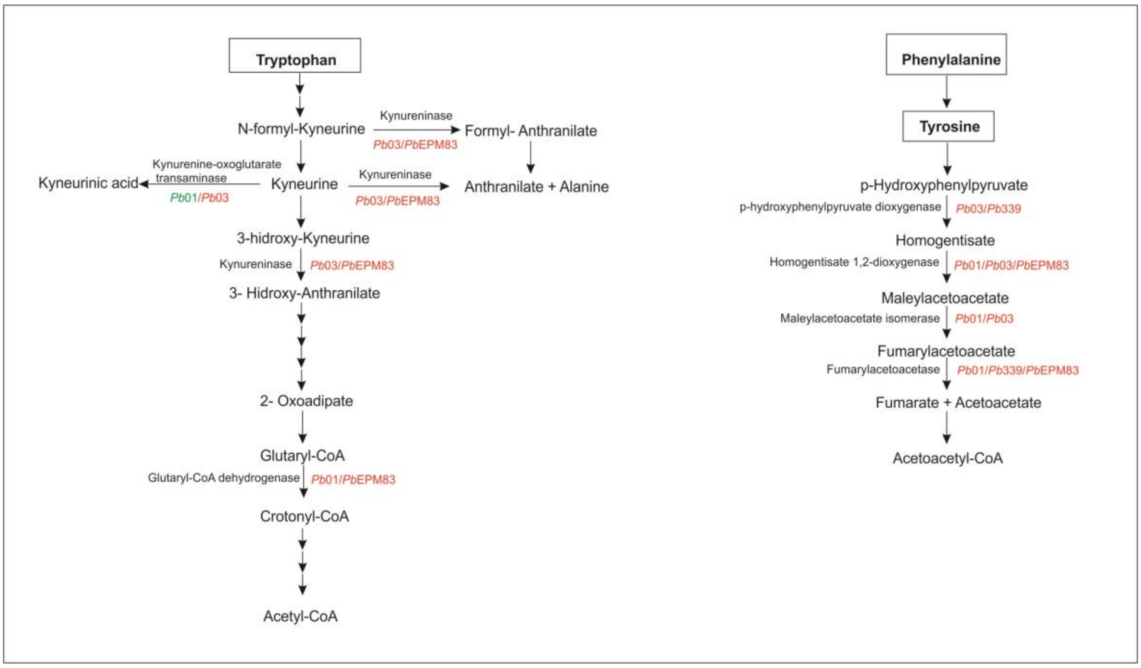

Supplemental Figure 6: Schematic representation of the degradation of the amino acids tryptophan, phenylalanine and tyrosine in *P. lutzii* (*Pb*01) **and** *P. brasiliensis Pb03, Pb339,* **and** *Pb*EPM83 **in presence of sodium acetate 100 mM.** Proteins up regulated and down regulated in each isolate were depicted by colors red and green, respectively.
