# Supplementary material for: Differential Metabolism of a Two-Carbon Substrate by Members of the Paracoccidioides Genus
Source: Front Microbiol. 2017 Nov 27;8:2308. doi: 10.3389/fmicb.2017.02308 (PMC5711815; doi:10.3389/fmicb.2017.02308)
Supplement: Supplementary file 19 [file Image9.pdf]

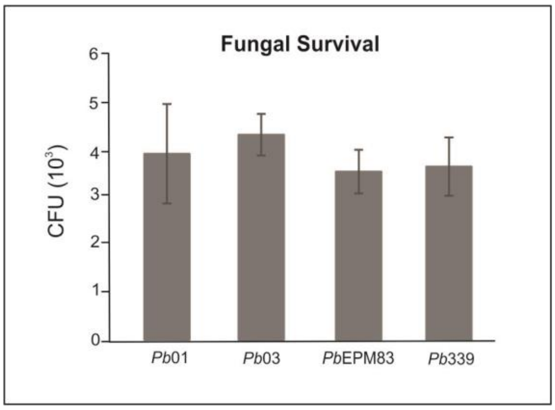

Supplemental Figure 9: Survival of *Paracoccidioides* isolates in macrophages during infection. *Paracoccidioides* yeast cells of the four isolates (*Pb*01, *Pb*03, *Pb*339 and *Pb*EPM83) were previously grown in BHI medium liquid supplemented with 4% (w/v) glucose up to 48 h and then were incubated with macrophages at a 1:5 macrophages: yeast cells ratio, for 24 h.The number of viable cells was determined by quantifying the number of colony forming units/mL (CFUs/mL) during infection from culture supernatant (non-internalized cells removed by aspiration prior to macrophages lysis) and after internalization. Data were expressed as mean ± standard error (represented using error bars) of the biological triplicates of independent experiments, using analysis of variance (ANOVA). There was no significant difference between the four isolates evaluated, for *p*-value of ≤0.05.
